# RAD54L coordinates the nucleolar DNA damage response to maintain rDNA stability

**DOI:** 10.1038/s44318-026-00864-3

**Published:** 2026-07-23

**Authors:** Ruofei Liu, Jiachen Xuan, Carmelo Cerra, Nyree Stojnic, Junqi Pan, Shalini S Chelliah, Shannon Mendez, Rhynelle Dmello, Karla J Cowley, Yangyi Zhang, Kezia Gitareja, Matthew Wakefield, Andrew Deans, Clare L Scott, Keefe T Chan, Kaylene J Simpson, Jian Kang, Elaine Sanij

**Affiliations:** 1 St Vincent’s Institute of Medical Research, Melbourne, VIC Australia; 2 Sir Peter MacCallum Department of Oncology, University of Melbourne, Melbourne, VIC Australia; 3 The Walter and Eliza Hall Institute of Medical Research, Melbourne, VIC Australia; 4 Department of Biochemistry and Molecular Biology, Monash University, Clayton, VIC Australia; 5 Victorian Centre for Functional Genomics, Peter MacCallum Cancer Centre, Melbourne, VIC Australia; 6 Cancer Research Division, Peter MacCallum Cancer Centre, Melbourne, VIC Australia; 7 Department of Medicine- St Vincent’s Hospital, University of Melbourne, Melbourne, VIC Australia; 8 Department of Obstetrics, Gynaecology and Newborn Health, University of Melbourne, Melbourne, VIC Australia; 9 Present Address: The ACRF Department of Cancer Biology and Therapeutics, The John Curtin School of Medical Research, Australian National University, Canberra, ACT Australia

**Keywords:** Chromatin, Transcription & Genomics, DNA Replication, Recombination & Repair

## Abstract

The nucleolus is organized around actively transcribed ribosomal RNA genes (rDNA), where high RNA polymerase I (Pol I) activity creates intrinsic susceptibility to replication stress and DNA damage. Here, we identify the DNA translocase RAD54L as a critical regulator of the nucleolar DNA damage response (nDDR) to rDNA double-strand breaks (DSBs) and replication stress. We show that RAD54L localizes to the nucleolus under basal conditions and is recruited to nucleolar caps following CRISPR-Cas9-induced rDNA-DSBs to promote repair. RAD54L loss results in persistent RAD51 foci, increased nucleolar γH2AX, and micronuclei formation, indicating defective resolution of rDNA lesions and genome instability. Under baseline conditions and replication stress induced by the Pol I transcription inhibitor CX-5461, RAD54L limits the accumulation of ssDNA and coordinates nDDR signaling. We further show that rDNA-DSBs induce RNA polymerase II-dependent RNA-DNA hybrids (R-loops) at intergenic rDNA regions, which facilitate nucleolar reorganization and cap formation and repair factor recruitment. Together, these findings establish RAD54L as a key regulator that coordinates replication stress response and rDNA repair, maintaining rDNA stability and genome integrity.

## Synopsis

The nucleolar DNA damage response (nDDR) preserves the integrity of the highly transcribed and repetitive rDNA repeats. This study identifies RAD54L as a key factor of nDDR, coordinating replication stress response, ATM signaling, and homologous recombination repair to maintain rDNA stability.

- RAD54L localizes to the nucleolus under basal conditions and is recruited to nucleolar caps following CRISPR-Cas9-induced rDNA double-strand breaks (DSBs), where it promotes efficient homologous recombination repair.
- RAD54L coordinates replication stress responses by limiting single-stranded DNA (ssDNA) accumulation and maintaining nucleolar genome stability during basal conditions and following CX-5461-induced replication stress.
- RAD54L promotes coordinated nDDR signalling by supporting ATM-CHK2 activation and nucleolar cap formation during CX-5461-mediated replication stress.
- rDNA damage induces RNA polymerase II-dependent transcription at intergenic rDNA regions, promoting RNA-DNA hybrid (R-loop) formation.
- RNA polymerase II activity is required for nucleolar reorganization and recruitment of DNA repair factors, including RAD54L and TP53BP1, to nucleolar cap structures during repair of rDNA DSBs.

## Abstract (web summary)

rDNA-break-induced RAD54L delocalization to nucleolar caps is important for efficient homologous recombination repair and nucleolar DNA damage responses.

## Graphical abstract

**Figure:**
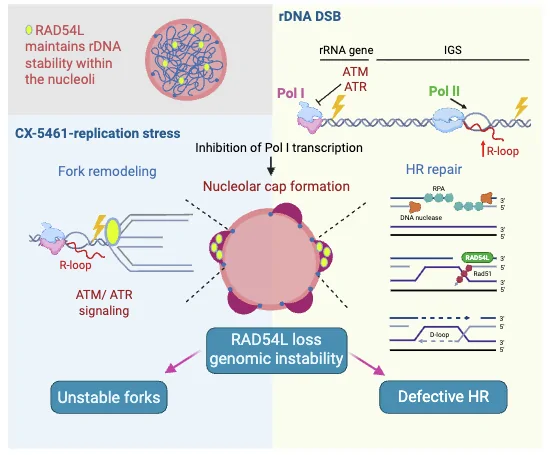

**Figure:**
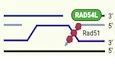

## Introduction

RNA polymerase I (Pol I) transcription of rDNA constitutes more than 60% of all cellular transcription (Schneider, 2012). The rDNA repeats are organized in clusters (ranging from 16 to 76 copies per array), with up to ∼400 copies across the short arm of the five acrocentric chromosomes in human cells (Nurk et al, 2022). Pol I transcribes a subset of the rDNA repeats to produce the 47S precursor-ribosomal RNA (pre-rRNA) within the nucleoli, which serve as the sites of Pol I transcription and ribosomal subunit assembly. The combination of high transcriptional output, repetitive sequence architecture, and dynamic chromatin organization renders rDNA loci intrinsically vulnerable to replication stress and DNA damage. The instability of the rDNA loci is increased upon loss of genome maintenance factors such as BLM and WRN, emphasizing the need for robust surveillance mechanisms to preserve the integrity of rDNA loci (Killen et al, 2009).

Several unique characteristics of the rDNA repeats, including their repetitive nature, high Pol I transcription rates, and high GC content, present risks of genomic instability (Potapova et al, 2019; Xuan et al, 2021). The head-to-tail arrangement of rDNA copies per locus allows recombination events, which can lead to alterations in rDNA copy number and genomic rearrangements (Wang and Lemos, 2017; Warmerdam and Wolthuis, 2019). Furthermore, high levels of transcription render the rDNA clusters vulnerable to transcription-replication conflicts (TRCs), which can promote replication fork stalling and single-strand DNA (ssDNA) accumulation (Saxena and Zou, 2022; Xuan et al, 2021). rDNA double-strand breaks (DSBs) are repaired by the homologous recombination (HR), non-homologous end-joining (NHEJ) repair, and single-strand annealing pathways that are activated depending on the cell cycle stage, the extent of rDNA damage, and persistence of DSBs (Goffova and Fajkus, 2021; Warmerdam and Wolthuis, 2019). Thus, precise coordination of repair pathway choice and replication dynamics is critical for maintaining rDNA stability.

The transit from low to high levels of rDNA damage is associated with ATM- and ATR-mediated inhibition of Pol I transcription and large-scale reorganization of nucleolar architecture that involves localization of rDNA DSBs to nucleolar caps at the periphery (Korsholm et al, 2020; van Sluis and McStay, 2017). This spatial reorganization is thought to facilitate access of repair machinery while limiting aberrant recombination between rDNA arrays on distinct chromosomes (Gal et al, 2024; van Sluis and McStay, 2015). Accompanying this structural reorganization of the nucleoli is a shift from NHEJ to HR type of repair (Korsholm et al, 2020). In agreement with this, HR factors are recruited to nucleolar caps after rDNA damage (Korsholm et al, 2019; Marnef et al, 2019; Mooser et al, 2020; van Sluis and McStay, 2015; Warmerdam et al, 2016), suggesting that nucleolar compartmentalization spatially restricts HR at damaged loci.

Here, we utilized a boutique CRISPR-Cas9 screen to assess the interactions of DNA repair pathways with inhibitors of Pol I transcription and identified a previously unrecognized role for the DNA translocase RAD54L in nucleolar DNA damage response (nDDR). RAD54L is a member of the SWI2/SNF2 family of ATP-dependent DNA translocases and is a central effector of HR. Following DNA end resection, RAD54L interacts with RAD51 nucleoprotein filaments, stimulating homology search and strand invasion, then removing RAD51 from heteroduplex DNA after strand exchange has occurred, thereby enabling completion of repair and preventing persistence of recombination intermediates (Mason et al, 2015). In addition to its established role in HR, emerging evidence has implicated RAD54L in the regulation of replication fork dynamics. RAD54L restrains replication fork progression and promotes fork reversal under conditions of replication stress, limiting the accumulation of pathological replication intermediates (Uhrig et al, 2024).

In this study, we observed that RAD54L localizes to nucleoli in both replicating and non-replicating cells. To interrogate rDNA damage responses, we employed two complementary approaches: targeted induction of rDNA DSBs using a CRISPR-Cas9-based system, and pharmacological induction of nucleolar replication stress using CX-5461, an inhibitor of Pol I transcription (Drygin et al, 2011; Sanij et al, 2020). Upon rDNA DSBs, RAD54L is recruited to nucleolar caps and is required for efficient repair and maintenance of rDNA stability. Under CX-5461-mediated replication stress, RAD54L loss results in increased ssDNA accumulation, altered ATM signaling, and impaired nucleolar cap formation, consistent with defective coordination of responses to rDNA damage and replication stress.

Moreover, rDNA damage induces TRCs associated with RNA-DNA hybrids (R-loops), with our data supporting a contribution from RNA polymerase II (Pol II)-associated transcription at intergenic spacer (IGS) regions of rDNA loci. Inhibition of Pol II activity attenuates the formation of nucleolar caps and the recruitment of DNA repair factors, including TP53BP1 and RAD54L, to rDNA DSBs. Thus, Pol II provides a transcriptional substrate for R-loop formation that facilitates nucleolar reorganization following the induction of rDNA damage. Our data show that RAD54L has both basal and damage-induced roles in the nucleolus. Under unstressed conditions, RAD54L supports replication fork restraint to limit ssDNA and R-loop accumulation. However, following nucleolar stress or rDNA DSBs, RAD54L is recruited to nucleolar caps, where it contributes to the execution of the nDDR. These findings define a dynamic local surveillance mechanism within the nucleolus, in which RAD54L acts to restrain and stabilize replication forks and coordinates ATM-dependent signaling and responses to rDNA damage to preserve rDNA integrity.

## Results

### Loss of RAD54L leads to synthetic lethal interactions with Pol I transcription inhibitors

**Figure 1:**
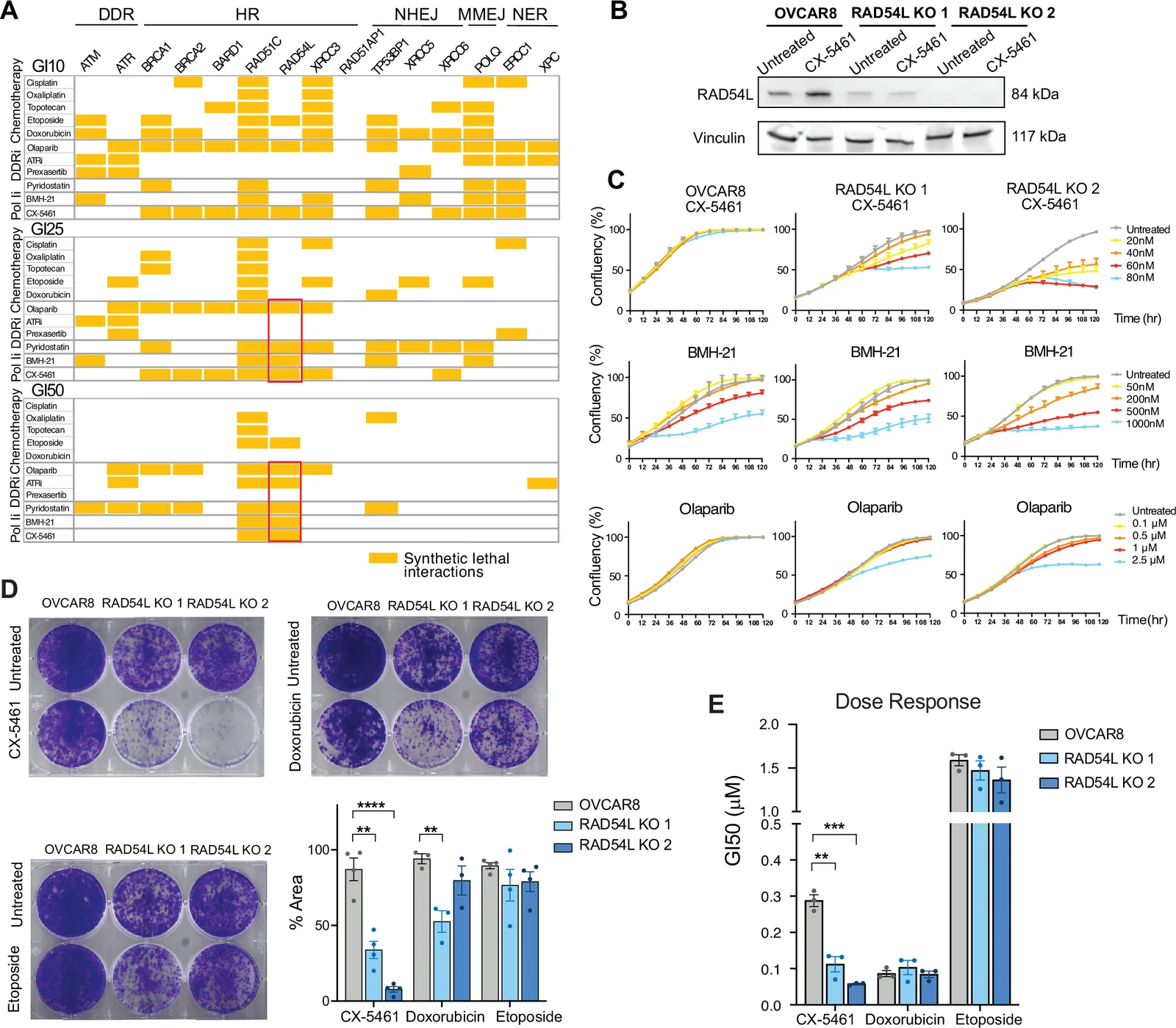
Loss of RAD54L leads to a synthetic lethal interaction with Pol I transcription inhibitors. (**A**) Snapshot of synthetic lethal (SL) interactions between DDR gene knockouts and pharmacological perturbations in OVCAR8 cells. Cells were treated for 72 h with a panel of 11 drugs, including chemotherapeutics, DDR inhibitors (DDRi), and Pol I transcription inhibitors, at three doses per drug, with six technical replicates per condition. SL interactions were quantified by calculating both the absolute growth difference and the relative growth difference for each gene knockout compared with Cas9 control cells. Significant SL interactions highlighted in yellow were defined using stringent cut-offs of >0.15 absolute difference and <0.85 relative difference in cell number. (**B**) Western blot analysis of RAD54L expression in parental OVCAR8 cells and two derivative RAD54L knockout (KO) cells following treatment with 1 μM CX-5461 or untreated control for 3 h. Representative of three biological replicates. (**C**) Dose–response proliferation curves for parental OVCAR8 and RAD54L KO cells treated with the indicated concentrations of CX-5461, BMH-21, or olaparib. Cell confluence was monitored over time using IncuCyte live-cell imaging. (**D**) Colony formation assay in parental OVCAR8 and RAD54L KO cells. Cells were treated with CX-5461 (20 nM), doxorubicin (10 nM), or etoposide (200 nM) for 5 days, then cultured for an additional 7 days in drug-free medium prior to fixation and staining with crystal violet. Representative of three independent biological replicates. Colony area (% occupancy per well) was quantified using ImageJ and normalized to the corresponding untreated controls. *n* =  3–4, CX-5461: **P = 0.0013 (RAD54L KO1), ****P < 0.0001 (RAD54L KO2); Doxorubicin: **P = 0.0062 (RAD54L KO1). (**E**) Growth inhibitory concentration (GI50) values for parental OVCAR8 and RAD54L KO cells following treatment with CX-5461, doxorubicin, or etoposide. Cells were treated with increasing drug concentrations for 72 h, then fixed and stained with DAPI for cell number quantification. *n* = 3, **P = 0.0027 (RAD54L KO1), ***P = 0.0001 (RAD54L KO2). Growth inhibition dose–response curves were generated, and GI50 values were calculated using GraphPad Prism 10. Statistical significance was assessed by unpaired *t* test (**D**, **E**). Data are presented as mean ± SEM (**C**–**E**). Source data are available online for this figure.

To uncover synergistic vulnerabilities and gain mechanistic insights into how transcription and replication stress at the rDNA loci intersects with DDR pathways, we performed a synthetic lethal (SL) boutique CRISPR-Cas9 screen targeting 15 DDR genes in combination with Pol I inhibitors CX-5461 (Bywater et al, 2012) and BMH-21 (Peltonen et al, 2014), as well as nine other chemotherapeutic drugs, including topoisomerase (TOP) I and II poisons, DDR inhibitors, and the G-quadruplex (G4) stabilizer pyridostatin (PDS) (Fig. 1A; Appendix Fig. S1A). These 15 DDR genes represent key mediators of HR, NHEJ, microhomology-mediated end joining (MMEJ), and Nucleotide Excision Repair (NER).

Briefly, ovarian cancer OVCAR8-Cas9 cells were transfected with guide RNAs (gRNAs) targeting each DDR gene for 96 h, then re-plated into 96-well plates, and treated with either DMSO or pre-determined GI10, GI25, and GI50 doses of each drug for 72 h (Table Expanded View (EV) 1). Cells were then fixed, stained with DAPI, and imaged to determine cell viability. To identify SL interactions, we calculated both absolute and relative growth differences in gene knock-out (KO) cells relative to Cas9-expressing control cells, applying a stringent threshold of >15% absolute and <85% relative reduction in viability (Appendix Fig. S1A). A summary matrix of SL interactions was generated (Fig. 1A).

As expected, we observed that BRCA1/2 KO confer sensitivity to olaparib and chemotherapeutics. CX-5461 is a dual inhibitor of RNA Pol I and TOP II activities (Bossaert et al, 2021; Bruno et al, 2020; Bywater et al, 2012; Maclachlan et al, 2024), however, by comparison to other TOP II poisons like doxorubicin and etoposide, CX-5461 acts as a DNA structure-driven TOP II poison at G4-enriched, actively transcribed regions, with preferential activity at rDNA loci (Bossaert et al, 2021; Cameron et al, 2024). In this screen, CX-5461 exhibited a distinct sensitivity profile compared to other drugs, showing preferential SL interactions with HR and NHEJ factors and POLQ at low GI10 dose (GI10; 10 nM), with an increasingly prominent HR dependency at higher doses. Among the genes tested, RAD54L emerged as the strongest and most specific SL interactor with both CX-5461 and BMH-21. Notably, RAD54L loss also enhanced OVCAR8 cells’ sensitivity to the PARP inhibitor (PARPi) olaparib and PDS, suggesting a broader role for RAD54L in responding to replication stress and DNA damage at G4-enriched genomic regions, including rDNA.

To validate the SL interaction of RAD54L and inhibitors of Pol I transcription, we generated clonal RAD54L KO OVCAR8 cell lines using CRISPR-Cas9 (Fig. 1B). Two independent clones were obtained: one with low residual RAD54L expression (KO1) and one with complete loss of detectable RAD54L protein (KO2), reflecting distinct CRISPR editing. Proliferation and clonogenic assays confirmed that RAD54L loss significantly sensitized cells to growth inhibition by CX-5461, BMH-21, and olaparib compared to parental OVCAR8 cells (Fig. 1C). Notably, sensitivity to CX-5461 correlated with the extent of RAD54L depletion, with the strongest effect observed in the complete KO2 cell line. In contrast, RAD54L KO cells showed no increased sensitivity to the TOP II poison etoposide and only modest sensitivity to doxorubicin (Fig. 1D,E). These results indicate that the SL interaction between RAD54L loss and CX-5461 is linked to nucleolar-specific stress and rDNA damage rather than a general response to TOP II-mediated DNA damage.

### RAD54L functions as a nucleolar DNA damage response (nDDR) factor

**Figure 2:**
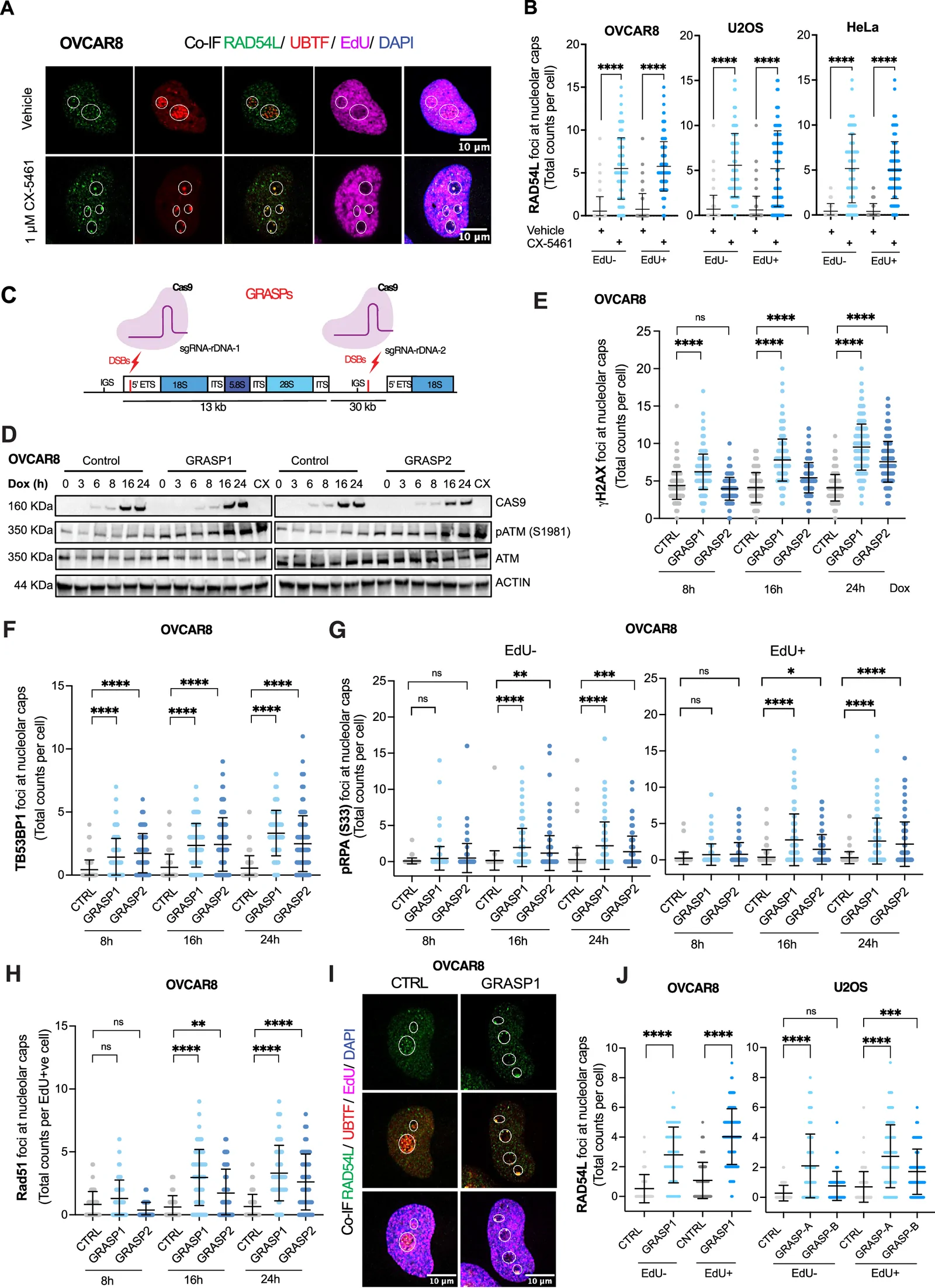
RAD54L is a component of the nucleolar DNA damage response. (**A**) Co-IF analysis of RAD54L and UBTF in OVCAR8 cells treated with 1 µM CX-5461 or vehicle for 3 h. Representative images from two independent experiments. White circles represent nucleolar periphery. (**B**) Quantification of RAD54L foci at nucleolar caps (marked with UBTF) in OVCAR8, U2OS, and HeLa cells treated with CX-5461 as in (A). Quantitation was performed by manual counting of *n* > 100 cells per condition for OVCAR8 and U2OS cells, and *n* > 90 for HeLa cells per condition across two biological replicates. ****P < 0.0001. (**C**) Schematic of the targeted rDNA DSB model depicting an rRNA gene unit and the positions of sgRNA-rDNA target sites. (**D**) Western blot analysis of ATM signaling activity in cells treated with doxycycline (100 ng/ml) for the indicated time points. Actin served as a loading control. Blots are representative of three biological replicates. (**E**) Quantification of γH2AX foci at nucleolar caps (marked with NPM) in OVCAR8 GRASP and control cells upon doxycycline (100 ng/ml) induction for 8, 16, or 24 h. *n* > 130 cells per condition from three biological replicates, ****P < 0.0001; ns, non-significant. Representative images are shown in Appendix Fig. S2D. (**F**) Quantification of TP53BP1 foci at nucleolar caps (marked with UBTF) in cells treated as in (**E**). *n* > 130 cells per condition from two biological replicates, ****P < 0.0001. Representative images are shown in Appendix Fig. S2E. (**G**) Quantification of pRPA (S33) foci at nucleolar caps (marked with NPM) in cells treated as in (**E**). Cells were pulse-labeled with EdU (10 μM) for 30 min to mark replicating cells. *n* > 95 EdU- cells per condition from two biological replicates: ns, non-significant ****P < 0.0001 (GRASP1 16 h), **P = 0.0046 (GRASP2 16 h), ****P < 0.0001 (GRASP1 24 h), ***P = 0.0004 (GRASP2 24 h); *n* > 80 EdU+ cells per condition from two biological replicates ****P < 0.0001 (GRASP1 16 h), *P = 0.0285 (GRASP2 16 h), ****P < 0.0001 (GRASP1 and GRASP2 24 h). Representative images are shown in Appendix Fig. S2F. (**H**) Quantification of RAD51 foci at nucleolar caps (marked with NPM) in cells treated as in (**E**). *n* > 45 EdU+ cells per condition, **P = 0.0019 (GRASP2 16 h), ****P < 0.0001; ns, non-significant. Representative images are shown in Appendix Fig. S2G. (**I**) Co-IF analysis of RAD54L and UBTF in OVCAR8 GRASP and control cells upon doxycycline (100 ng/ml) induction for 24 h. Cells were pulse-labeled with EdU to mark replicating cells. Representative images from two independent replicates. White circles represent nucleolar periphery. (**J**) Quantification of RAD54L foci in nucleolar caps (marked with UBTF) in OVCAR8 and U2OS GRASP and control cells treated as (**I**). OVCAR8: *n* > 55 EdU- cells and *n* > 89 EdU+ cells per condition from two biological replicates, ****P < 0.0001; U2OS: *n* > 50 EdU− cells and *n* > 93 EdU+ cells per condition from two biological replicates: ns, non-significant ****P < 0.0001, ***P = 0.0003. Statistical significance was assessed by one-way ANOVA with Tukey’s multiple comparisons test (**B**, **E**–**G**, **H**, **J**); ns, non-significant; exact *P* values are shown, ****P ≤ 0.0001. Error bars represent mean ± SD (**B**,** E**–**G**, **H**, **J**). Source data are available online for this figure.

RAD54L is a member of the SWI2/SNF2 ATP-dependent DNA translocase family that remodels dsDNA during HR. It regulates RAD51 by stabilizing RAD51-ssDNA filament, promoting strand invasion, and facilitating filament turnover (Mason et al, 2015). Given that CX-5461 induces rDNA-associated DNA damage and exhibits synthetic lethality with HR deficiency (Cameron et al, 2024; Sanij et al, 2020), we assessed whether RAD54L is recruited to the nucleoli following CX-5461 treatment. At baseline conditions, co-immunofluorescence (co-IF) analysis revealed punctate nucleolar RAD54L staining, co-localizing with the nucleolar factor UBTF, which binds active rDNA (Sanij et al, 2008) in OVCAR8, U2OS cells, and HeLa cells (Fig. 2A,B; Appendix Fig. S1B). Upon CX-5461 treatment (1 μM, 3 h), UBTF redistributed to nucleolar caps, indicating inhibition of Pol I transcription and activation of nDDR. RAD54L co-accumulated at nucleolar caps in both EdU-positive and EdU-negative cells, indicating that its redistribution and recruitment are not restricted to S-phase cells (Fig. 2A,B; Appendix Fig. S1B).

To directly interrogate RAD54L function in nDDR, we established inducible, rDNA DSB models using CRISPR-Cas9. We generated clonal OVCAR8 cell lines (#1 and #2) and clonal U2OS cell lines (A and B) stably expressing doxycycline (Dox)-inducible endonuclease Cas9 and two single guide RNAs (sgRNAs) targeting the rDNA (sgRNA-rDNA), named GRASP (Guide RNA for rDNA Specificity) (Fig. 2C). Dox induction for 16–24 h induced Cas9 expression and ATM activation, confirming engagement of an ATM-dependent DDR (Fig. 2D; Appendix Fig. S2A). γH2AX and TP53BP1 foci accumulated at nucleolar periphery by 16 h and persisted at 24 h, indicating sustained DSB signaling (Fig. 2E,F; Appendix Fig. S2B–E).

DSB processing involves end resection, generating ssDNA that is initially coated by replication protein A (RPA) and subsequently replaced by RAD51 to facilitate HR (Korsholm et al, 2020). Consistent with early end resection-associated ssDNA formation, we observed a time-dependent detection of phosphorylated RPA (S33) foci at the nucleoli, as early as 16 h post-induction with Dox in replicating and non-replicating cells (Fig. 2G; Appendix Fig. S2F). RAD51 foci accumulated at nucleolar caps by 16 h and continued to increase by 24 h (Fig. 2H; Appendix Fig. S2G), indicating progressive HR engagement. The induction of rDNA DSBs was also accompanied by suppression of Pol I transcription (Appendix Fig. S2H), consistent with nDDR activation.

RAD54L accumulated at nucleolar caps within 24 h of rDNA DSBs induction in OVCAR8 (Fig. 2I-J) and U2OS cells (Fig. 2J; Appendix Fig. S2I), in both EdU-positive and EdU-negative cells. Nucleolar fractionation followed by western blotting also confirmed increased nucleolar RAD54L levels upon the induction of rDNA DSBs in GRASP1 OVCAR8 cells (Appendix Fig. S2J). Collectively, these data establish RAD54L as a nucleolar DDR factor that is recruited to rDNA damage sites and associated with HR repair at nucleolar caps.

### RAD54L is required for rDNA repair and maintenance of rDNA genomic stability

**Figure 3:**
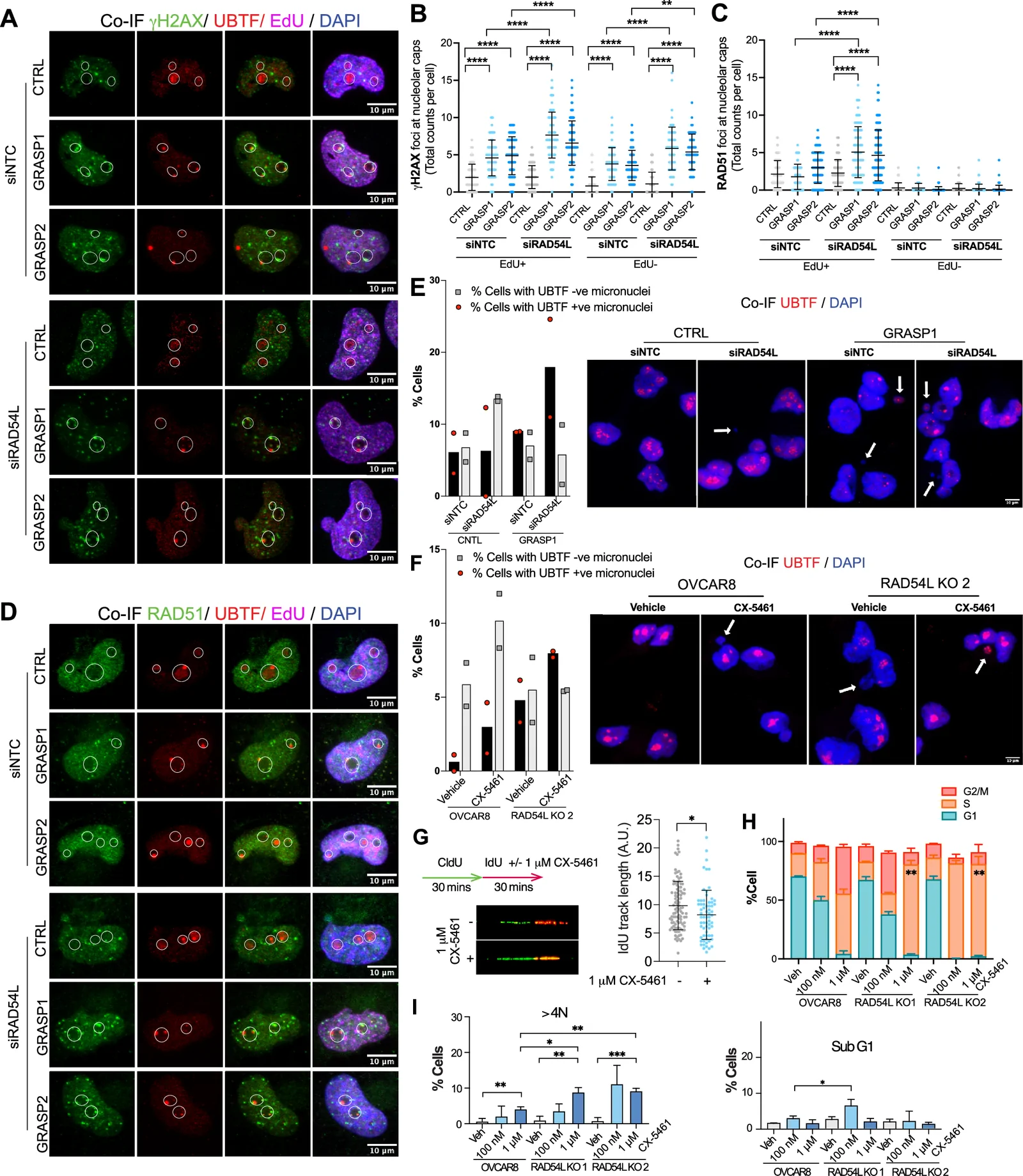
RAD54L is required for nucleolar DNA repair and maintenance of rDNA genomic stability. (**A**) Co-IF analysis of γH2AX and UBTF in OVCAR8 GRASP and control cells transfected with siRAD54L or non-targeting control siRNA, followed by doxycycline induction (100 ng/ml) for 24 h. Cells were pulse-labeled with EdU to mark replicating cells. White circles represent nucleolar periphery. Representative images from two independent replicates. (**B**) Quantification of γH2AX foci at nucleolar caps (marked with UBTF) in cells treated as (**A**). *n* > 62 EdU- cells and *n* > 77 EdU+ cells per condition from two biological replicates, **P = 0.0024; ****P < 0.0001 using one-way ANOVA with Tukey’s multiple comparisons test. (**C**) Quantification of RAD51 foci at nucleolar caps (marked with UBTF) in cells treated as (**A**). *n* > 64 EdU- cells and *n* > 68 EdU+ cells per condition from two biological replicates, ****P < 0.0001 using one-way ANOVA with Tukey’s multiple comparisons test. Representative images are shown in (**D**). (**D**) Co-IF analysis of RAD51 and UBTF in OVCAR8 cells treated as (**A**). White circles represent nucleolar periphery. (**E**) Quantification of micronuclei (indicated by the white arrows) containing rDNA (UBTF-positive) and UBTF-negative micronuclei in OVCAR8 GRASP and control cells transfected with siRAD54L or non-targeting control siRNA. Cells were treated with cytochalasin B (5 μg/ml) for 24 h. Representative images from two independent experiments. The bar graph represents % cells exhibiting micronuclei, over 98 cells per condition were counted from *n* = 2. (**F**) Quantification of micronuclei (indicated by the white arrows) containing rDNA (UBTF-positive) and UBTF-negative micronuclei in OVCAR8 and RAD54L KO cells treated with CX-5461 (100 nM) and cytochalasin B (5 μg/ml) for 24 h. Representative images from two independent experiments. The bar graph represents % cells exhibiting micronuclei, over 135 cells per condition were counted from *n* = *2*. (**G**) DNA fiber assay to assess the effect of CX-5461 on replication fork progression in OVCAR8 cells. Left: schematic of the labeling protocol and representative labeled fibers. Right: the length of IdU tracks per fiber, quantified from *n* ≥ 67 fibers, *P = 0.0175 using Welch’s *t* test. (**H**) Cell cycle analysis by BrdU incorporation and PI staining in parental OVCAR8 and RAD54L KO cells following 72 h treatment with CX-5461 (100 nM or 1 μM). Histogram plots show the percentage of cells in G1, S, and G2M phases. *n* = 3, S phase populations: **P = 0.0044 (OVCAR8 1 μM compared to RAD54L KO1 1 μM), **P = 0.0028 (OVCAR8 1 μM compared to RAD54L KO2 1 μM) using one-way ANOVA with Tukey’s multiple comparisons test. (**I**) Histogram plots of the percentage of cells with >4 N DNA content and sub-G1 fraction, *n* = 3. For >4 N DNA content cell population: **P = 0.0093 (OVCAR8 Veh versus 1 μM), **P = 0.0019 (RAD54L KO1 Veh versus 1 μM), *P = 0.0163 (OVCAR8 1 μM versus RAD54L KO1 1 μM), ***P = 0.0003 (RAD54L KO2 Veh versus 1 μM), **P = 0.0017 (OVCAR8 1 μM versus RAD54L KO2 1 μM) using Welch’s *t* test. For sub-G1 fractions, *P* = 0.0411 (OVCAR8 100 nM versus RAD54L KO1 100 nM) using Welch’s *t* test. Error bars represent mean ± SD (**B**, **C**, **G**) or mean ± SEM (**H**, **I**). Source data are available online for this figure.

We next examined how RAD54L loss impacts the cellular response to rDNA DSBs. siRNA-mediated depletion of RAD54L increased γH2AX and RAD51 foci at the nucleolar periphery following rDNA DSB induction, with these foci co-localizing to nucleolar caps (Fig. 3A–D), indicating defective resolution of rDNA lesions.

RAD54L is a key HR translocase that regulates RAD51 filament dynamics. During HR, RAD51 assembles on resected ssDNA, catalyzes homology search and strand invasion to form a displacement loop (D-loop), and promotes error-free repair. RAD54L promotes RAD51-mediated strand exchange and facilitates RAD51 removal from dsDNA, thereby limiting persistent recombination intermediates (Mason et al, 2015). To assess whether RAD54L loss impacts RAD51 dynamics in a general DNA damage context, we quantified RAD51 foci after ionizing irradiation (IR) (Appendix Fig. S3A,B). IR was used as a complementary system to examine RAD51 kinetics at canonical DSBs across the genome. In control cells, RAD51 foci increased by 4 h, peaked at 8 h, and resolved by 24 h post-IR. In contrast, RAD54L KO cells displayed elevated basal RAD51 foci and sustained RAD51 accumulation from 4 to 24 h, indicating prolonged RAD51-DNA association and defective filament turnover.

Consistent with this, RAD54L knockdown by siRNA increased RAD51 foci at the nucleoli upon rDNA damage (Fig. 3C,D), supporting a role for RAD54L in resolving RAD51-dependent intermediates at rDNA. Functionally, RAD54L-deficient GRASP cells displayed increased micronuclei formation following rDNA DSB induction, including UBTF-positive micronuclei containing rDNA, indicating defective repair and chromosome segregation errors (Fig. 3E). CX-5461 has been reported to induce replication stress at rDNA (Sanij et al, 2020). RAD54L loss similarly increased UBTF-positive micronuclei following CX-5461 treatment, demonstrating enhanced rDNA-associated genome instability under replication stress (Fig. 3F).

The data support a model in which RAD54L contributes to resolving replication-associated stress. CX-5461 impairs replication fork progression (Fig. 3G), suggesting increased replication-associated stress. Consistent with this, CX-5461-treated RAD54L KO cells exhibited increased accumulation in S-phase and increased proportion of cells with >4 N content, indicative of defective mitotic progression and/or failed cytokinesis (Fig. 3H,I). Collectively, these findings suggest a role for RAD54L in limiting replication-associated stress and promoting efficient recovery from rDNA damage, thereby contributing to the maintenance of rDNA genomic integrity.

### Loss of RAD54L attenuates CX-5461-mediated ATM-CHK2 signaling and nucleolar cap formation

**Figure 4:**
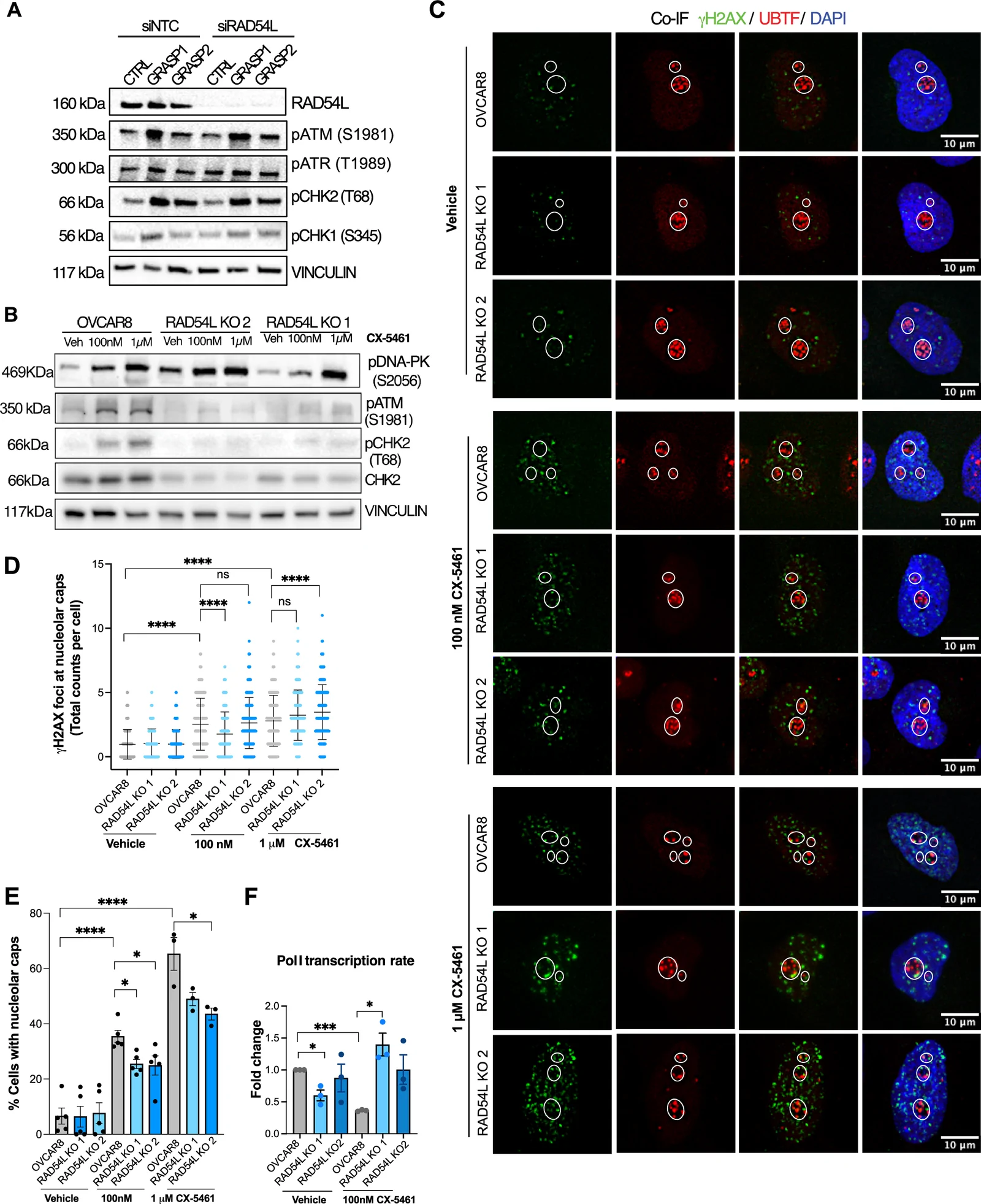
Loss of RAD54L attenuates ATM-CHK2 signaling and impairs nucleolar cap formation upon CX-5461 treatment. (**A**) Western blot analysis of RAD54L expression and ATM and ATR signaling in OVCAR8 control and GRASP cells transfected with siRAD54L or non-targeting control siRNA. Vinculin served as a loading control. (**B**) Western blot analysis of ATM and DNA-PK signaling in parental OVCAR8 and RAD54L KO cells following treatment with CX-5461 for 24 h. Vinculin serves as a loading control. (**C**) Co-IF analysis of γH2AX with UBTF in cells treated as (**B**), white circles represent nucleolar periphery. Representative images from five independent experiments. (**D**) Quantification of γH2AX foci count at nucleolar caps (marked with UBTF) in OVCAR8 and RAD54L KO cells treated as in (**B**), *n* > 170 cells per condition from five biological replicates; ns, non-significant, ****P < 0.0001, ns, non-significant. (**E**) Quantification of the percentage of cells with nucleolar caps in OVCAR8 and RAD54L KO cells treated as in (**B**). Cells with nucleolar caps were defined by UBTF redistribution into peripheral cap structures, with ≤5 UBTF foci per nucleolus, *n* = 3–5, *P = 0.0302 (RAD54L KO1 100 nM) and *P = 0.0231 (RAD54L KO2 100 nM) compared to OVCAR8 100 nM; *P = 0.0124 (RAD54L KO2 1 μM compared to OVCAR8 1 μM); ****P < 0.0001. (**F**) Quantitative real-time PCR analysis of Pol I transcriptional rate using primers targeting the 5’ETS of the 47S pre-rRNA. Expression was normalized to *Vimentin* mRNA and is presented as fold change relative to parental OVCAR8 cells, *n* = 3, *P = 0.042 (RAD54L KO1 Vehicle versus OVCAR8 Vehicle), ***P = 0.0001 (OVCAR8 100 nM versus Vehicle), and *P = 0.0282 (RAD54L KO1 100 nM compared to OVCAR8 100 nM). Statistical significance was assessed by one-way ANOVA with Tukey’s multiple comparisons test (**D**, **E**) and using Welch’s *t* test (**F**). Error bars represent mean ± SD (**D**) or mean ± SEM (**E**, **F**). Source data are available online for this figure.

We next examined whether RAD54L influences DDR signaling in response to rDNA damage. RAD54L loss did not alter phosphorylation of ATM (S1981), pATR (T1989), and DNA-PK (S2056) following IR treatment (Appendix Fig. S4A) or upon the induction of rDNA DSBs in GRASP OVCAR8 cells (Fig. 4A). These data indicate that RAD54L acts downstream of initial DDR activation following the induction of DSBs.

In contrast, RAD54L KO cells displayed reduced ATM-CHK2 activation following CX-5461 treatment, compared to parental cells (Fig. 4B; Appendix Fig. S4B). This phenotype was recapitulated in siRNA-mediated RAD54L depletion, which also showed increased phosphorylation of RPA (S33), indicative of elevated ssDNA accumulation (Appendix Fig. S4C). Notably, DNA-PK phosphorylation at S2056 and ATR phosphorylation at (T1989) were maintained in RAD54L-deficient cells treated with CX-5461, suggesting a shift in DDR pathway utilization towards ATR/DNA-PK signaling (Fig. 4B; Appendix Fig. S4B). These findings support a model in which RAD54L promotes proper processing of CX-5461-induced lesions to enable ATM activation, whereas its loss leads to accumulation of replication-associated ssDNA intermediates and altered DDR pathway engagement.

To further assess RAD54L’s function in resolving CX-5461-mediated rDNA damage, we examined γH2AX levels after 3 h of CX-5461 treatment (Fig. 4C,D). At 1 μM CX-5461, RAD54L KO cells displayed increased γH2AX within and at the nucleolar periphery, indicating increased or persistent DNA damage. In contrast, at 100 nM CX-5461, RAD54L-deficient cells exhibited reduced or comparable nucleolar γH2AX compared to control cells (Fig. 4C,D). At this lower dose, the nucleolar stress response is partial, and γH2AX alone may not fully capture nucleolar reorganization. We therefore assessed this readout in conjunction with UBTF-defined nucleolar cap morphology, which showed that RAD54L loss results in a lower proportion of cells forming nucleolar caps (Fig. 4D). These observations are consistent with reduced ATM-dependent nucleolar reorganization in RAD54L-deficient cells, as ATM activity promotes Pol I transcription repression and subsequent nucleolar reorganization following rDNA damage (Korsholm et al, 2019). Consistent with this, RAD54L loss restored Pol I transcription in CX-5461-treated cells, in association with reduced nucleolar cap formation (Fig. 4E,F).

Collectively, these data indicate that RAD54L is required to convert CX-5461-induced replication stress into ATM-activating DNA lesions that drive nucleolar reorganization. In its absence, ATR and DNA-PK signaling persist, whereas ATM activation, Pol I repression, and nucleolar cap formation are attenuated, leading to defective nDDR and accumulation of persistent rDNA damage.

### RAD54L resolves replication stress at the nucleoli

**Figure 5:**
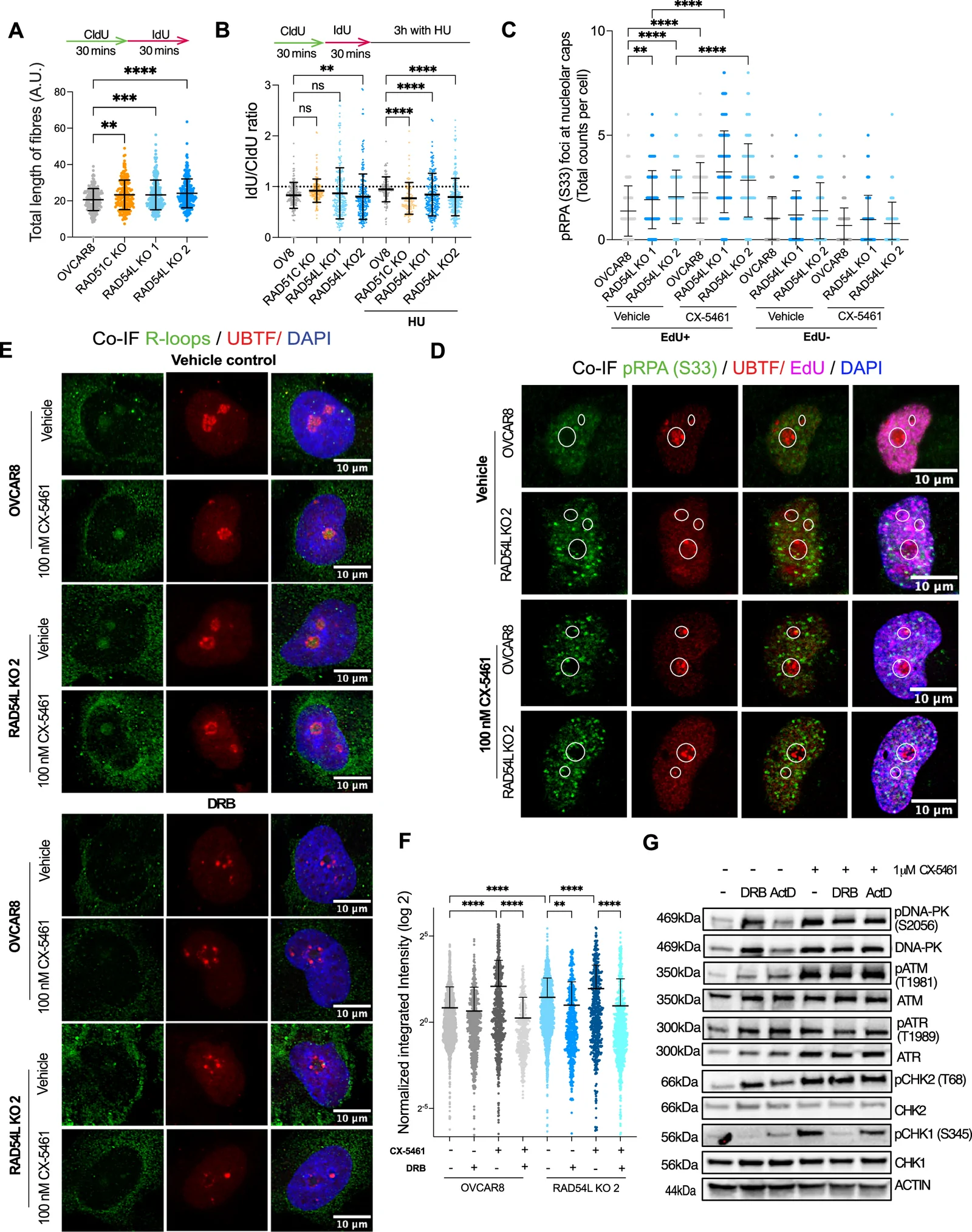
RAD54L resolves replication stress at the nucleoli. (**A**) DNA fiber assay to assess replication fork progression in parental OVCAR8, RAD51C KO, and RAD54L KO cells. Top: schematic of the labeling protocol. Bottom: quantification of total length of CIdU and IdU tracks per fiber from *n* > 180 fibers pooled across three independent experiments. Compared to OVCAR8 cells: **P = 0.0021 (RAD51C KO), ***P = 0.0009 (RAD54L KO1), ****P < 0.0001 (RAD54L KO2) using one-way ANOVA with Tukey’s multiple comparisons. (**B**) DNA fiber assay to assess fork protection and stability in OVCAR8 and RAD54L KO cells. Cells were labeled and either harvested or treated with hydroxyurea (HU) (2 mM) for 3 h. Top: schematic of the labeling protocol. Bottom: quantification of the ratio of the length of the IdU track to the CIdU track per fiber from *n* > 100 fibers pooled across three independent experiments. **P = 0.0093; ****P < 0.0001; ns, non-significant using Kruskal–Wallis test-multiple comparisons. (**C**) Co-IF analysis of pRPA (S33) with UBTF. Parental OVCAR8 and RAD54L KO cells were treated with CX-5461(100 nM) for 3 h. Cells were pulse-labeled with EdU to mark replicating cells. Quantification of pRPA (S33) foci in nucleolar caps (marked with UBTF). *n* > 150 cells per baseline condition across four independent experiments and *n* > 80 from CX-5461- treated conditions across two independent experiments **P = 0.0013; ****P < 0.0001 using one-way ANOVA with Tukey’s multiple comparisons. (**D**) Representative images from two independent experiments as described in (**C**), white circles represent nucleolar periphery. (**E**) Co-IF analysis of R-loop with UBTF. Parental OVCAR8 and RAD54L KO2 cells were pre-treated with DRB (100 μM) for 1 h, followed by CX-5461(100 nM) for 3 h. Representative images from two independent experiments. (**F**) Quantification of R-loop signal intensity in cells treated as (**E**). R-loop signal intensity was quantified using CellProfiler. Integrated intensity values were normalized to the corresponding median value of parental OVCAR8 cells within each experiment, *n* > 110 cells per condition from two independent experiments, **P = 0.0036; ****P < 0.0001 using one-way ANOVA with Tukey’s multiple comparisons test. (**G**) Western blot analysis of ATM, ATR, and DNA-PK signaling in OVCAR8 cells. Cells were treated with DRB (100 μM) or ActD (10 nM) for 1 h, followed by CX-5461 (1 μM) for 6 h. Actin serves as a loading control. Blots are representative of two biological replicates. Error bars represent mean ± SD (**A**–**C**, **F**). Source data are available online for this figure.

Beyond its canonical function in HR, RAD54L has been implicated in replication fork remodeling, where it restrains fork progression and promotes fork reversal to maintain fork stability under replication stress (Uhrig et al, 2024). We therefore assessed the impact of RAD54L loss on fork dynamic during global replication. RAD54L KO cells exhibited increased replication fork speed, indicating impaired fork restraint, with KO2 cells (complete RAD54L KO) displaying reduced fork progression continuity (CldU/IdU tract ratio), suggesting altered fork stability (Fig. 5A,B). Notably, RAD51C loss similarly resulted in increased fork speed; however, fork progression continuity was largely maintained under basal conditions and was reduced following hydroxyurea (HU) treatment, indicating impaired fork protection under replication stress (Hill et al, 2018) (Fig. 5B). Together, these findings are consistent with distinct, context-dependent contributions of RAD54L and RAD51C to replication dynamics, with RAD54L influencing fork progression and stability under basal conditions, and RAD51C contributing to fork protection under stress conditions.

RAD54L KO cells exhibit a high baseline of pRPA S33 at nucleoli in S-phase cells, consistent with replication-associated ssDNA accumulation, which was further enhanced following CX-5461 treatment (Fig. 5C,D). CX-5461 induces replication stress associated with G4 stabilization and/or TOP II trapping at rDNA loci (Bossaert et al, 2021; Cameron et al, 2024; Xu et al, 2017). Loss of RAD54L exacerbated CX-5461-induced replication stress and DNA end resection, as evidenced by increased ssDNA gaps at rDNA loci (Fig. 5C,D).

Given that replication stress and ssDNA are frequently associated with increased RNA-DNA hybrid (R-loop) formation, we next assessed nucleolar R-loops. RAD54L KO cells displayed elevated basal nucleolar R-loops, consistent with increased replication stress (Fig. 5E,F; Appendix Fig. S5A). CX-5461 treatment increased nucleolar R-loops in parental cells; however, this was not further enhanced in RAD54L KO cells, suggesting that RAD54L loss and CX-5461 converge on a shared mechanism driving R-loop accumulation.

Recent studies have shown that nucleolar Pol II generates antisense intergenic ncRNAs (asincRNAs) within the rDNA IGS, which can form R-loops that regulate nucleolar organization (Abraham et al, 2020). These transcripts form an R-loop “shield” that restricts the synthesis of Pol I-dependent sense intergenic ncRNAs (sincRNAs), a process that, when deregulated, can disrupt nucleolar condensates' integrity and compromise nucleolar organization (Abraham et al, 2020). We therefore tested whether Pol II activity contributes to nucleolar R-loop formation under conditions of CX-5461 treatment and RAD54L loss. Pre-treatment with the Pol II elongation inhibitor DRB (5,6-dichlorobenzimidazole 1-β-D-ribofuranoside) for 1 h significantly reduced nucleolar R-loops in both parental and RAD54L KO cells, following 3-h vehicle or CX-5461 treatment, indicating that Pol II activity is a major driver of nucleolar R-loop accumulation (Fig. 5E,F).

Notably, DRB treatment attenuated CX-5461-induced phosphorylation of DNA-PK, ATR, and CHK1, whereas ATM-CHK2 signaling remained largely unaffected (Fig. 5G). In contrast, inhibition of Pol I transcription with actinomycin D (ActD) had minimal impact on CX-5461-induced DDR signaling. These data indicate that Pol II-dependent R-loops act upstream of DNA-PK- and ATR-mediated signaling but are not required for CX-5461-mediated ATM activation. Furthermore, DRB treatment alone disrupted nucleolar organization, highlighting a critical role for Pol II transcription in maintaining nucleolar structure (Fig. 5E). Collectively, these findings demonstrate that RAD54L preserves replication fork stability at rDNA, limiting ssDNA accumulation and constraining R-loop formation, while Pol II-driven R-loops contribute to CX-5461-mediated replication stress and selectively activate DNA-PK/ATR signaling pathways.

### Increased Pol II transcriptional activity upon rDNA damage is associated with rDNA repair

**Figure 6:**
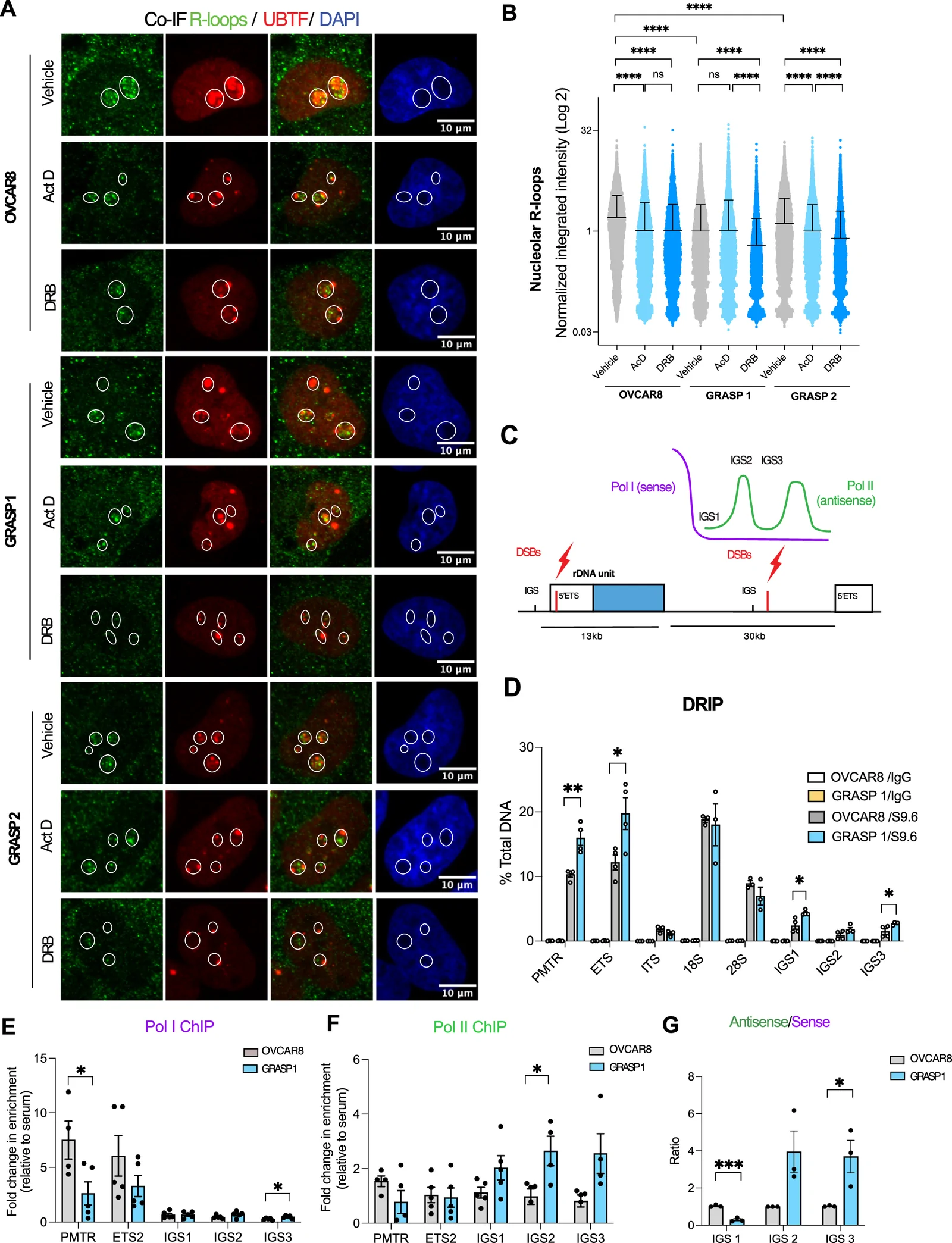
Increased Pol II transcriptional activity at rDNA intergenic regions upon rDNA damage. (**A**) Co-IF analysis of R-loop with UBTF. GRASP and OVCAR8 control cells treated with doxycycline (100 ng/ml) for 24 h. Actinomycin D (10 nM) or DRB (100 μM) was added 4 h before harvest. White circles represent nucleolar periphery. Representative images from three independent experiments. (**B**) Quantification of R-loop signal intensity in the experiments described in (**A**). R-loop signal intensity was quantified using CellProfiler. Integrated intensity values were normalized to the corresponding median value of OVCAR8 control cells within each experiment, *n* > 180 cells per condition from three independent experiments, ****P < 0.0001; ns, non-significant using one-way ANOVA with Tukey’s multiple comparisons test. Error bars represent mean ± SD. (**C**) A schematic of Pol II transcription at rDNA intergenic spacers (IGSs) producing antisense intergenic ncRNAs upon DSBs at rDNA. (**D**) DRIP–qPCR analysis of rDNA-associated R-loops in parental OVCAR8 and GRASP1 cells. Genomic DNA was subjected to immunoprecipitation using the S9.6 antibody or an IgG control, followed by qPCR with primer sets spanning the rDNA repeat and IGS regions. *n* = 4–5, **P = 0.0035 (PMTR), *P = 0.0326 (ETS), *P = 0.0142 (IGS1), *P = 0.0315 (IGS3). (**E**) ChIP-qPCR analysis of Pol I binding to rDNA in OVCAR8 and GRASP1 cells. *n* = 4–5, *P = 0.04 (PMTR), *P = 0.0249 (IGS3). (**F**) ChIP-qPCR analysis of Pol II binding to rDNA in OVCAR8 and GRASP1 cells. *n* = 4–5, *P = 0.022 (IGS2). (**G**) Strand-specific RT–qPCR (ss-RT) analysis of sense and antisense intergenic ncRNAs transcribed from rDNA IGS regions. The antisense-to-sense intergenic ncRNAs ratio was calculated for each site, and the relative ratio was calculated by normalizing to the ratio of GRASP cells to control cells within each experiment. *n* = 3, ***P = 0.0005 (IGS1), *P = 0.0385 (IGS3). Statistical significance was assessed by unpaired *t* test (**D**–**G**). Error bars represent mean ± SEM (**D**–**G**). Source data are available online for this figure.

We next investigated whether Pol II activity and Pol II-driven nucleolar-R-loops contribute to nDDR following rDNA DSBs, particularly as Pol I transcription is suppressed upon rDNA DSB induction (Appendix Fig. S2H). Co-IF for R-loops and UBTF revealed a reduction in overall nucleolar R-loop signal following rDNA DSB induction (Fig. 6A,B). In OVCAR8 control cells, both the Pol I inhibitor ActD and the Pol II inhibitor DRB significantly reduced R-loop levels. However, Pol II inhibition resulted in a more pronounced reduction in R-loops in GRASP cells compared to Pol I inhibition, indicating that R-loops generated upon rDNA DSBs are predominantly produced by Pol II.

To define the genomic distribution of these R-loops, we performed DRIP-PCR. R-loop signals were enriched at the rDNA promoter, 5’ external transcribed spacer (ETS), and IGS regions proximal to DSB sites, with no increase detected within the ITS, 18S, or 28S regions (Fig. 6C,D; Appendix Fig. S5B). These findings indicate that damage-induced R-loops localize to regulatory and intergenic regions rather than coding rDNA sequences. We next assessed Pol I and Pol II occupancy across rDNA by chromatin immunoprecipitation (ChIP). Under basal conditions, Pol I was enriched at the promoter and 5’ETS of the 47S rRNA gene, whereas active Pol II (pS2) localized predominantly to IGS regions (Fig. 6E,F; Appendix Fig. S5C). Following rDNA DSB induction, Pol II occupancy increased at IGS regions in both OVCAR8 and U2OS GRASP models, correlating with an increased antisense intergenic (asincRNA) to sense intergenic (sincRNA) transcript ratio (Fig. 6G; Appendix Fig. S5D). In contrast, Pol I occupancy at the promoter was reduced, consistent with decreased 47S pre-rRNA transcription (Fig. 6E; Appendix Fig. S2H). Thus, rDNA damage induces a transcriptional switch from Pol I- to Pol II-driven activity within rDNA loci.

**Figure 7:**
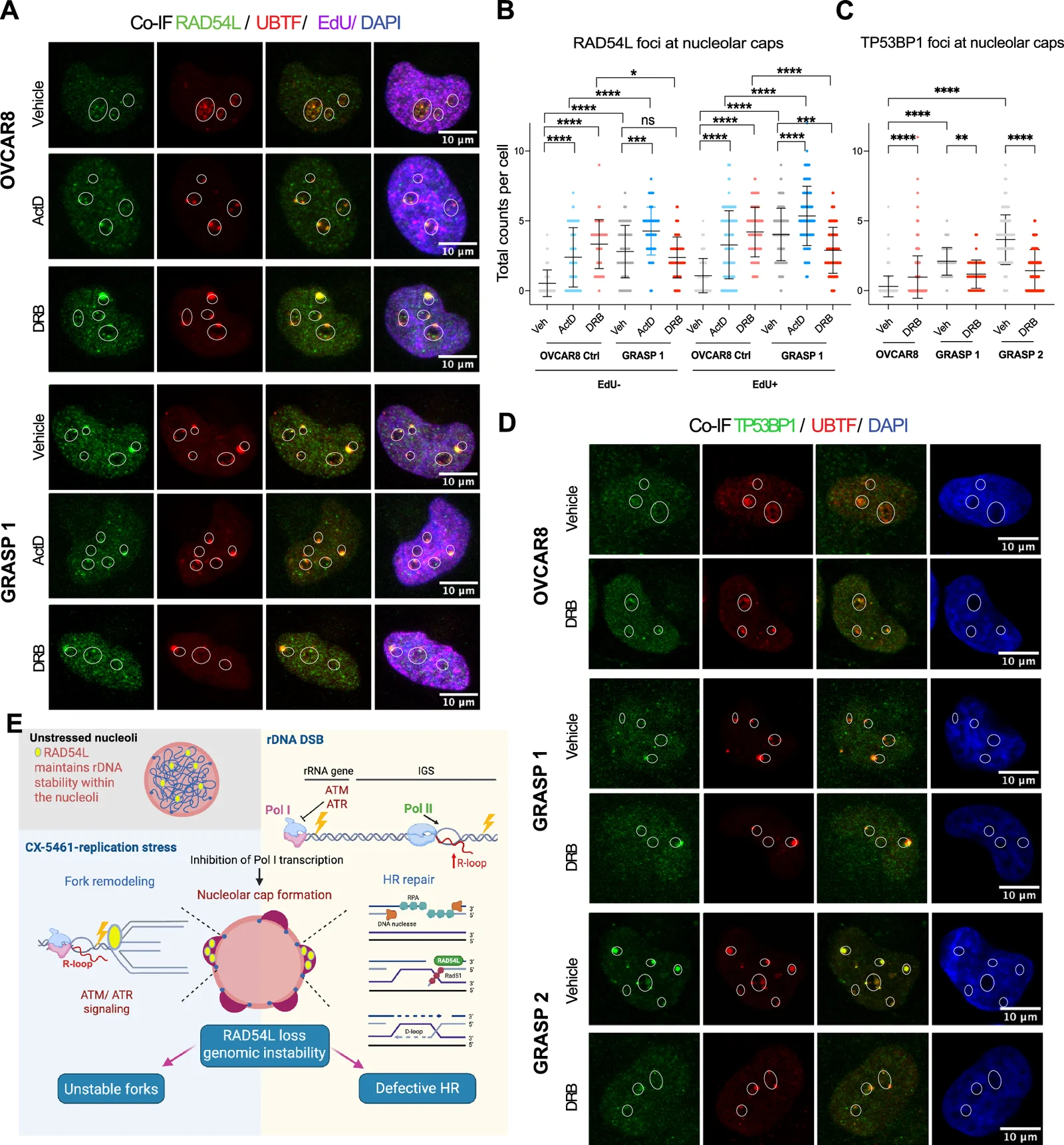
Pol II transcription activity is linked to nucleolar reorganization and the recruitment of DNA repair factors to nucleolar caps upon the induction of rDNA DSBs. (**A**) Co-IF analysis of RAD54L with UBTF in parental OVCAR8 and GRASP1 cells. Cells were treated with doxycycline (100 ng/ml) for 24 h. Actinomycin D (10 nM) or DRB (100 μM) was added 4 h before the endpoint. White circles represent nucleolar periphery. Representative images from two independent experiments. (**B**) Quantification of RAD54L foci at nucleolar caps (marked with UBTF) in parental OVCAR8 and GRASP1 cells treated as (**A**). *n* > 50 EdU- cells per condition, *P = 0.0425, ***P = 0.0002, ****P < 0.0001, ns, non-significant. *n* > 88 EdU+ cells per condition, ***P = 0.001, ****P < 0.0001 from two independent experiments. (**C**, **D**) Co-IF analysis and quantification of TP53BP1 foci at nucleolar caps (marked with UBTF) in parental OVCAR8 and GRASP cells treated with doxycycline (100 ng/ml) for 24 h. DRB (100 μM) was added 4 h before the endpoint. Representative images from three independent experiments. White circles represent nucleolar periphery. *n* > 50 for GRASP 1 and corresponding control cells; and *n* > 150 for GRASP 2 and corresponding control cells from three independent experiments, **P = 0.002, ****P < 0.0001. Statistical significance was assessed by one-way ANOVA with Tukey’s multiple comparisons test (**B**, **C**). Data are presented as mean ± SD (**B**, **C**). (**E**) Schematic model illustrating nucleolar DDR activation upon rDNA damage. RAD54L resolves replication stress within the nucleolus at baseline conditions and is required for resolving CX-5461-mediated replication stress. RAD54L is also recruited to nucleolar caps following rDNA double-strand breaks, where it promotes HR repair. rDNA damage is also associated with increased RNA polymerase II (Pol II) transcription from intergenic spacer (IGS) regions, generating R-loops that are linked to nucleolar reorganization and subsequent recruitment of RAD54L and TP53BP1 to nucleolar caps. Created in BioRender. Sanij, E (2026) https://BioRender.com/66yk65z. Source data are available online for this figure.

Given the observed defects in nucleolar organization in DRB-treated cells (Figs. 5E and  6A), we assessed the impact of DRB treatment on nucleolar fragmentation and cap formation and the localization of RAD54L and TP53BP1 to nucleolar caps (Fig. 7A–D; Appendix Fig. S6A,B). As expected, ActD, DRB, or rDNA DSB induction alone triggered nucleolar stress and cap formation. However, only Pol II inhibition significantly impaired nucleolar cap formation following rDNA damage, independent of cell cycle stage (Fig. 7A,B; Appendix Fig. S6A,B).

RNA transcribed by Pol II at DSB sites has been implicated in activation of DDR and recruitment of DNA repair factors, including TP53BP1 (Ketley et al, 2022; Petermann et al, 2022). Previous studies have shown that damage-induced transcripts can facilitate dissociation of the TIRR-TP53BP1 complex, thereby promoting TP53BP1 recruitment to sites of DSBs (Ketley et al, 2022). Consistent with this, DRB-mediated Pol II inhibition significantly reduced recruitment of RAD54L and TP53BP1 to nucleolar caps, whereas ActD had no such effect (Fig. 7A,B; Appendix Fig. S6A,B), despite unchanged protein levels (Appendix Fig. S6C).

Together, these data demonstrate that Pol II-dependent transcription and R-loop formation in response to DSBs at rDNA are essential for nucleolar reorganization and selective activation of DNA-PK/ATR signaling pathways. This transcriptional response promotes nucleolar cap formation and facilitates recruitment of repair factors, including RAD54L, thereby enabling efficient rDNA repair. These findings support a model in which Pol II activity upon rDNA damage establishes a transcription-coupled environment that is permissive for nDDR factor assembly.

## Discussion

The rDNA repeats represent one of the most structurally complex and fragile genomic regions in mammalian cells. Their repetitive organization, high Pol I transcriptional output, and chromatin architecture create susceptibility to TRCs and genome instability. Here, we define a nucleolar surveillance mechanism that coordinates transcription, replication stress, and DNA repair through the DNA translocase RAD54L (Fig. 7E).

Using complementary models of nucleolar stress, targeted rDNA DSBs, and CX-5461-mediated replication stress, we show that RAD54L is recruited to nucleolar caps and is required to maintain rDNA integrity. Loss of RAD54L under both rDNA DSBs and CX-5461 conditions led to increased rDNA lesions and elevated micronuclei formation, consistent with heightened genomic instability. Importantly, RAD54L-deficient cells exhibited prolonged RAD51 filament persistence following rDNA DSBs, supporting a role for RAD54L in promoting RAD51 filament turnover and efficient HR progression. In addition to these damage-associated roles, RAD54L loss resulted in increased ssDNA accumulation and elevated nucleolar R-loops under basal conditions, accompanied by altered replication fork dynamics, indicating a role in replication fork restraint and stability. These findings support a dual function for RAD54L within the nucleolus. Under unstressed conditions, RAD54L supports replication fork restraint and rDNA integrity, thereby maintaining optimal Pol I transcription. Upon nucleolar stress or rDNA damage, RAD54L is further enriched at nucleolar caps, where it contributes to the execution of the nDDR.

rDNA DSBs activate an ATM-dependent repair program involving relocalization of damaged repeats to nucleolar caps (Korsholm et al, 2020; van Sluis and McStay, 2017). HR at rDNA loci must be tightly regulated to prevent deleterious recombination within highly repetitive rDNA arrays (Gal et al, 2024). The nDDR model established by the McStay laboratory (van Sluis and McStay, 2015), identified that RAD51 recruitment to nucleolar caps can occur outside S phase; however, in our system RAD51 foci at nucleolar caps are enriched in S-phase cells (Fig. 3C), indicating that canonical RAD51-dependent HR is preferentially engaged in replicating cells while remaining context-dependent in G1. Our data place RAD54L downstream of DSB sensing, facilitating HR-mediated repair following end resection and spatial repositioning, as RAD54L loss did not impair ATM activation in GRASP cells but disrupted downstream repair processes.

Beyond HR, RAD54L plays a critical role in replication fork regulation. RAD54L KO cells exhibited impaired fork restraint and instability. In contrast, RAD51C loss increased fork speed but primarily affected fork stability under HU-induced stress, consistent with a role in fork protection. These findings suggest context-dependent contributions of RAD54L to replication-associated processes, including fork stability under basal conditions and during nucleolar stress. Rad54L loss exacerbated CX-5461-induced replication stress, suggesting that RAD54L contributes to replication stress tolerance, with prominent effects observed at nucleolar/rDNA regions.

Mechanistically, RAD54L deficiency led to increased ssDNA accumulation and altered DDR pathway engagement in response to CX-5461. Loss of RAD54L attenuated ATM-CHK2 signaling while maintaining ATR/DNA-PK activation, consistent with a shift from canonical ATM-dependent rDNA DSB repair toward replication stress-associated signaling. These findings support a model in which RAD54L facilitates processing of replication-associated lesions into ATM-activating structures, whereas its absence leads to accumulation of ssDNA intermediates that preferentially engage ATR and DNA-PK pathways. This imbalance was associated with defective nucleolar cap formation and incomplete repression of Pol I transcription, indicating failure to establish a coordinated nDDR.

Another key finding of this study is the role of Pol II in nDDR. rDNA damage-induced Pol II recruitment to IGS regions, increasing antisense transcription and R-loop formation. Pol II-derived R-loops acted upstream of DNA-PK and ATR signaling but were dispensable for ATM activation. Inhibition of Pol II transcription impaired nucleolar cap formation and reduced recruitment of RAD54L and TP53BP1, demonstrating that Pol II activity contributes to nDDR. Together, these findings support a model in which Pol II-dependent transcription upon rDNA DSBs establishes an environment that promotes nucleolar compartmentalization and repair factor recruitment.

Integrating these observations, we propose a model in which RAD54L and Pol II coordinate interconnected but temporally distinct aspects of rDNA maintenance. Under basal conditions, RAD54L acts at highly transcribed rDNA loci to restrain fork progression and stabilize replication forks, thereby limiting ssDNA accumulation, transcription-replication conflicts, and pathological R-loop formation. Under nucleolar stress, Pol II activity increases at intergenic rDNA regions, and Pol II-driven R-loops become the predominant species, where they are associated with nucleolar reorganization and the formation of a repair-permissive nucleolar DDR compartment. In this context, RAD54L functions both to constrain replication stress and to promote HR-mediated repair downstream of end resection, ensuring efficient processing of replication-associated lesions. In its absence, lesions persist as ssDNA- and R-loop-rich intermediates, leading to misdirected DDR signaling and defective rDNA repair.

Finally, these findings have therapeutic implications. Oncogene-driven cancer cells exhibit elevated ribosome biogenesis and dependency on nucleolar function (Bywater et al, 2012; Kang et al, 2021). Our data demonstrate that RAD54L enables tolerance to nucleolar replication stress and that its loss sensitizes cells to Pol I inhibition and PARP inhibition. These results suggest that RAD54L status, or broader alterations in nucleolar HR and replication stress pathways, may serve as biomarkers of response to nucleolar-targeting therapies. Given ongoing clinical evaluation of CX-5461 in solid cancers (NCT04890613, NCT06606990, NCT07147231), our findings highlight nDDR pathways as actionable vulnerabilities in cancers with elevated rDNA stress.

## Methods

**Table:** Reagents and tools table

Reagent/resource	Reference or source	Identifier or catalog number
**Experimental models**		
OVCAR-8 human ovarian cancer cell	National Cancer Institute	All cell lines were verified using STR profiling
OVCAR-4 human ovarian cancer cell	National Cancer Institute	
Cas9-inducible U2OS cell	Munoz et al, 2014	
RAD51C knock-out cell	Kondrashova et al, 2018	
HeLa cervical cancer cell line	ATCC	
**Recombinant DNA**		
pCW-Cas9-Blast	gift from Mohan Babu	Addgene #83481
Cas9-mCherry lentivirus	Victorian Centre for Functional Genomics	Addgene #70182
pCLIP-dual-SFFV-Puromycin vector	Transonic	
**Antibodies**		
Rabbit-pATM (S1981)	ABClonal	#AP1030
Mouse-ATM	GeneTex	#GTX70103
Mouse-pATR (T1989)	GeneTex	#GTX128145
Rabbit-ATR	GeneTex	#GTX128146
Rabbit-pCHK2 (T68)	Cell Signalling	#2197S
Rabbit-CHK2	Cell Signalling	#2662S
Rabbit-pCHK1 (S345)	Cell Signalling	#2348S
Mouse-CHK1	Santa Cruz	#SC-8408
Rabbit-pRPA(S33)	Novus Biologicals	#NB100544
Rabbit-pRPA(S4/8)	Thermo Fisher	#A300-245A
Rat-RPA32	Cell Signalling	#2208 T
Mouse-pHistone H2A.X (S139)	Sigma-Aldrich	#05-636-I
Mouse-pHistone H2A.X (S139)	Abcam	#ab81299
Rabbit-Cas9	Cell Signalling	#65832
Rabbit-CIAPIN1	ABClonal	#A6336
Rabbit-Fibrillarin	Abcam	#AB5821
Mouse-Nucleophosmin	Abcam	#AB10530
Muose-TP53BP1	Sigma-Aldrich	#05-726
Mouse-DNA–RNA Hybrid (R- loop)	Provided by Andrew Deans	
Rabbit-DNA–RNA Hybrid (R- loop)	Sigma-Aldrich	#MABE1095
Mouse-PCNA	Abcam	#AB18197
Mouse-UBTF	Santa Cruz	#SC-13125
Rabbit-UBTF	In-house made	
Mouse-Actin	Thermo Fisher	#MA511869
Mouse-Vinculin	Santa Cruz	#SC-73614
Rabbit-beta-tubulin	Abcam	#AB6046
Rabbit-RAD51	Abcam	#AB63801
Mouse-RAD54L	Santa Cruz	#SC-374598
Rabbit-Histone 3	Abcam	#AB1791
Rat-BrdU	Abcam	#AB6326
Mouse-BrdU	Becton Dickinson	#347580
Rabbit-Phospho-DNA-PKcs (S2056)	Cell Signalling	#68716
Rabbit-DNA-PKcs	Cell Signalling	#38168
Rabbit-POLR1A	Cell Signalling	#24799
Rabbit-RNA polymerase II CTD repeat YSPTSPS (S2)	Abcam	#AB5095
Normal Rabbit IgG	Cell Signalling	#2729
FITC-conjugated anti-mouse IgG	Jackson ImmunoResearch	515-095-003
Goat anti-mouse HRP-conjugated	Bio-Rad	#1721011
Goat anti-rat HRP-conjugated	Bio-Rad	#5204-2504
Goat anti-rabbit HRP-conjugated	Bio-Rad	#1706515
Alexa Fluor™ Goat anti-Rabbit 488	Thermo Fisher	#A11008
Alexa Fluor™ Goat anti-Mouse 488	Thermo Fisher	#A11001
Alexa Fluor™ Goat anti-Rabbit 594	Thermo Fisher	#A11012
Alexa Fluor™ Donkey anti-Mouse 594	Thermo Fisher	#A21203
Alexa Fluor™ Goat anti-Rabbit 647	Thermo Fisher	#A32733
Alexa Fluor™ Chicken anti-rat antibody 488	Invitrogen	#A21470
**Oligonucleotides and other sequence-based reagents**		
rDNA sgRNA1	Horizon	Table EV2
rDNA sgRNA2	Horizon	Table EV2
RAD54L crRNAs	Horizon	CR-004592
BRCA1 crRNAs	Horizon	CR-003461
BRCA2 crRNAs	Horizon	CR-003462
ATM crRNAs	Horizon	CR-003201
ATR crRNAs	Horizon	CR-003202
TP53BP1 crRNAs	Horizon	CR-003548
RAD51C crRNAs	Horizon	CR-010534
RAD51 AP1 crRNAs	Horizon	CR-017166
BARD1 crRNAs	Horizon	CR-003873
ERCC1crRNAs	Horizon	CR-006311
XPC crRNAs	Horizon	CR-016040
XRCC3 crRNAs	Horizon	CR-012067
XRCC6 crRNAs	Horizon	CR-005084
XRCC5 crRNAs	Horizon	CR-010491
POLQ crRNAs	Horizon	CR-015180
PLK1 crRNAs	Horizon	CR-003290
tracrRNA	Horizon	U-002000
ON-TARGETplus non-targeting Pool	Horizon	D-001810-10
RAD54L siRNA	Horizon	Table EV3
PCR primers	This study	Table EV4
**Chemicals, enzymes, and other reagents**		
CX-5461	MedChemExpress	HY -13323
VE-821	Selleckchem	S8007
BMH-21	Selleckchem	S7718
Prexasertib	Selleckchem	S7178
Pyridostatin	Selleckchem	S7444
Olaparib	Selleckchem	S1060
Etoposide	Selleckchem	S1225
Topotecan	Selleckchem	S9321
Cisplatin	Hospital pharmacy	
Oxaliplatin	Hospital pharmacy	
Doxorubicin	Hospital pharmacy	
Actinomycin D	Sigma-Aldrich	A1410
5,6-Dichlorobenzimidazole 1-β-D-ribofuranoside	Sigma-Aldrich	D1916
Cytochalasin B	Sigma-Aldrich	C6762
Doxycycline	Sigma-Aldrich	D9891
Lipofectamine RNAiMAX	Thermo-Fisher	13778075
RPMI-1640	Gibco	11530586
DMEM	Gibco	11-965-092
HEPES	Gibco	11560496
GlutaMAX	Gibco	35050061
Fetal bovine serum	HyClone	SH3007102
5-bromo-2-deoxyuridine (BrdU)	Sigma-Aldrich	B5002
Propidium iodide	Sigma-Aldrich	P4170
SuperScript IV reverse transcriptase	Thermo Fisher Scientific	18080044
Fast SYBR Green Master Mix	Applied Biosystems	4385610
5-chloro-2-deoxyuridine (CIdU)	Sigma-Aldrich	C6891
5-iodo-2-deoxyuridine (IdU)	Sigma-Aldrich	I7125
β-agarase	New England Biolabs	M0392S
Micrococcal nuclease	New England Biolabs	M0247
Proteinase K	Roche	3115828001
RNase H	New England Biolabs	M0297L
DharmaFECT 4	Horizon	31985070
Protein A/G magnetic beads	Thermo Fisher Scientific	88803
**Software**		
GraphPad Prism 10	https://www.graphpad.com/	
ImageJ	https://imagej.net/ij/	
CellProfiler	https://cellprofiler.org	
FlowJo v10	https://www.flowjo.com/	
Affinity Design 2.0	https://www.affinity.studio	
**Other**		
IncuCyte S3 ZOOM live-cell imaging system	Sartorius	
LSRFortessa flow cytometer	BD Biosciences	
STELLARIS 5 confocal microscope	Leica	
FiberComb combing instrument	Genomic Vision	
THUNDER microscope	Leica	
Cellomics CX7 LZR high-content platform	Thermo Fisher Scientific	
BioTek liquid handle	Agilent	

### Methods and protocols

#### Cell lines and culture conditions

Human OVCAR8 cells were transduced with an inducible Cas9 lentiviral construct (pCW-Cas9-Blast; a gift from Mohan Babu; Addgene #83481) and selected with blasticidin (10 µg/mL) for 7 days prior to single-cell sorting to derive clonal populations. The inducible Cas9-U2OS cell line was generated by the Lachaud laboratory as described previously (Munoz et al, 2014).

To generate inducible, locus-specific rDNA double-strand break (DSB) cell lines, OVCAR8-Cas9 and U2OS-Cas9 cells were transduced with lentiviral particles encoding a dual-sgRNA cassette in the pCLIP-dual-SFFV-Puromycin vector (sgRNA1 and sgRNA2; Table EV2). Single-cell clones were isolated and expanded. These clonal lines are referred to as “GRASP”. Cells transfected with the empty pCLIP vector were used as controls.

For constitutive Cas9 expression in OVCAR8 cells, cells were transduced with Cas9-mCherry lentivirus (FUCas9Cherry; a gift from Marco Herold; Addgene #70182) obtained through the Victorian Centre for Functional Genomics (VCFG), Melbourne. Cas9-positive cells were enriched by fluorescence-activated cell sorting (FACS) by collecting the top 10% mCherry-expressing population and expanding the sorted cells.

RAD51C KO cell line was described previously in (Kondrashova et al, 2018). RAD54L knockout clonal lines were generated in OVCAR8-Cas9 cells by reverse transfection with synthetic crRNAs targeting RAD54L (Reagents and tools Table) complexed with tracrRNA (1:1 molar ratio) using DharmaFECT4 (Horizon), followed by single-cell sorting and clonal expansion. RAD54L loss was confirmed by immunoblotting, and two independent RAD54L-deficient clones were selected for downstream experiments.

OVCAR8 and derivative cell lines were cultured in RPMI-1640 supplemented with 20 mM HEPES, 10% (v/v) fetal bovine serum (FBS), and 2 mM GlutaMAX (Gibco, 35050061). U2OS and HeLa cells were cultured in DMEM supplemented with 20 mM HEPES, 10% (v/v) FBS, and 2 mM GlutaMAX. Cells were maintained at 37 °C in a humidified incubator with 5% CO₂ and were cultured for a maximum of 8–10 weeks. All cell lines were authenticated by short tandem repeat (STR) profiling and routinely tested for mycoplasma contamination by PCR.

#### Compounds

CX-5461 was purchased from MedChemExpress (HY-13323) and prepared as a10 mM stock in 50 mM NaH_2_PO_4_. VE-821 (Selleckchem, S8007), BMH-21(Selleckchem, S7718), prexasertib (Selleckchem, S7178), pyridostatin (Selleckchem, S7444), and olaparib (Selleckchem, S1060) were prepared as 10 mM stock solutions in DMSO. Etoposide (Selleckchem, S1225) was prepared as a 100 mM stock in DMSO. Topotecan (Selleckchem, S9321) was prepared as 1 mM stock in 0.9% (w/v) saline. Cisplatin, oxaliplatin, and doxorubicin (hospital pharmacy) were dissolved in 0.9% (w/v) saline. Actinomycin D (Sigma-Aldrich, A1410) was prepared at 1 mg/ml in DMSO, 5,6-Dichlorobenzimidazole 1-β-D-ribofuranoside (DRB, Sigma-Aldrich, D1916) was prepared at 100 mM in DMSO, and cytochalasin B (Sigma-Aldrich, C6762) was prepared at 50 mg/ml in DMSO.

#### siRNA transfection

OVCAR8 GRASP and control cells (2.5 × 10^5^ cells per well) were reverse-transfected in 6-well plates with RAD54L siRNA (sense 5′-AAGCAUUUAUUCGAAGCAUUUUU-3′, antisense 5′-AAAUGCUUCGAAUAAAUGCUUUU-3′) (Table EV3) or ON-TARGETplus Non-targeting Pool (Horizon) at a final concentration of 12.5 µM) using Lipofectamine RNAiMAX (3.75 µL per well) according to the manufacturer’s instructions. At 72 h post-transfection, cells were re-plated and induced with doxycycline (100 ng/mL) for 24 h, then processed for immunoblotting or immunofluorescence analyses.

#### Cell proliferation assays

Drug sensitivity dose–response curves were generated by seeding cells in 96-well plates and allowing cells to adhere for 24 h prior to treatment. Cells were then exposed to vehicle control or increasing concentrations of compounds. Cell growth was monitored using an IncuCyte S3 ZOOM live-cell imaging system (Essen BioScience) by acquiring phase-contrast images every 12 h and quantifying proliferation using the integrated confluence algorithm. Alternatively, at 72 h post-treatment, cells were fixed with methanol and stained with propidium iodide (PI) or DAPI. Cell number was quantified by fluorescent nuclear counting on the IncuCyte S3 ZOOM. Dose–response curves were fitted in GraphPad Prism (v10), and growth inhibition (GI) values were calculated as the concentration required to inhibit net cell growth by 10%, 25%, 50%, 75%, and 90% relative to vehicle controls (GI10, GI25, GI50, GI75, and GI90; 72 h; Table EV1).

#### Clonogenic assays

Cells were seeded in 6-well plates and allowed to adhere for 24 h before drug treatment. Cells were treated with compound(s) for 5 days, after which the media was aspirated, and wells were washed with PBS. Drug-free medium was then added, and cells were cultured for an additional 7 days to permit colony outgrowth. Colonies were fixed in 100% methanol for 30 min, stained with 0.1% (w/v) crystal violet (Sigma-Aldrich, C6158) for 30 min, rinsed thoroughly with H_2_O, and air-dried. Plates were imaged, and colonies were quantified using ImageJ.

#### Cell cycle analysis

Cell cycle distribution was assessed by 5-bromo-2-deoxyuridine (BrdU) incorporation. Cells were pulse-labeled with BrdU (10 μM; Sigma-Aldrich, B5002) for 30 min, washed twice with PBS, harvested, and fixed in ice-cold 80% ethanol. Fixed cells were stored at 4 °C until staining. For BrdU detection, cells were pelleted and incubated in 2 N HCl containing 0.5% (v/v) Triton X-100 for 30 min to denature DNA. Acid was neutralized by washing in 0.1 M sodium tetraborate decahydrate (Na_2_B_4_O_7_.10H_2_O, pH 8.5 (Sigma-Aldrich, S9640). Cells were then incubated sequentially for 30 min with anti-BrdU antibody (Abcam, ab6326) and FITC-conjugated anti-mouse IgG (Jackson ImmunoResearch, 515-095-003) diluted in PBS containing 2% FBS and 0.5% Tween-20. Cells were washed in PBS containing 2% FBS and stained with PI (10 μg/mL) for 15 min at room temperature. Samples were acquired on an LSRFortessa flow cytometer (BD Biosciences), and cell cycle profiles were analyzed using FlowJo (v10).

#### Western blotting

Cells were washed twice with PBS and lysed in SDS lysis buffer (20 mM HEPES, 0.5 mM EDTA, 2% SDS, pH 7.9). Lysates were boiled at 95 °C for 10 min and sheared through a 27-gauge needle. Protein concentration was determined using the detergent-compatible protein assay (Bio-Rad, 5000116). Samples were mixed with 6× SDS sample loading buffer (375 mM Tris-HCl, 9% SDS, 50% glycerol, 0.03% bromophenol blue), re-boiled, and stored at −20 °C until use.

Equal amounts of protein (10-40 µg per lane) were resolved on 4-20% Mini-PROTEAN TGX precast gels (Bio-Rad) in Tris-glycine-SDS running buffer (25 mM Tris, 192 mM glycine, 0.1% SDS, pH 8.7) at 120 V. Proteins were transferred to PVDF membranes in transfer buffer (25 mM Tris, 192 mM glycine, 20% methanol) at 250 mA for 60 min at 4 °C. Membranes were blocked in TBST (TBS, 0.1% Tween-20) containing 5% (w/v) non-fat milk for 1 h at room temperature, incubated with primary antibodies overnight at 4 °C, then washed and incubated with HRP-conjugated secondary antibodies for 1 h at room temperature. Signal was developed using Clarity Western ECL substrate (Bio-Rad) and imaged on an iBright 750 imaging system (Thermo Fisher Scientific).

#### RNA extraction, reverse transcription, and qPCR

Total RNA was extracted using the NucleoSpin RNA Mini kit (Macherey-Nagel, 740955.50) according to the manufacturer’s instructions. First-strand cDNA was synthesized from 100 ng RNA using 0.5 µg random hexamers (Promega, C1181), 10 mM dNTP, 1x SSIV buffer, 0.5 mM DTT, ribonuclease inhibitor 40 U, and SuperScript IV reverse transcriptase 200 U (Thermo Fisher Scientific, 18080044) in 20 µl reaction. Reverse transcription was performed at 25 °C for 10 min, 37 °C for 60 min, followed by enzyme inactivation at 70 °C for 15 min.

Quantitative PCR (qPCR) was performed using Fast SYBR Green Master Mix (Applied Biosystems, 4385610) on a StepOnePlus Real-Time PCR System (Applied Biosystems). Cycling conditions were: 95 °C for 5 min, followed by 39 cycles of 95 °C for 5 s and 60 °C for 30 s. Primer sequences are provided in Table EV4. Relative expression was calculated using the ΔΔCt method, normalized to the indicated reference genes.

#### Strand-specific qPCR

Strand-specific qPCR was performed as described previously (Abraham et al, 2020), using tagged primer-based reverse transcription to selectively amplify strand-specific transcripts. Briefly, cDNA was generated using strand-specific tagged primers (ss_Tag combined with hIGS_forward for sense transcripts; ss_Tag combined with hIGS_reverse for antisense transcripts). 7SK sense transcripts were used as a positive control using 7SK forward primers. Strand-specific primer sequences are provided in Table EV4.

#### Immunofluorescence

For immunofluorescence (IF) with EdU labeling, cells were pulsed with EdU (10 μM) for 30 min to label S-phase cells and then processed as indicated. Cells were fixed in 4% paraformaldehyde (PFA) for 10 min at room temperature, permeabilized with 0.3% Triton X-100 in PBS for 10 min, and blocked in 5% goat serum with 0.3% Triton X-100 in PBS for 30 min. Cells were incubated with primary antibodies for 1 h at 37 °C in a humidified chamber, washed, and incubated with species-appropriate secondary antibodies for 1 h at 37 °C. EdU incorporation was detected using click chemistry in reaction buffer containing 100 mM Tris-HCl (pH 8.5), Alexa Fluor 647-azide (10 nM; Invitrogen, A10277), CuSO4 (1 mM), and ascorbic acid (100 mM) for 30 min at room temperature. Following PBS washes, nuclei were counterstained and mounted using DAPI-containing mounting medium (VECTASHIELD).

For immunofluorescence staining of DNA–RNA hybrids (R-loops), cells were fixed in 4% PFA and then permeabilized with ice-cold 100% methanol for 10 min followed by 100% acetone for 1 min on ice, prior to blocking and incubation with the S9.6 antibody. For RNase H specificity controls, after fixation, permeabilization, and PBS washes, samples were incubated with RNase H (50 U/mL final) at 37 °C for 3 h in a humidified chamber, then processed for blocking and S9.6 staining in parallel with untreated samples.

Images were acquired on a Leica STELLARIS 5 confocal microscope using a ×63 water-immersion objective with a z-step of 1 μm. Image files were processed in ImageJ.

Quantitation: RAD54L, γH2AX, TP53BP1, pRPA (S33), and RAD51 foci were quantified by manual scoring. These DDR factor foci were scored based on localization to nucleolar caps, defined by UBTF staining. Cells with nucleolar caps were identified by the characteristic redistribution of UBTF into peripheral nucleolar cap structures (with 5 or less UBTF foci per nucleolus) following rDNA damage. Nucleolar regions were additionally delineated using NPM staining or the relative absence of DAPI signal, and DDR foci at the nucleolar periphery were quantified. R-loop signal intensity was quantified using CellProfiler.

#### DNA fiber analysis

DNA fiber analysis was performed using sequential incorporation of thymidine analogs followed by molecular combing. Cells (0.75–1.0 × 10^6^) were seeded in 10 cm dishes and allowed to adhere for 24 h. Cells were pulse-labeled with 5-chloro-2-deoxyuridine (CldU 50 μM; Sigma-Aldrich, C6891) for 30 min, washed three times with warm PBS to remove residual label, and then pulse-labeled with 5-iodo-2-deoxyuridine (IdU 250 μM; Sigma-Aldrich, I7125) for 30 min. Labeling steps were performed in a protected area from light. To investigate fork protection and stability, following the second pulse, cells were washed with warm PBS and subsequently treated with 2 mM hydroxyurea for 3 h. For fork speed and analysis of controls, cells were harvested immediately after the IdU pulse.

Cells were collected, and equal numbers of cells were pelleted for each condition. All buffers were prepared fresh on the day of the experiment. Cell pellets were fully resuspended in 45 μL of 1:1 PBS:0.5% trypsin without phenol red (Gibco, 15400054) and incubated at 50 °C for 10 s. The cell suspension was then mixed with 45 μL molten Buffer (1.2% low-melting-point agarose in PBS; Bio-Rad, 1613111) and immediately dispensed into plug molds (Bio-Rad). Plugs were solidified at 4 °C for more than 1 h.

Plugs were released from the mould and incubated in 250 μL of lysis buffer (0.5 M EDTA pH 8.0, 1% sarkosyl, 2 mg/mL proteinase K) overnight at 50 °C to lyse cells and release genomic DNA. Plugs were washed three times for 1 h in 1 mL TE buffer (10 mM Tris-HCl, 0.1 mM EDTA) and then washed once for 3.5 h in wash buffer (1 M Tris, 0.1 M EDTA). Plugs were equilibrated in 0.5 M MES hydrate (pH 5.5, Sigma-Aldrich, M5287), incubated at 68 °C for 20 min followed by 42 °C for 10 min, and then digested with β-agarase (1.5 μL, NEB, M0392S) overnight at 42 °C to release high-molecular-weight DNA.

Released DNA was gently loaded into disposable reservoirs (Genomic Vision, RES-001) containing 0.5 M MES hydrate (pH 5.5) and combed onto silanized coverslips (Genomic Vision, COV-002) using a FiberComb combing instrument (Genomic Vision). Coverslips were mounted onto Superfrost slides using non-fluorescent adhesive. Slides were baked at 65 °C for 2 h, then fibers were denatured in freshly prepared denaturation solution (0.5 M NaOH, 1 M NaCl) for 8 min at room temperature. Slides were washed in PBS and dehydrated through an ethanol series (70%, 90%, and 100%; 5 min each) before air-drying.

Immunodetection of labeled tracks was performed using rat anti-BrdU (BU1/75; Abcam, ab6326; 1:100 in blocking buffer) to detect CldU and mouse anti-BrdU (BD Biosciences, 347580; 1:50) to detect IdU, followed by species-appropriate fluorescent secondary antibodies. Washes were performed in PBS containing 0.1% Triton X-100. Fibers were imaged on a Leica THUNDER microscope using a ×40 objective, and tract lengths were measured manually in ImageJ.

#### Chromatin immunoprecipitation (ChIP)

Following doxycycline induction (100 ng/mL, 24 h), GRASP and control cells were fixed in culture medium containing 0.6% PFA at 37 °C for 10 min. Crosslinking was quenched with glycine (0.125 M) for 10 min at room temperature. Cells were washed twice with PBS, scraped, and pelleted (3000× *g*, 5 min). The pellet containing 6 × 10^6^ cells was resuspended in 300 μl hypotonic lysis buffer (10 mM Tris-HCl pH 7.4, 10 mM NaCl, 10 mM MgCl_2_, 0.5% IGEPAL CA-630 in DEPC-treated water) supplemented with protease inhibitors and incubated on ice for 10 min. Nuclei were pelleted (9000× *g*, 3 min, 4 °C), resuspended in 300 μl MNase digestion buffer, and digested with micrococcal nuclease (MNase, NEB M0247; 250 gel units for 10 min in OVCAR8 and 500 gel units for 20 min in U2OS cells) at 37 °C with brief vortexing every 2.5 min. Digestion was stopped by the addition of EDTA (final 50 mM), and samples were placed on ice for 5 min. Nuclei were pelleted (9000× *g*, 5 min, 4 °C) and resuspended in 300 μl ChIP dilution buffer (167 mM NaCl, 16.7 mM Tris-HCl pH 8.1, 0.01% SDS, 1.1% Triton X-100, 1 mM DTT in DEPC-treated water) supplemented with protease inhibitors. Chromatin was further fragmented by sonication using a Branson Sonifier (40% duty cycle; three rounds of 6 s on/30 s off). Insoluble material was removed by centrifugation (9000× *g*, 10 min, 4 °C), and supernatants were retained.

For immunoprecipitation, chromatin was diluted to 1 mL in ChIP dilution buffer; 6% chromatin was reserved as input and stored at −80 °C. Pol I and Pol II antibodies (3–5 μg) were added, and samples were incubated overnight at 4 °C with rotation. Immune complexes were captured using protein A/G magnetic beads (Thermo Fisher Scientific, 88803) for 2 h at 4 °C. Beads were washed sequentially with low-salt wash buffer (150 mM NaCl, 20 mM Tris-HCl pH 8.1, 2 mM EDTA, 1% Triton X-100, 0.1% SDS), high-salt wash buffer (500 mM NaCl, 20 mM Tris-HCl pH 8.1, 2 mM EDTA, 1% Triton X-100, 0.1% SDS), LiCl wash buffer (0.25 M LiCl, 10 mM Tris-HCl pH 8.1, 1 mM EDTA, 1% sodium deoxycholate, 1% NP-40), and twice with TE (10 mM Tris-HCl pH 8.1, 1 mM EDTA). Chromatin was eluted twice in 250 μl elution buffer (100 mM NaHCO_3_, 1% SDS) for 15 min at room temperature with rotation. Inputs were brought to the same final volume (500 μl) using elution buffer. Crosslinks were reversed by incubation with NaCl (final 200 mM) at 65 °C overnight.

Following reverse crosslinking, samples were treated with proteinase K (20 μg) in the presence of EDTA (final 10 mM) and Tris-HCl (final 40 mM, pH 6.5) for 2 h at 45 °C. DNA was purified by 500 μl phenol:chloroform:isoamyl alcohol extraction (25:24:1) and 500 μl isopropanol precipitation with sodium acetate (3 M, 50 μl) and glycogen carrier (20 μg). Pellets were washed with 70% ethanol, air-dried, and resuspended in DEPC-treated water. ChIP enrichment was quantified by qPCR using primers listed in Table EV4 and expressed as % input and/or fold enrichment relative to IgG controls (state which metric is used in figures).

#### DNA–RNA immunoprecipitation (DRIP) followed by qPCR (DRIP–qPCR)

DRIP–qPCR was performed largely as described by Sanz et al (Sanz and Chedin, 2019) with minor modifications. Briefly, cells were harvested at about 75–80% confluency from 15-cm dishes (7–8 × 10^6^ cells per sample), washed in 1× DPBS, pelleted (1000 rpm, 3 min), and resuspended in 1.6 mL TE buffer. SDS (50 µL of 20% w/v) and proteinase K (5 µL of 20 mg/mL) were added, and lysates were mixed by gentle inversion until viscous, then incubated overnight at 37 °C.

Genomic DNA was extracted by phenol/chloroform/isoamyl alcohol (25:24:1), ethanol precipitated (1/10 volume 3 M sodium acetate pH 5.2, and 2.5 volumes 100% ethanol), and DNA was gently spooled using cut tips to minimize shearing. DNA was washed three times with 80% ethanol, air-dried, and resuspended in TE (125 µL) on ice with minimal pipetting using cut tips. Genomic DNA was then digested overnight at 37 °C using a restriction enzyme cocktail (BsrGI-HF, EcoRI, HindIII, SspI-HF, and XbaI; 1.5 µL each of 20 U/µL) in 1× NEB Buffer 2 with 1× BSA; spermidine (final 0.5×) was added last. Digestion efficiency was verified by electrophoresis on a 0.8% agarose gel. Digested DNA was extracted using phenol/chloroform and ethanol precipitation (with glycogen carrier), washed with 80% ethanol, air-dried, and resuspended in TE. DNA concentration was determined by spectrophotometry; ≥28 µg total digested DNA per sample was prepared for RNase H-treated controls and immunoprecipitation reactions.

For assessing the specificity of the assay in detecting R-loops, 10 µg of digested DNA was treated with RNase H at 37 °C for 4 h. For immunoprecipitation, 10 µg of digested DNA was diluted to 450 µL in TE and combined with 52 µL of 10× DRIP binding buffer (1× final) and either S9.6 antibody (10 µg) or rabbit IgG control antibody (10 µg). A 10% input fraction was retained prior to antibody addition. Samples were incubated overnight at 4 °C on a rotating wheel. The following day, 25 µL of protein A/G magnetic beads per IP were pre-washed in TE, added to each reaction, and incubated for 2 h at 4 °C with rotation. Beads were washed twice with 1× binding buffer (750 µL per wash; 15 min at room temperature with rotation). Bound nucleic acids were eluted in DRIP elution buffer (300 µL) supplemented with proteinase K (7 µL of 20 mg/mL) for 45 min at 55 °C. Eluates were recovered on a magnetic rack and purified by phenol/chloroform extraction followed by ethanol precipitation with glycogen, then resuspended in 50 µL TE.

Enrichment of DNA–RNA hybrids at selected loci was quantified by qPCR (primer sequences in Table EV4) using input-normalized values. DRIP signal was reported as % input and/or as fold enrichment of S9.6 over IgG, with RNase H-treated samples used to confirm R-loop pull-down specificity.

#### DDR CRISPR-Cas9 synthetic lethal screen

To identify drug-gene synthetic lethal interactions within the DNA damage response (DDR) network, OVCAR8 cells stably expressing Cas9 were reverse-transfected with crRNA:tracrRNA complexes targeting individual DDR genes (crRNA duplexes; Millennium Science) using DharmaFECT 4 (Horizon, 31985070) together with tracrRNA (Horizon, U-002000). A non-targeting sgRNA control was included in parallel. After 24 h, the transfection medium was replaced with complete growth medium, and cells were cultured for a further 72 h to enable gene editing.

Edited cells were then re-plated into 384-well plates using a BioTek liquid handler and treated with a panel of 11 compounds at GI10, GI25, and GI50 concentrations (three technical replicates per condition). Drug concentrations were defined from independent dose–response assays (Table EV1). After 72 h of drug exposure (or at the screening endpoint; specify), cells were fixed, stained with DAPI, and imaged on a Cellomics CX7 LZR high-content platform (Thermo Fisher Scientific) using a ×10 objective. Cell survival was quantified from DAPI-positive nuclear counts.

Synthetic lethal (SL) scores were calculated by first determining the fold change in proliferation for each gene knockout under drug treatment relative to the corresponding DMSO-treated knockout. Two complementary metrics were then derived for each gene–drug pair: SL_divid (relative reduction), defined as the proportional change in drug response in the gene knockout relative to parental OVCAR8-Cas9 cells treated with the same compound; and SL_minus (absolute reduction), defined as the absolute difference in drug response between the gene knockout and parental OVCAR8-Cas9 cells. Candidate SL interactions were nominated using combined thresholds of SL_minus > 0.15 and SL_divid <0.85.

#### Statistics and reproducibility

No statistical method was used to predetermine the sample size, but we routinely, unless stated, employed at least three biological repeats for each experiment. In imaging experiments, scoring and quantitation included 50–100 cells per condition/per experiment. Randomization was not appropriate because samples were derived from specific genetic cell lines or treatment conditions. The investigators were not blinded to allocation during the experiments and outcome assessment because all numerical data were software-automated and independent of investigator subjectivity. No data were excluded from any analyses. All data presented in the manuscript are represented as the mean ±  SEM or SD and were analyzed using GraphPad Prism (version 10). The statistical test employed for each dataset has been specified in the figure legends.

## Supplementary information


Appendix
Table EV1
Table EV2
Table EV3
Table EV4
Peer Review File
Source data Fig. 1
Source data Fig. 2
Source data Fig. 3
Source data Fig. 4
Source data Fig. 5
Source data Fig. 6
Source data Fig. 7


## Data Availability

This study includes no data deposited in external repositories. All data are available in the manuscript and the corresponding Source Data files. Material is available upon request from the corresponding author. The source data of this paper are collected in the following database record: biostudies:S-SCDT-10_1038-S44318-026-00864-3.
