# Supplementary material for: RAD54L coordinates the nucleolar DNA damage response to maintain rDNA stability
Source: EMBO J. 2026 Jul 23;45(17):6299–319. doi: 10.1038/s44318-026-00864-3 (PMC13534639; doi:10.1038/s44318-026-00864-3)
Supplement: Supplementary file 1 [file 44318_2026_864_MOESM1_ESM.pdf]

Appendix for

"RAD54L coordinates the nucleolar DNA damage response to maintain rDNA stability"

Ruofei Liu, Jiachen Xuan, Carmelo Cerra, Nyree Stojnic, Junqi Pan, Shalini Sundramurthi Chelliah, Shannon Mendez, Rhynelle Dmello, Karla J. Cowley, Yangyi Zhang, Kezia Gitareja, Matthew Wakefield, Andrew Deans, Clare Scott, Keefe T. Chan, Kaylene J. Simpson, Jian Kang and Elaine Sanij

Table of Content: **Page**

Appendix Figure S1	2
Appendix Figure S2	4
Appendix Figure S3	7
Appendix Figure S4	8
Appendix Figure S5	10
Appendix Figure S6	11

Appendix Figure S1

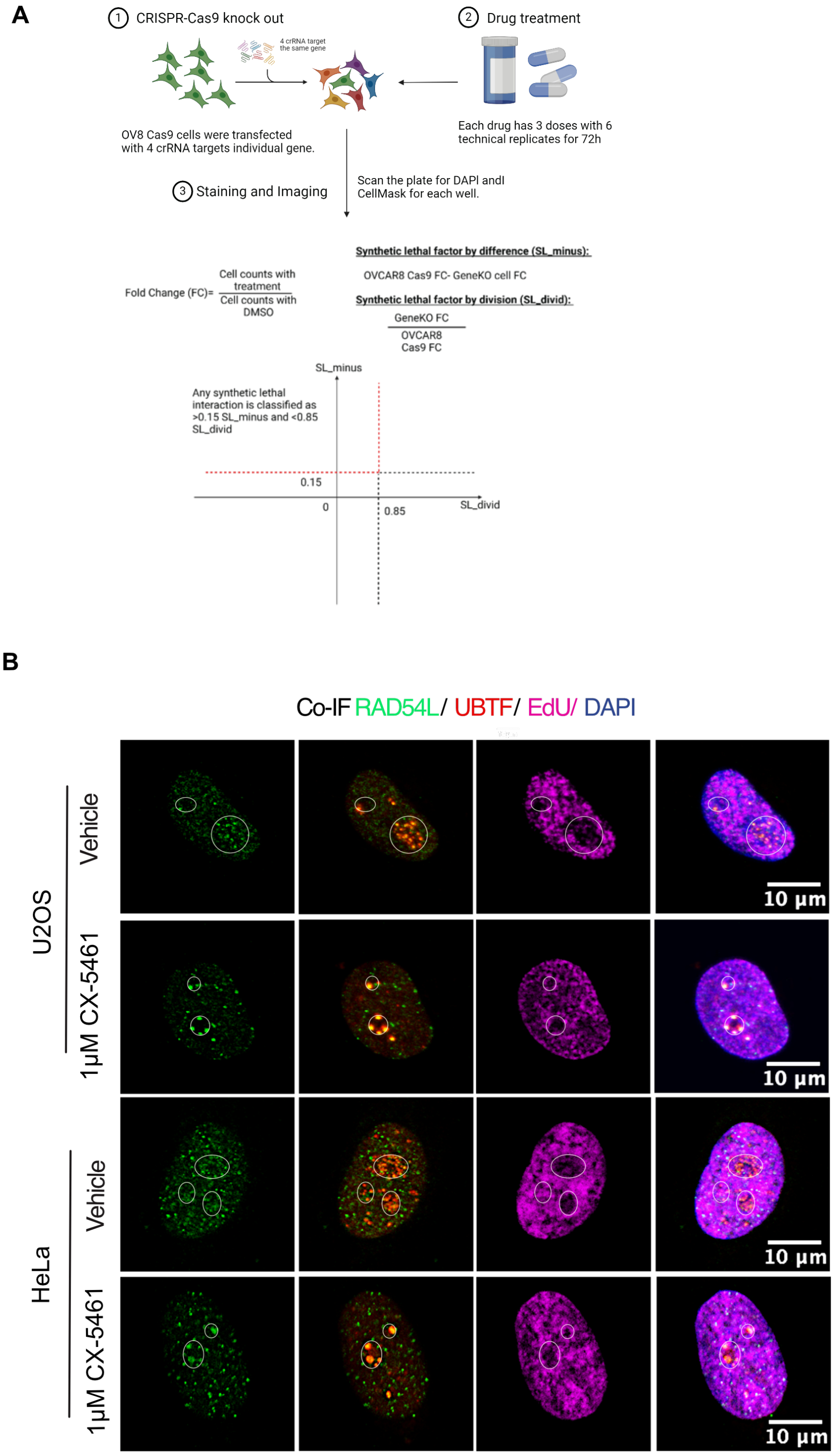

Appendix Figure S1. A boutique CRISPR-Cas9 synthetic lethal (SL) screen.
(A) Schematic overview of the screening workflow. OVCAR8 Cas9-expressing cells were reverse-transfected with gene-specific crRNAs (Reagents and Tools Table). After 96 h, cells were replated and treated with the indicated drugs (listed in Table EV1) for 72 h, then fixed,

stained with DAPI, and imaged. Cell survival was quantified by automated cell counting of DAPI stained nuclei. SL interactions were identified by first calculating the fold change in proliferation of drug-treated knockout (KO) cells relative to DMSO-treated cells for each gene KO. Two SL metrics were then derived for each gene-drug pair: SL_divid (relative reduction), defined as the proportional change in the KO response to drug relative to the corresponding response in parental OVCAR8 Cas9 cells; and SL_minus (absolute reduction), defined as the absolute difference in drug response between the KO and parental OVCAR8 Cas9 cells. Hits were called using stringent thresholds of SL_minus > 0.15 and SL_divid < 0.85.
(B) Co-IF analysis of RAD54L and UBTF in U2OS and HeLa cells treated with CX-5461 (1µM) or vehicle for 3 h. Representative images from three independent experiments. Quantification of RAD54L foci at nucleolar caps (marked with UBTF) is shown in Figure 2B. White circles represent nucleolar periphery.

Appendix Figure S2

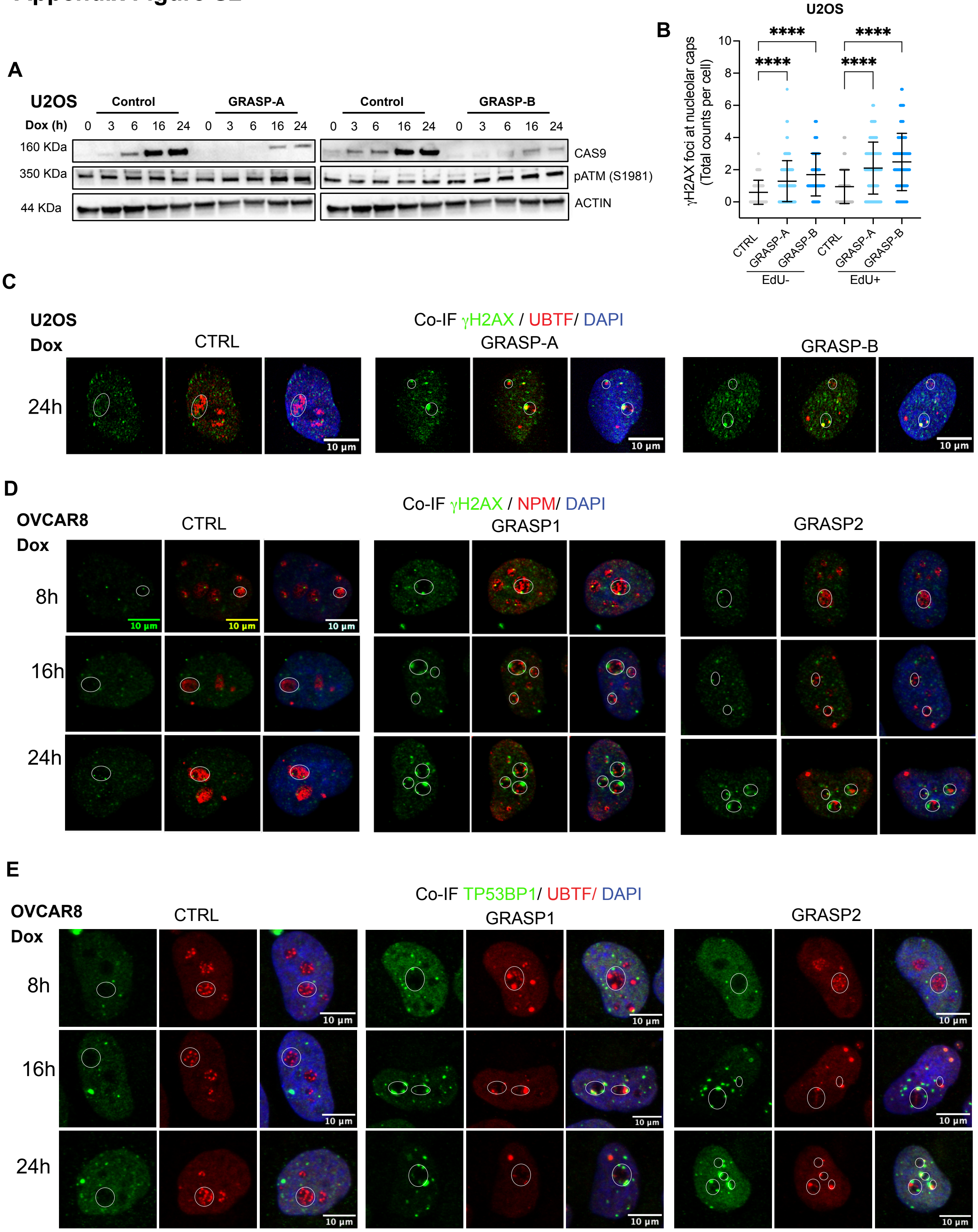

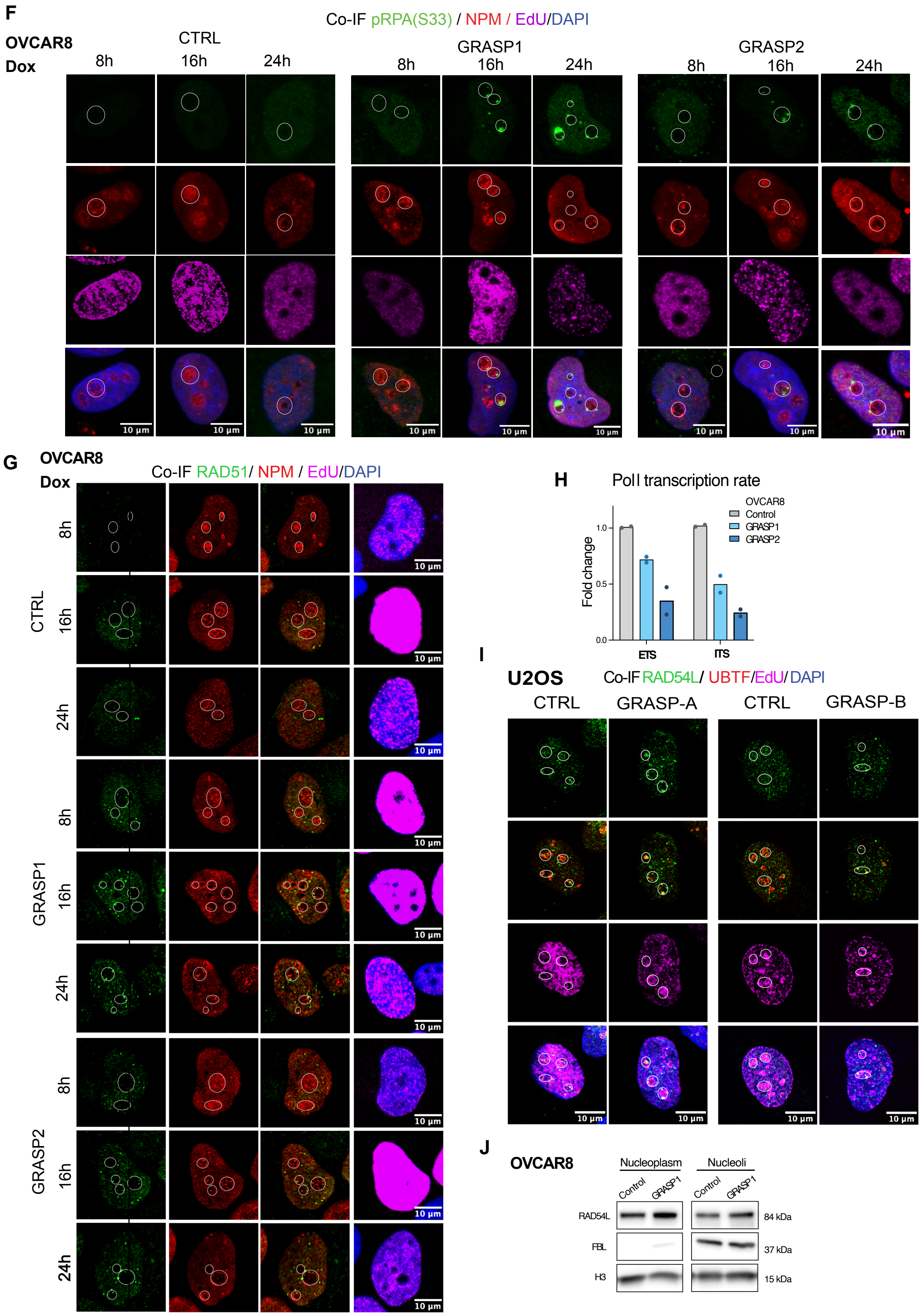
F
OVCAR8
Dox
Co-IF pRPA(S33) / NPM / EdU/DAPI
CTRL
GRASP1
GRASP2
8h
16h
24h
10 µm
G
OVCAR8
Dox
Co-IF RAD51/ NPM / EdU/DAPI
CTRL
GRASP1
GRASP2
8h
16h
24h
10 µm
H
PolI transcription rate
OVCAR8
Control
GRASP1
GRASP2
Fold change
1.0
0.5
0.0
ETS
ITS
I
U2OS
Co-IF RAD54L/ UBTF/EdU/ DAPI
CTRL
GRASP-A
CTRL
GRASP-B
10 µm
J
OVCAR8
Nucleoplasm
Nucleoli
Control
GRASP1
Control
GRASP1
RAD54L
FBL
H3
84 kDa
37 kDa
15 kDa

Appendix Figure S2. RAD54L functions as a nucleolar DNA damage response factor.

(A) Western blot analysis of Cas9 and pATM (S1981) in U2OS cells treated with doxycycline (100 ng/ml) as indicated. Actin served as a loading control. Blots are representative of three independent biological replicates. (**B**) Quantification of γH2AX foci at nucleolar cap (marked with UBTF) in U2OS control and GRASP cells treated with doxycycline (100 ng/ml) for 24 h.

Cells were pulse-labelled with EdU for 30 min prior to endpoint to mark replicating cells. Error bars represent mean ± SD, $n > 50$ EdU- cells and $n > 75$ EdU+ cells per condition from two independent experiments, **** $p < 0.0001$ using one-way ANOVA with Tukey's multiple comparisons test. (**C**) Co-IF analysis of γH2AX and UBTF in U2OS control and GRASP cells treated as (B). White circles represent nucleolar periphery. Representative images from three independent experiments. (**D**) Co-IF analysis of γH2AX with NPM in OVCAR8 control and GRASP cells upon doxycycline induction (100 ng/ml) for 8 h, 16 h or 24 h. Cells were pulse-labelled with EdU to mark replicating cells. Representative images from three independent biological experiments. Quantification of γH2AX foci at nucleolar caps (marked with NPM) is shown in Figure 2E. (**E**) Co-IF analysis of TP53BP1 with UBTF in OVCAR8 control and GRASP cells treated as in (D). Representative images from three biologically independent experiments. Quantification of TP53BP1 foci at nucleolar caps (marked with UBTF) is shown in Figure 2F.

White circles represent nucleolar periphery (**C-E**).

(**F**) Co-IF analysis of pRPA(S33) with NPM in OVCAR8 control and GRASP cells treated as in (D). Representative images from three independent biological experiments. Quantification of pRPA(S33) foci count at nucleolar caps (marked with NPM) is shown in Figure 2G.

G) Co-IF analysis of RAD51 with NPM in OVCAR8 control and GRASP cells treated as in (D). Representative images from three biologically independent experiments. Quantification of RAD51 foci count at nucleolar caps (marked with NPM) is shown in Figure 2H.

(**H**) Quantitative real-time PCR analysis of RNA Pol I transcriptional rate in OVCAR8 control and GRASP cells using primers targeting the 5'ETS and ITS of the 47S pre-rRNA. Expression was normalized to *Vimentin* mRNA and is presented as fold change relative to control OVCAR8 cells, $n = 2$.

(**I**) Co-IF analysis of RAD54L and UBTF in U2OS control and GRASP cells upon doxycycline induction (100 ng/ml) for 24 h. Representative images from three independent experiments.

(**J**) Western blot analysis of RAD54L in nucleolar and nucleoplasmic fractions from OVCAR8 control and GRASP1 cells following doxycycline induction (100 ng/ml) for 24 h. FBL served as a loading control for nucleolar fraction and histone H3 served as a loading control for both nucleoplasmic and nucleolar fractions.

White circles represent nucleolar periphery (**F-G, I)**.

Appendix Figure S3

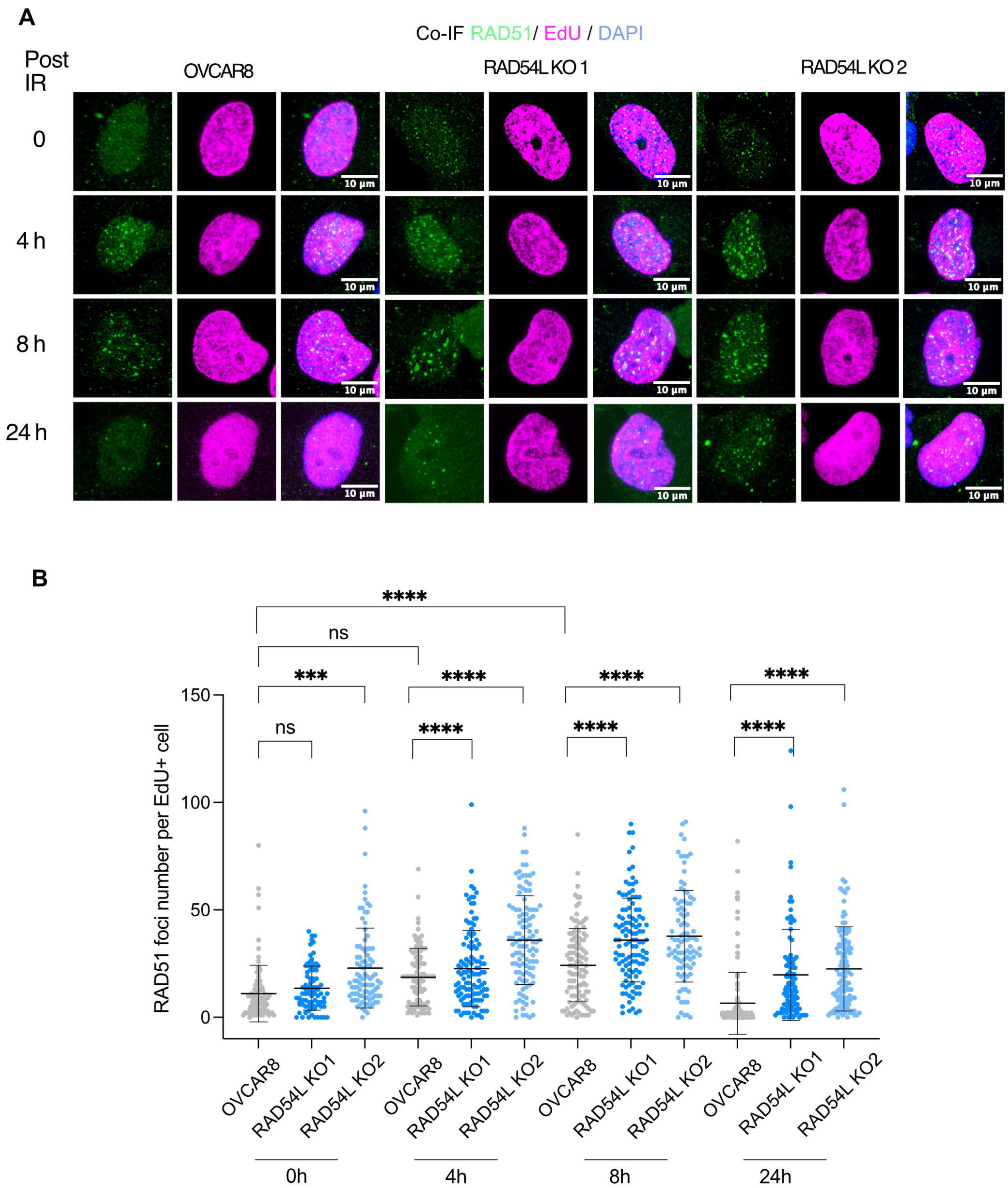

Appendix Figure S3. RAD54L loss leads to elevated RAD51 foci formation following ionizing radiation.

(**A**) Co-IF analysis of RAD51 foci in parental OVCAR8 and RAD54L KO cells, under basal condition or following ionizing irradiation (IR; 5 Gy). Cells were pulse labelled with EdU (10 µM, 30 min) prior to IR to mark replicating cells. Cells were fixed and stained at 4, 8, 24 h post-IR. Representative images from two independent experiments.

(**B**) Quantification of Nuclear RAD51 foci in EdU positive cells using CellProfiler. Data are presented as mean ± SD. $n > 150$ EdU+ cells per condition across two independent replicates. Statistical analysis was performed using one-way ANOVA with Tukey's multiple comparisons test; ns, non-significant; *** $p = 0.003$; **** $p < 0.0001$.

Appendix Figure S4

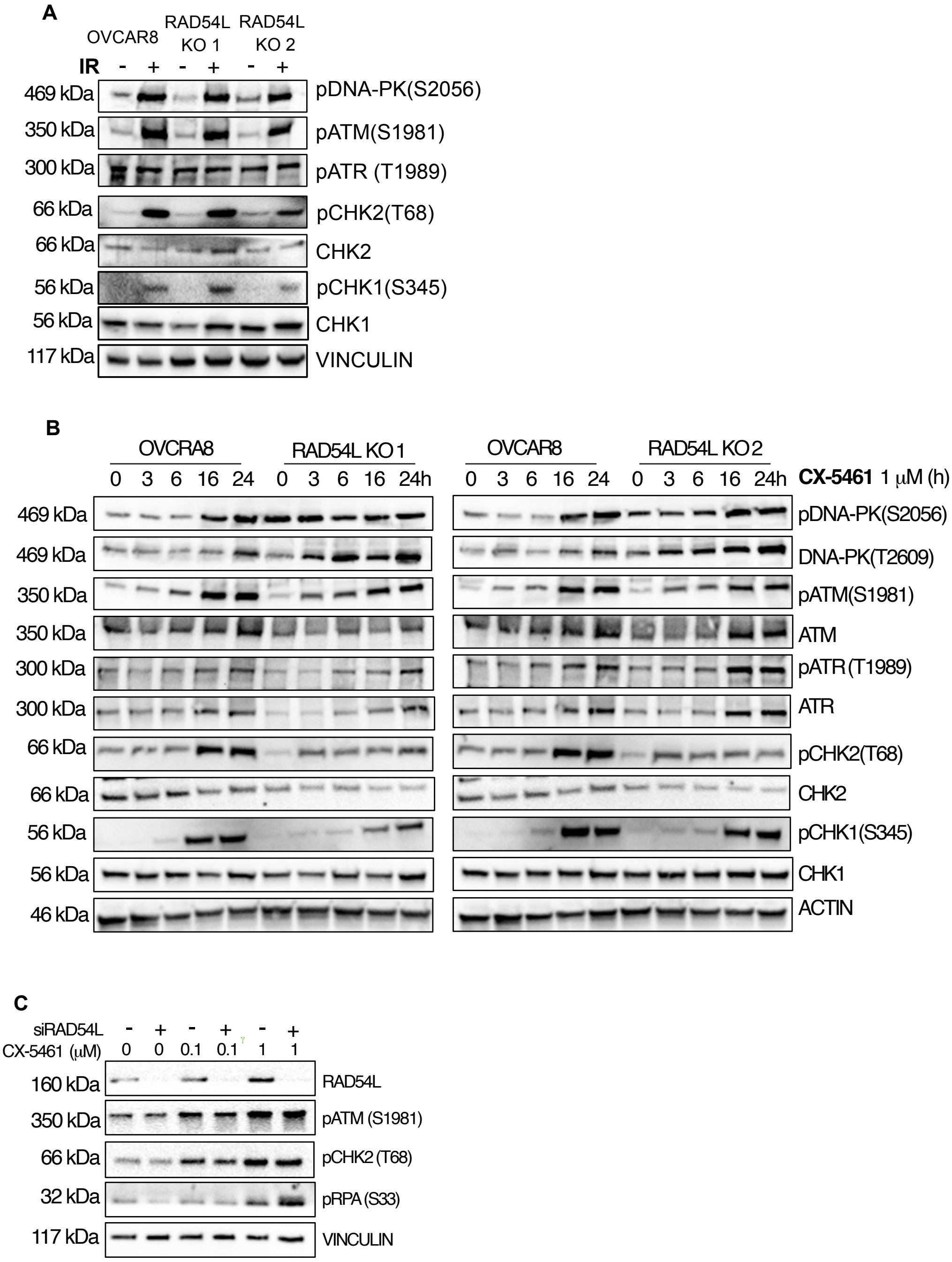

Appendix Figure S4. Loss of RAD54L attenuates ATM-CHK2 signaling upon CX-5461 treatment.

(**A**) Western blot analysis of ATM, ATR and DNA-PK signaling in parental OVCAR8 and RAD54L KO cells, under basal conditions or 4 h post-ionizing irradiation (5 Gy). Vinculin served as a loading control.

(**B**) Western blot analysis of ATM, ATR and DNA-PK signaling in parental OVCAR8 and RAD54L KO cells treated with CX-5461 for various time points as indicated. Actin served as a loading control.

(**C**) Western blot analysis of ATM signaling and pRPA S33 in OVCAR8 transfected with siRAD54L or non-targeting control siRNA, followed by CX-5461 (0.1 or 1 μM) for 24 h. Vinculin served as a loading control.

All blots are representative of three biological replicates.

Appendix Figure S5

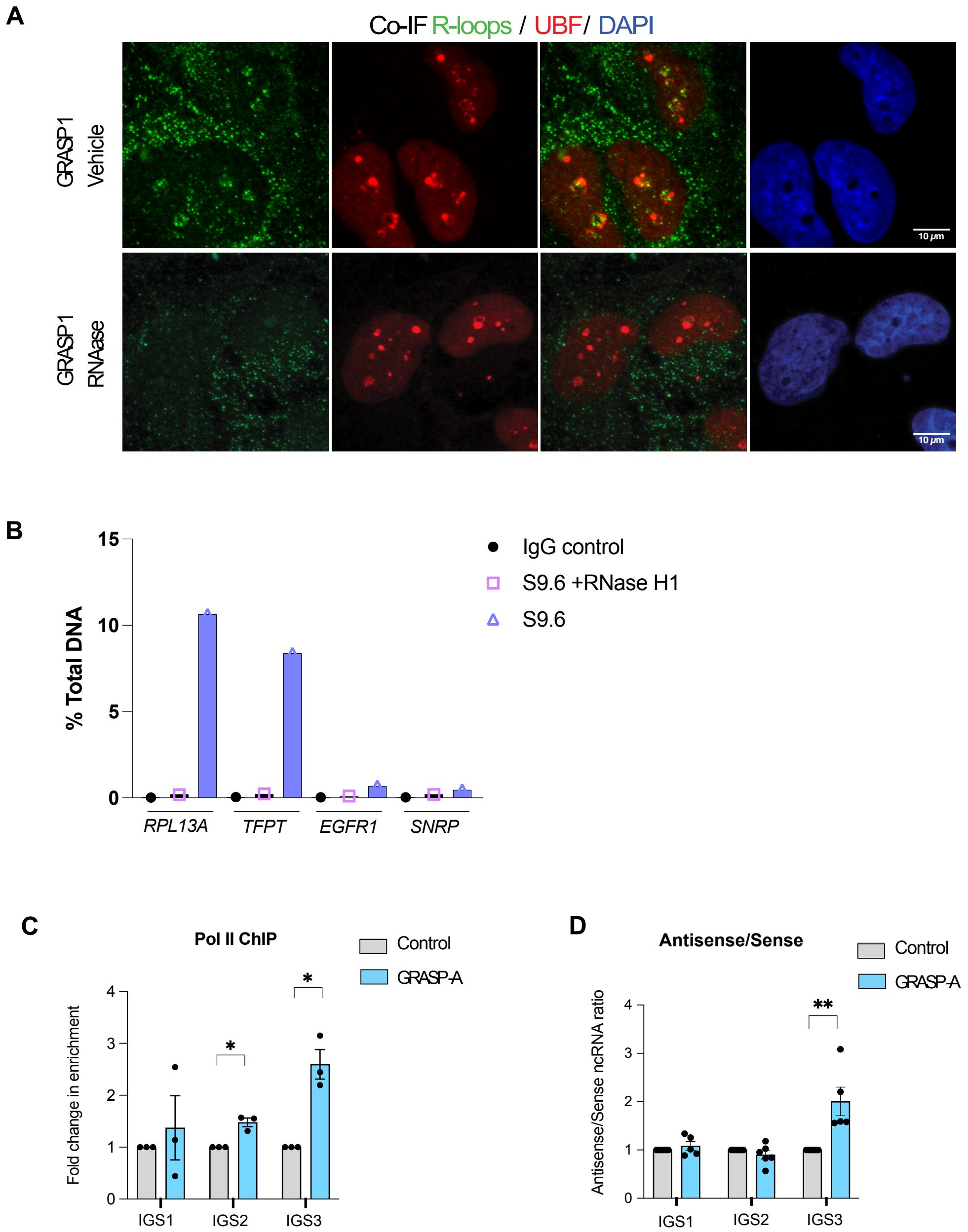

Appendix Figure S5. Pol II transcription activity is associated with nucleolar DDR.

(**A**) Co-IF analysis of R-loops and UBTF in OVCAR8 GRASP1 cells treated with doxycycline (100 ng/ml) for 24 h, followed by vehicle or RNase H (5 U) for 3 h to degrade R-loops. (**B**) DRIP analysis in OVCAR8 cells confirms the specificity of R-loop signal that was enriched at R-loop-positive foci (RPL13A and TFPT) and abolished by RNase H treatment, whereas EGR1 and SNRP served as negative control loci. (**C**) ChIP-qPCR analysis of Pol II binding to rDNA IGS region of U2OS control and GRASP cells, $n = 3$, $*p = 0.0294$ (IGS2), $*p = 0.0305$ (IGS3). (**D**) Strand-specific RT–qPCR (ss-RT) analysis of sense and antisense intergenic ncRNAs transcribed from rDNA IGS regions. The antisense-to-sense intergenic ncRNAs ratio was calculated for each site, and the relative ratio was calculated by normalizing the ratio of U2OS GRASP cells to control cells within each experiment. $n = 5\text{-}6$, $**p = 0.002165$. Statistical significance was assessed by unpaired t-test (**C-D**). Error bars represent mean ± SEM (**C-D**).

Appendix Figure S6

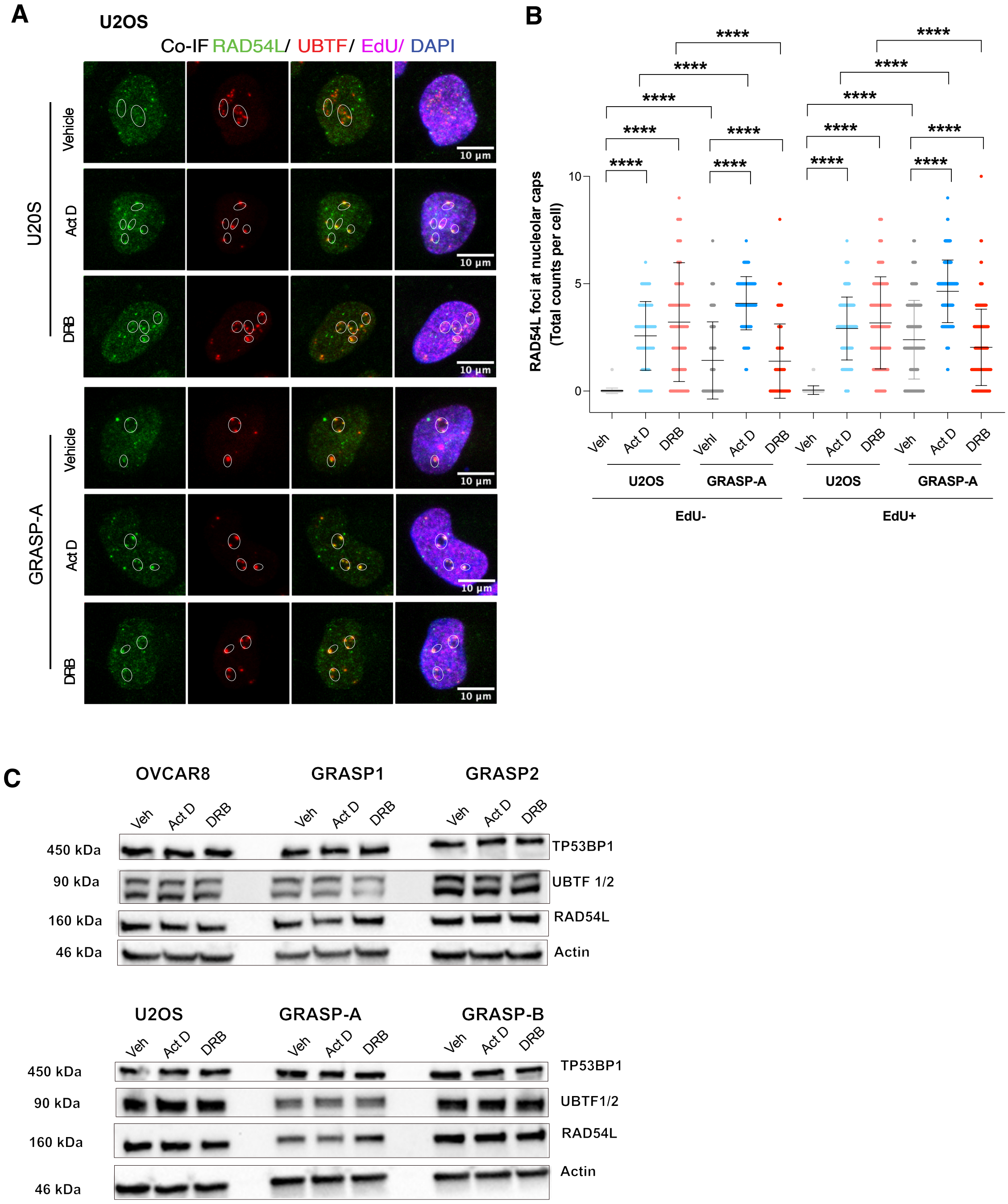

Appendix Figure S6. Pol II transcription activity is linked to nucleolar reorganization and recruitment of repair factors to nucleolar caps.

(**A**) Co-IF analysis of RAD54L with UBTF in U2OS control and GRASP-A cells. Cells were treated with doxycycline (100 ng/ml) for 24 h. Actinomycin D (10 nM) or DRB (100 µM) was added 4 h before the endpoint. Representative images from two independent experiments. White circles represent nucleolar periphery.

(**B**) Quantification of RAD54L foci at nucleolar caps (marked with UBTF) in U2OS control and GRASP-A cells treated as (A). $n > 70$ EdU- cells per condition and $n > 100$ EdU+ cells per condition across two independent experiments, **** $p < 0.0001$. Error bars represent mean ±

SD. Statistical significance was assessed by one-way ANOVA with Tukey's multiple comparisons test.

(**D**) Western blot analysis of RAD54L and TP53BP1 in control and GRASP cells treated as (A). Actin serves as a loading control.
