# Supplementary material for: RAD54L coordinates the nucleolar DNA damage response to maintain rDNA stability
Source: EMBO J. 2026 Jul 23;45(17):6299–319. doi: 10.1038/s44318-026-00864-3 (PMC13534639; doi:10.1038/s44318-026-00864-3)
Supplement: Supplementary file 2 [file 44318_2026_864_MOESM6_ESM.pdf]

THE EMBO JOURNAL

RAD54L coordinates the nucleolar DNA damage response to maintain rDNA stability

Ruofei Liu, Jiachen Xuan, Carmelo Cerra, Nyree Stojnic, Junqi Pan, Shalini Sundramurthi Chelliah, Shannon Mendez Ruiz, Rhynelle Dmello, Karla Cowley, Yangyi Zhang, Kezia Gitareja, Matthew Wakefield, Andrew Deans, Clare Scott, Keefe Chan, Kaylene Simpson, Jian Kang, and Elaine Sanij

Corresponding author(s): Elaine Sanij (esanij@svi.edu.au)

Review Timeline:

Submission Date:	30th Apr 26
Editorial Decision:	21st May 26
Revision Received:	4th Jul 26
Accepted:	7th Jul 26

Editor: Hartmut Vodermaier

Transaction Report:

(Note: With the exception of the correction of typographical or spelling errors that could be a source of ambiguity, letters and reports are not edited. Depending on transfer agreements, referee reports obtained elsewhere may or may not be included in this compilation. Referee reports are anonymous unless the Referee chooses to sign their reports.)

This manuscript was transferred to The EMBO JOURNAL following peer review at another journal.

Reviewers' comments:

Reviewer #1 (Remarks to the Author):

The manuscript by Liu et al. identifies a synthetic lethal interaction between RNA Pol I inhibitors and RAD54L. They show that RAD54L associates with rDNA and relocalizes to nucelolar caps upon rDNA DSBs induced by Cas9. Depletion of RAD54L induces micronuclei, which they suggest are associated with replication stress. In response to CX-5461ATM-CHK2 signalling is attenuated in Rad54L KO cells, preventing transcriptional repression and cap formation. Fork speed is increased and fork protection decreased in response to HU in RAD54L KO. The authors suggest that RAD54L protects against replication stress by constraining R-loop formation induced by RNA Pol II transcription in rDNA. The authors present interesting results on induction of RNA Pol II-dependent transcription at rDNA intergenic regions upon rDNA DSBs and present data that may indicate a role of RNA Pol II transcription in nucleolar restructuring in response to rDNA DSBs. Although the manuscript has improved during the revision, and still contains interesting observations, the major issue of the study not being coherent remains. The authors change back and forth between Cas9 induced DSBs and treatment with CX-5461, and try to assemble this into one story. The fundamental problem is clearly reflected in the model presented by the authors, where it is clear that the pieces are not coming together. Unfortunately, the study cannot be recommended for publication

Below are specific comments that may help the authors in their future work, however they do not overcome the primary concern of the paper:

The concerns raised by Reviewer 1 appear to centre largely on issues of narrative framing and interpretation, rather than on the validity or robustness of the experimental work. Importantly, these concerns are, in our view, addressable through clarification. In particular, we believe Reviewer 1 may have missed a central point of the study regarding our use of two complementary experimental models. The Cas9-induced rDNA DSB system and CX-5461 treatment were intentionally used to interrogate distinct forms of nucleolar stress, and together they show that reliance on RAD54L in the nucleolus differs according to the nature of the insult. Reviewer 1's comment that "The authors change back and forth between Cas9 induced DSBs and treatment with CX-5461, and try to assemble this into one story" appears, in our view, to reflect a conceptual misunderstanding of the experimental framework rather than a flaw in the data themselves.

We have revised the manuscript to reflect the intentional use of the two experimental systems and highlight that our findings establish RAD54L as a key regulator that couples replication stress response to DNA repair pathways to maintain rDNA stability and nucleolar genome integrity.

• Fig. 2: RAD54L appears nucleolar in untreated cells and seems associated with rDNA. Can the authors clarify whether nucleolar RAD54L is present across all cell-cycle

phases, independent of EdU status, or whether it is enriched specifically in S phase in the absence of exogenous damage? Is there a measurable increase in nucleolar RAD54L during S phase? Is RAD54L being recruited in response to rDNA damage or is it just condensed and therefore appear brighter? Addressing these points would help define RAD54L's nucleolar role and its place within the n-DDR.

In Fig 2A-B and Fig 1SB, the data show that RAD54L is present in nucleoli under untreated (baseline) conditions, where it exhibits punctate staining that co-localizes with UBTF in OVCAR8, U2OS, and HeLa cells. This basal nucleolar localization is observed in both EdU-negative and EdU-positive cells, indicating that nucleolar localisation of RAD54L is **not restricted to S phase**. These findings indicate that RAD54L is a constitutive nucleolar factor, rather than being exclusively recruited following rDNA damage.

We agree that the current manuscript underemphasises this aspect, as the primary focus has been on RAD54L's role in replication stress responses and DNA repair.

Our data in Fig 5A-B show that RAD54L contributes to **replication fork restraint and stability**. RAD54L-deficient cells display increased fork speed, reduced fork stability, elevated nucleolar pRPA(S33) (Fig 5C-D) and R-loops (Fig 5E-F), all consistent with defective control of replication-associated stress at rDNA. Together, these findings support a model in which the basal nucleolar pool of RAD54L performs a surveillance function, stabilising replication forks and limiting ssDNA/R-loop accumulation at highly transcribed rDNA repeats, even in the absence of exogenous damage.

To determine whether RAD54L is involved in rDNA repair, we quantified **RAD54L foci at nucleolar caps** in EdU-negative and EdU-positive cells following CX-5461 treatment and after induction of rDNA DSBs in the GRASP system. In both conditions, RAD54L exhibited a clear increase in discrete cap-associated foci, rather than a uniform increase in nucleolar intensity, indicating spatial redistribution to sites of damage. Consistently, nucleolar fractionation followed by immunoblotting demonstrated increased RAD54L levels in nucleolar fractions after rDNA DSB induction (Fig. S2J), supporting active recruitment.

Together, these results distinguish regulated recruitment/enrichment of RAD54L at damaged rDNA from passive concentration due to nucleolar condensation.

Thus, RAD54L exhibits dual behaviour: a constitutive nucleolar presence consistent with a surveillance role during normal rDNA transcription/replication, and damage-induced enrichment at nucleolar caps, supporting its active function in rDNA repair and the nucleolar DNA damage response (nDDR).

• "RAD54L accumulated at nucleolar caps within 24 h of rDNA DSB induction in OVCAR8 (Fig. 2I–J) and U2OS cells (Fig. 2J, Fig. S2I), independent of replication status." In OVCAR8 there seems to be a preference for EdU+, whereas in U2OS GRASPB (note mislabelling) enrichment is evident only in EdU-positive cells. Given these cell-line and EdU-dependent differences, the conclusion about replication-status independence should be re-evaluated.

We thank the reviewer for this careful observation. We agree that the relationship between RAD54L recruitment and replication status requires cautious interpretation, particularly given the differences observed between cell lines and clones. RAD54L foci at nucleolar caps were quantified in both EdU-positive and EdU-negative cells in OVCAR8 and U2OS GRASP clonal cell lines (Fig 2I–J).

In OVCAR8 cells, RAD54L accumulation at nucleolar caps is more prominent in EdU-positive cells, suggesting a degree of enrichment during S phase. In contrast, in U2OS GRASP cells, RAD54L recruitment is detectable in both EdU-positive and EdU-negative populations, although the magnitude of enrichment is more variable and less pronounced in EdU-negative clone B cells. We interpret these differences as reflecting cell line- and clone-specific variability in rDNA damage induction and nucleolar reorganisation, rather than indicating a strict dependence on S phase. Importantly, RAD54L accumulation at nucleolar caps is consistently observed in EdU-negative cells across both systems, demonstrating that recruitment can occur outside of active DNA replication.

To avoid overstating this point, we have revised the text from "independent of replication status" to more precise wording:
"RAD54L accumulated at nucleolar caps in both EdU-positive and EdU-negative cells, indicating that its recruitment is not restricted to S-phase cells."

• Fig. 3: There is a clear preference for S-phase cells, with RAD51 foci essentially absent in EdU-negative cells. How do the authors reconcile this with the current model of the n-DDR (first presented by the McStay lab)?

We thank the reviewer for this important point. We agree that RAD51 recruitment to nucleolar caps can occur outside S phase, as demonstrated the McStay laboratory (Van Sluis & McStay, G&D 2015).

We agree that RAD51 foci at nucleolar caps are strongly enriched in EdU+ cells in our system. However, we don't consider this inconsistent with the nDDR model described by the McStay Lab. They show RAD51 can localize to nucleolar caps outside S phase, and proposed it is related to the specialized repair environment at damaged rDNA, where the repetitive nature of the locus may permit homology-directed repair independently of an actively replicating sister chromatid. In our system, RAD51 recruitment is clearly much more prominent in EdU+ cells, which is biologically consistent with RAD51 acting on resected ssDNA intermediates that are expected to be more abundant in S/G2 and in replication-associated repair contexts.

We acknowledge that in Fig 3C, RAD51 recruitment to caps in EdU-negative GRASP cells is not detected. We do not interpret this as RAD51 cannot be recruited outside S phase, but rather that recruitment in non-replicating cells is less frequent and/or weaker, and therefore more difficult to detect in this assay.

This distinction is also consistent with our interpretation that RAD54L has a broader role at damaged rDNA than RAD51, which engaged in both RAD51-associated HR repair in S-phase cells and RAD51-independent replication fork stabilization, and lesion processing functions that are not restricted to S phase.

We have revised the text to clarify that nucleolar cap formation defines a repair-permissive compartment following rDNA damage, but the recruitment of individual repair factors is dictated by their function.

• "RAD54L is a key HR translocase that regulates RAD51 filament dynamics." If no RAD51 foci are detected in EdU-negative cells, how do the authors explain RAD54L's effects in those cells? Clarification of the proposed mechanism outside S phase is needed.

We thank the reviewer for this point. We agree that RAD51 recruitment is not restricted to EdU-positive cells.

However, we do not consider the low levels of RAD51 foci in EdU-negative cells to preclude a functional role for RAD54L in these cells. RAD54L is a multifunctional ATP-dependent DNA translocase, and its activity is not limited to RAD51 filament regulation.

In S-phase cells, RAD54L acts together with RAD51 to promote HR intermediate resolution. In contrast, in EdU-negative cells, our data support a RAD51-independent role for RAD54L, likely involving chromatin remodelling and lesion processing at damaged rDNA. This is consistent with our observations that RAD54L loss impacts ATM-dependent signalling and nucleolar cap formation under CX-5461-induced stress, demonstrating that its function extends beyond canonical HR.

We therefore propose that RAD54L operates through distinct, context-dependent mechanisms: a RAD51-dependent role during S phase, and a RAD51-independent role in organising and processing damaged rDNA outside S phase.

We have revised the text to clarify this distinction and to avoid implying that RAD54L function is exclusively mediated through RAD51.

• "To test whether RAD54L loss alters RAD51 kinetics, we quantified RAD51 foci after ionizing irradiation (IR) ". Why switch to global nuclear IR-induced damage here, rather than staying within the rDNA-focused system? Please justify the model shift or provide complementary data in the nucleolar context.

We thank the reviewer for this important comment. We used IR as a complementary, well-established system to test whether the increased RAD51 signal in RAD54L-deficient cells reflects a general defect in RAD51 filament turnover, rather than a nucleolus-specific phenomenon. RAD54L is a DNA translocase with a defined biochemical role in promoting RAD51 filament disassembly, and IR provides a robust and temporally controlled readout of RAD51 assembly and resolution at canonical DSBs.

Importantly, the IR experiments were not intended to model nucleolar damage per se, but to establish the underlying mechanism- that RAD54L limits RAD51 persistence at dsDNA. This allows us to interpret the nucleolar phenotype within a broader mechanistic framework.

Crucially, we also provide direct evidence in the nucleolar context. In Fig 3C-D, siRNA-mediated depletion of RAD54L in the GRASP rDNA-DSB system leads to increased RAD51 foci at nucleolar caps following rDNA break induction, demonstrating that defective RAD51 resolution occurs specifically at damaged rDNA.

Thus, the IR and GRASP systems are complementary rather than interchangeable. The IR time course establishes a general role for RAD54L in promoting RAD51 filament turnover, while the GRASP experiments demonstrate that this mechanism operates within the nucleolar/rDNA damage context.

We have revised the text to clarify this rationale.

• "DNA fiber assays revealed that CX-5461 impairs replication fork progression (Fig 3G) supporting a model in which RAD54L is required to resolve replication-associated stress.". The inference feels ahead of the data. It would be more convincing to include fiber assays with RAD54L depletion alone, then combine RAD54L depletion with CX-5461. This is especially relevant given the additive effects observed in the micronucleus assay and the S-phase arrest.

In Fig. 5A-B, we included DNA fibre assays in RAD54L-deficient cells under basal conditions. These data show that RAD54L knockout alone results in increased fork speed and reduced fork stability, indicating impaired fork restraint even in the absence of exogenous stress. This directly supports a role for RAD54L in replication fork stabilisation, consistent with a prior report (Uhrig et al., NAR 2024).

We then integrated these findings with our CX-5461 response data. RAD54L-deficient cells exhibit enhanced replication-associated stress (increased nucleolar pRPA(S33) (Fig 5C-D)), increased UBTF-positive micronuclei, and accumulation in S-phase/>4N populations following CX-5461 treatment (Fig 3F-I).

Importantly, our conclusion is not based on CX-5461 fibre data alone. Rather, it is supported by two complementary observations:
(i) RAD54L loss intrinsically destabilises replication forks, and
(ii) CX-5461 further exacerbates replication-associated stress in this context.

We agree that a direct side-by-side fibre analysis combining RAD54L depletion with acute CX-5461 treatment would provide additional resolution. However, the inclusion of basal fibre assays, together with orthogonal markers of replication stress (pRPA(S33), micronuclei, and cell-cycle effects), provides convergent evidence supporting the model that RAD54L functions in fork restraint/stabilisation and is required to limit replication-associated stress at rDNA.

We have revised the text to clarify this logic and to avoid overstating conclusions based solely on CX-5461-treated conditions.

• Fig. 4D: The difference is not convincing. At low dose, KO1 (the less efficient knockout) shows a stronger effect than the full KO. How do the authors explain this? Also, how were these events scored, and are they truly nucleolar caps? The morphology does not clearly support cap formation and since translacotion is preceeding induction of gH2AX, this may be part of the phenotype.

We thank the reviewer for this careful comment. We respectfully do not interpret Fig. 4D as showing that KO1 has a stronger phenotype than the more complete knockout. At the low dose of CX-5461 (100 nM), both RAD54L-deficient clones show minor quantitative variation that falls within expected biological and scoring variability. This condition represents a low-signal regime, and differences at this level are not biologically meaningful when considered in isolation.

Importantly, 100 nM CX-5461 induces a submaximal nucleolar stress state in which ATM-dependent nucleolar reorganisation is only partially engaged. In this context, γH2AX alone is not a robust standalone readout. Accordingly, γH2AX was not scored as diffuse nuclear signal, but specifically within UBTF-defined nucleolar cap regions. Caps were defined by redistribution of UBTF into peripheral cap-like structures (≤5 foci per nucleolus), with nucleolar regions further delineated using nucleolar markers and DAPI exclusion.

We agree that cap morphology is less pronounced at low-dose CX-5461, and therefore γH2AX alone may not fully capture cap formation under these conditions. For this reason, we interpret Fig. 4D alongside complementary readouts. At 100 nM CX-5461, RAD54L-deficient cells show reduced UBTF-defined cap formation (Fig. 4E), resistance to Pol I repression (Fig. 4F), and increased pRPA(S33) (Fig. 5C), despite no increase in γH2AX. These findings are consistent across both clones and support a model in which RAD54L loss attenuates ATM-dependent nucleolar reorganisation rather than increasing total DNA damage at this stage.

At higher dose (1 μM CX-5461), the phenotype becomes more pronounced, with increased γH2AX at nucleolar caps reflecting persistent damage. Together, these data indicate that RAD54L facilitates the processing of replication-associated lesions into ATM-activating structures that drive organised nucleolar cap formation. In its absence, lesions are instead biased toward ssDNA-rich intermediates, consistent with increased pRPA(S33), fork instability, and elevated nucleolar R-loops, shifting signalling toward ATR/DNA-PK-dominant responses.

We have revised the text to clarify that the low-dose phenotype is interpreted in the context of these integrated readouts, rather than γH2AX quantification alone, and that RAD54L primarily promotes the conversion of replication stress into an organised ATM-dependent nucleolar damage response.

• Fig. 4F: “RAD54L KO1 reduced Pol I transcription in the absence of exogenous damage, indicating a basal role in nucleoli.” This again supports a basal role of RAD54L in nucleolar function but the authors ignore this.

We thank the reviewer for this insightful comment. We agree that the reduction in Pol I transcription in RAD54L-deficient cells under basal conditions supports a constitutive role for RAD54L in nucleolar function, and we acknowledge that this point was not sufficiently emphasised in the original manuscript.

Importantly, we interpret this basal phenotype as fully consistent with our overall model rather than as a separate observation. RAD54L-deficient cells exhibit increased basal replication-associated stress at rDNA, including elevated R-loops and ssDNA, which would be expected to compromise efficient rRNA synthesis. Thus, the reduction in Pol I transcription provides functional evidence of disrupted nucleolar homeostasis in the absence of RAD54L.

We have revised the manuscript to explicitly integrate this finding and to clarify that RAD54L has both basal and damage-induced roles in the nucleolus. Under unstressed conditions, RAD54L supports replication fork restraint and rDNA integrity, thereby maintaining optimal Pol I transcription. Following nucleolar stress or rDNA damage, RAD54L is further recruited to nucleolar caps, where it contributes to the execution of the nDDR.

• “These findings indicate that RAD54L promotes fork reversal and stabilization, whereas RAD51C primarily contributes to fork protection under replication stress.” The metric labeled “fork symmetry” is not measured from a bidirectional origin and not from a CldU/IdU ratio, which may primarily reflects fork progression, not symmetry. Termination during the second pulse would appear “asymmetric” without necessarily indicating instability. Measuring this ratio under stress, as done with HU, is appropriate for fork protection. Why is HU used in this context when the study focuses on CX-5461?

We thank the reviewer for this thoughtful comment. We believe there is a misunderstanding regarding the use of HU in our study. HU was not used to assess “fork symmetry,” but rather to measure replication fork stability/protection in RAD54L-deficient cells under controlled replication stress conditions.
Specifically, the CldU/IdU tract ratio in the HU experiment reflects nascent strand degradation and fork protection, a well-established approach in the field. In this context, shortening of the second label is indicative of fork degradation die to lack of protection rather than termination-related artefacts. We agree that, in the absence of stress, this metric can reflect fork progression dynamics; however, under HU treatment, where forks are stalled, it is widely used as a readout of fork protection.

We also agree that the term “fork symmetry” is not precise for our basal fibre assay, and we have revised the text to clarify that this measurement reflects fork progression continuity/stability rather than true bidirectional symmetry from a shared origin.

Finally, HU was used deliberately to assess fork stability in a canonical system, whereas CX-5461 was used to model nucleolar, rDNA-associated replication stress central to this study.
We have revised the manuscript to clarify these distinctions and to avoid conflating fork progression, symmetry, and protection.

• Fig. 5C: Why is the count labeled “at nucleolar caps”? Vehicle samples should not display caps, and depletion reduces cap frequency. The text elsewhere refers to nucleoli without specifying caps. Please align labeling with the actual structures scored.

We thank the reviewer for this careful comment and appreciate the opportunity to clarify this point. In Fig. 5C, we quantified pRPA(S33) signal specifically within nucleolar cap regions, rather than across the entire nucleolus.

Caps were defined based on UBTF redistribution into peripheral cap-like structures, consistent with established morphological criteria. We agree that under basal conditions nucleolar caps are rare; however, a small proportion of untreated cells do exhibit cap-like structures, reflecting cellular heterogeneity in nucleolar organisation. These were included in the analysis as they met the predefined morphological criteria.

Our intention in Fig. 5C was to assess damage-associated pRPA(S33) accumulation specifically at reorganised nucleolar structures, rather than general nucleolar signal.
We have revised the main text to clarify that caps were explicitly scored based on UBTF-defined morphology.

• The use of DRB as an RNA Pol II inhibitor: The IF in Fig. 4E shows strong inhibition of nucleolar transcription, consistent with prior work indicating that Pol II inhibition also impacts Pol I. Conclusions that treat DRB as Pol II-specific in this context risk being misleading and should acknowledge the Pol I effects.

We thank the reviewer for this important comment. We agree that prolonged inhibition of RNA polymerase II can indirectly impact Pol I transcription.

However, in our experiments DRB treatment was limited to 4 hours, a duration widely used to achieve acute inhibition of Pol II transcription. Under these conditions, the primary effect of DRB is expected to be on Pol II activity, with indirect effects on Pol I transcription being comparatively limited.

Importantly, our conclusions do not depend on DRB being strictly Pol II-specific. Rather, DRB is used as a tool to acutely suppress Pol II-dependent transcription, allowing us to test its contribution to nucleolar R-loop formation and the associated DNA damage response. The reduction in nucleolar signal is therefore interpreted in this functional context. We have revised the manuscript to clarify that DRB is used here as an acute Pol II inhibitor.

• Fig. 6A: The manuscript states that R-loops are reduced upon rDNA DSBs. However, measuring at the cap stage is too late, since R-loops would be resolved by then. One would expect early R-loop increases before nucleolar restructuring and prior to Pol I silencing. The current design does not capture that window.

We thank the reviewer for this important point. We agree that R-loop dynamics are likely to be temporally regulated following rDNA DSB induction, and that early increases may precede nucleolar restructuring and cap formation.

In the current study, our measurements were performed at the nucleolar cap stage, which reflects a later time point in the nDDR. As the reviewer notes, this stage likely captures a phase where R-loops are already being processed as part of the repair response.

While we do not directly capture the earliest time window of R-loop accumulation prior to cap formation, our conclusions are based on comparative analyses across conditions at the same stage, allowing us to assess how transcriptional perturbations influence R-loop levels and the nDDR

Importantly, DRB was applied acutely (4 hours) and the more pronounced reduction in R-loop signal following DRB treatment, compared to the Pol I inhibitor ActD, supports a contribution from Pol II-associated transcription to nucleolar R-loop formation upon rDNA DSBs. We have clarified this point in the manuscript.

• Fig. 6D: What is PMTR? Please define the acronym on first use.

PMTR is acronym for promoter. This has been revised.

• Fig. 6D, GRASP1 cells: Does this indicate the presence of both gRNAs? It appears only one gRNA is used. In the rebuttal the authors state they now provide both, but the supplement still shows U2OS with the same single gRNA. Please clarify and provide data if both gRNAs were used.

We thank the reviewer for raising this point. In both OVCAR8-Cas9 and U2OS-Cas9 cells, the GRASP models were generated by transduction with a dual-sgRNA cassette cloned into the pCLIP-dual-SFFV-Puromycin vector, which encodes both sgRNA1 and sgRNA2 targeting rDNA (sequences listed in Table S3), followed by single-cell cloning. Thus, the OVCAR8 and U2OS GRASP clones were generated using the same dual-guide design, not a single-guide construct. This is stated explicitly in the revised manuscript.

• The R-loop results are not clearly integrated with RAD54L's role. In the model, the R-loop pathway appears separate. Please articulate how RAD54L mechanistically interfaces with R-loop dynamics at rDNA.

We thank the reviewer for this important comment. We agree that the connection between RAD54L and R-loop dynamics required clearer integration, and we have revised both the text and model to reflect a unified mechanism.

Our data support a unified framework in which RAD54L interfaces with R-loop dynamics at rDNA by regulating the replication context that determines their formation and persistence. Rather than acting directly on R-loops, RAD54L limits replication stress and fork instability, thereby constraining the conditions that promote pathological R-loop accumulation.

Under basal conditions, nucleolar R-loops arise from both Pol I and Pol II transcription (Fig. 6A-B). Loss of RAD54L leads to increased fork speed, elevated pRPA(S33), and accumulation of nucleolar R-loops, indicating increased ssDNA exposure and transcription–replication conflicts at rDNA. In this setting, R-loops are best interpreted as a downstream consequence of impaired fork stability.

Under nucleolar stress, however, the R-loop landscape shifts and becomes predominantly Pol II–driven. Following rDNA DSB induction, Pol II inhibition (DRB) more effectively suppresses R-loops than Pol I inhibition (ActD), accompanied by increased Pol II occupancy and antisense intergenic transcription at rDNA. These Pol II–dependent R-loops are associated with nucleolar reorganisation and recruitment of DNA repair factors (Fig. 7A–D), suggesting a functional role in the nucleolar DDR.

Importantly, CX-5461 increases nucleolar R-loops in parental cells but does not further elevate them in RAD54L-deficient cells, indicating that RAD54L loss and nucleolar stress converge on a shared pathway that promotes R-loop accumulation.
Together, these findings place RAD54L upstream of R-loop dynamics by controlling replication fork stability: RAD54L suppresses pathological R-loop accumulation under basal conditions by limiting transcription–replication conflicts, while under stress, Pol II–driven R-loops emerge as a dominant, DDR-associated species. We have revised the manuscript to explicitly reflect this mechanistic integration in both the text and model.

Discussion: "CX-5461–mediated replication stress was exacerbated by RAD54L loss, linking RAD54L directly to replication stress tolerance at rDNA.". The data, as presented, do not yet establish rDNA specificity, neither for CX-5461 nor for RAD54L. Additional controls would be needed to support that link.

We thank the reviewer for this important point. We agree that our data do not formally demonstrate exclusive rDNA specificity for either CX-5461–induced replication stress or RAD54L function.

However, our conclusions are supported by multiple independent, nucleolar-focused readouts, including RAD54L localisation to the nucleoli and UBTF-defined nucleolar caps, and assessments of nucleolar pRPA(S33), nucleolar γH2AX in RAD54L KO cells, all of which consistently indicate that the replication stress phenotype is strongly enriched at rDNA in RAD54L KO cells. In addition, CX-5461 is widely characterised as a Pol I

inhibitor that preferentially perturbs rDNA transcription and induces replication stress at these loci, providing a well-established biological framework for our observations.

We acknowledge that we cannot exclude contributions from other genomic regions, and we have revised the text to avoid implying strict specificity.

We have also clarified that RAD54L likely contributes more broadly to replication stress tolerance genome-wide, while our data highlight a particularly prominent role at rDNA, consistent with the high transcriptional and replicative burden of this locus.

Reviewer #2 (Remarks to the Author):

Authors have attended my suggestions and the manuscript is much stronger now.

We sincerely thank Reviewer 2 for their acknowledgment of the quality of our work.

Underlined text reflects **new** comments (2nd round review) by Reviewer 3 "**Reviewer comment2"** and new responses are in blue

Reviewer #3 (Remarks to the Author):

Reviewer #3

We thank the reviewer for the careful evaluation of our manuscript and for raising several important conceptual and technical points. In response to these comments, we have substantially revised the manuscript to clarify the mechanistic conclusions and to focus the study on the functional role of RAD54L in the nucleolar DDR. Importantly, the revised manuscript no longer presents the study as a discovery-based proteomic analysis. Instead, we have removed the genome-wide discovery datasets (including aniPOND proteomics and the CRISPR morphology screen) and refocused the manuscript on mechanistic experiments examining RAD54L function during rDNA damage and nucleolar replication stress. This restructuring addresses several of the concerns raised by the reviewer regarding interpretation of the proteomic datasets and strengthens the conceptual clarity of the work. We have also revised the Discussion to more clearly define the mechanistic insights provided by the study and to better place our findings within the context of HR and rDNA maintenance. Our detailed responses to each comment are provided below.

Reviewer comment2: **The revised manuscript has been significantly improved** and now more clearly presents the finding that RAD54L is recruited to nucleolar caps, where rDNA repair occurs, and that upregulation of IGS-derived RNA promotes nucleolar cap formation. Although the precise mechanisms remain to be fully elucidated, these

findings provide important insights into the field. While the study is of clear interest, **further clarification about minor points** below would strengthen the manuscript and enhance its suitability for publication.

We thank the reviewer for their positive and encouraging assessment of our revised manuscript. We agree that, while the precise purpose of IGS-derived R-loops require further investigation, our findings in this manuscript provide important insights into nucleolar DNA damage responses and rDNA repair.

We also appreciate the reviewer's constructive suggestions. We have carefully addressed each of the minor points raised below and have revised the manuscript accordingly to improve clarity and strengthen the presentation.

Reviewer comment1: "There is no clear evidence that R-loop formation plays a functional role in DSB repair... R loops at the IGS induce collisions with replication forks generating additional DSBs... This makes it difficult to fully appreciate the conceptual advances in nDDR presented in the study."

Response: We thank the reviewer for this important conceptual point. We agree that our data do not demonstrate that R-loops play a functional role in DNA repair. Accordingly, we have revised the manuscript to clarify this interpretation. In the revised text, we now present R-loop formation as a transcription-associated feature of the nucleolar DDR, rather than a direct driver of repair. Our DRIP-qPCR and Pol II ChIP-qPCR experiments demonstrate that rDNA damage is accompanied by Pol II recruitment and transcriptional activity at intergenic rDNA regions, which correlates with the formation of R-loop structures. However, we now state more cautiously that these structures likely reflect the transcriptionally dynamic environment accompanying nucleolar DDR and nucleolar re-organization rather than a direct mechanism of DSB repair. Importantly, the central conceptual advance of the revised manuscript is the identification of RAD54L as a critical nucleolar DDR factor. We show that RAD54L is recruited to nucleolar caps following rDNA damage and that its loss leads to persistent RAD51 accumulation, replication fork destabilization, elevated γH2AX, and increased rDNA-associated genomic instability. These findings support a model in which RAD54L remodels replication forks during nucleolar replication stress and also acts downstream of RAD51 to promote efficient completion of HR at damaged rDNA arrays.

Reviewer comment2: Could the authors elaborate on the possible reasons why RAD54L functions as a critical factor for the repair of rDNA repeats following DSB induction and/or replication stress? For example, this may be related to the unique repetitive structure of rDNA.

We thank the reviewer for this insightful comment. We agree that the unique structural and functional features of rDNA repeats are likely to underlie the prominent requirement for RAD54L in this context, and we have expanded the Discussion accordingly.

rDNA loci present a distinct genomic environment characterised by high transcriptional output, repetitive sequence organisation, enriched in G4 DNA structures and transcription–replication conflicts. These features increase the likelihood of replication fork stalling, ssDNA exposure, and R-loop formation, all of which create a challenging substrate for genome maintenance.

The repetitive nature of rDNA also imposes additional constraints on DNA repair pathway choice. While homology-directed repair is facilitated by the availability of repeated sequences, it must be tightly regulated to prevent aberrant recombination events and maintain repeat integrity. In this setting, factors such as RAD54L, which regulate RAD51 filament dynamics and DNA strand exchange, are likely to play an important role in ensuring controlled and productive repair.

In addition, our data support a model in which RAD54L contributes to replication fork restraint and stability, thereby limiting the accumulation of replication-associated intermediates (e.g., ssDNA and R-loops) that can both promote damage and complicate repair. Loss of RAD54L therefore creates a permissive environment for transcription-replication conflicts, increasing the burden on repair pathways at these loci.

We therefore propose that RAD54L is particularly important at rDNA because it integrates both replication-associated stress control and DNA repair functions within a highly transcribed, repetitive genomic context.

We have revised the Discussion to explicitly incorporate these points and to highlight how the unique features of rDNA contribute to the dependence on RAD54L.

Specific comments Reviewer comment: “Please clarify the evidence supporting the claim that S-phase delay occurs following the induction of DSBs”

Response: We thank the reviewer for highlighting this point. As the primary focus of this study is the early dynamics of the nucleolar DDR within the first 24 hours following rDNA damage, we have removed the cell-cycle analysis of GRASP cells from the revised manuscript to maintain a clearer focus on the central findings. Instead, we expanded the functional analysis of RAD54L in rDNA repair. In the revised manuscript, we included additional experiments measuring micronuclei formation following Cytochalasin B treatment in GRASP cells with RAD54L knockdown (Fig 3E). RAD54Ldeficient GRASP cells displayed a significantly higher frequency of micronuclei following rDNA DSB induction, including rDNA-containing micronuclei marked by UBTF staining (Fig 3E). These findings suggest that loss of RAD54L compromises rDNA repair and results in chromosome segregation defects that lead to micronucleation and genomic instability.

Reviewer comment2: I appreciated the micronuclei accumulation probably containing rDNA by RAD54 KD, which suggesting a new feature of rDNA instability.

We thank the reviewer for this positive comment. We agree that the accumulation of micronuclei containing rDNA in RAD54L-deficient cells likely reflects increased genomic instability and contributions from rDNA loci.

Reviewer comment: AniPOND-MS and EdU incorporation at repair sites. “When DSBs are induced on rDNA array, EdU are also incorporated at DSB sites .... I am concern about the possibility that mass spectrometry data does not necessarily contain the proteins associated with the replication forks.”

Response: We appreciate the reviewer highlighting this important technical limitation. In response, we have removed the aniPOND-MS dataset from the revised manuscript. While these experiments were originally included as exploratory analyses, we agree that distinguishing proteins associated with replication forks from those associated with repair synthesis is challenging in this context. The revised manuscript now focuses on mechanistic experiments addressing RAD54L function in nucleolar DDR.

Reviewer comment2: OK

Reviewer comment: “R-loops can form without DSB induction to regulate Pol I transcription. Do these collide with replication forks without DSB induction?”

Response: We thank the reviewer for raising this point. We have clarified this in the revised manuscript. Basal R-loops are indeed present at rDNA loci, under normal conditions, and have been reported to be associated with Pol II transcriptional activity at intergenic spacer regions (Abraham et al 2020 Nature). Under these baseline conditions, R-loops are also generated cotranscriptionally at the 47S rRNA coding region by Pol I (Fig 6D). R-loops are known to have the potential to interfere with replication fork progression thereby contributing to transcription-replication conflicts. However, in our study we did not directly test whether basal rDNA R-loops lead to replication fork collisions in the absence of induced DNA damage. Instead, our data show that rDNA damage is associated with an increase in Pol II-mediated R-loops, consistent with elevated Pol II binding and transcriptional activity at intergenic regions during nDDR(Fig 6D-G and Fig S5C-D). Accordingly, in the revised manuscript we have moderated our interpretation and now describe these R-loops as transcription-associated structures that accompany nucleolar DDR, rather than concluding that they directly drive replication-fork collisions in our system.

Reviewer comment2:OK

Reviewer comment: Provide quantitative analysis of FBL fragmentation in Fig 4A-B.

Response: We thank the reviewer for this suggestion. In the revised manuscript, the nucleolar morphology CRISPR screen and associated analyses of FBL fragmentation have been removed as part of the restructuring of the study. This change allowed us to focus the manuscript more clearly on the functional role of RAD54L in nucleolar DDR.

Reviewer comment2: OK

Reviewer comment: “I wonder if the R-loops is different from the R-loops observed after DSB induction in both GARSP1 and GRASP2 cells of Figure 5.”

Response: We thank the reviewer for this important observation. We agree that basal R-loops detected in untreated cells likely arise from Pol I transcription within the rDNA coding region and from Pol II transcription within intergenic spacer (IGS) regions. This is supported by our DRIPqPCR analysis (Fig 6D), which detects R-loop signal under basal conditions. Consistent with this interpretation, treatment with both the Pol II inhibitor DRB and the Pol I inhibitor Actinomycin D reduced R-loop signal in OVCAR8 cells (Fig 6A-B), indicating that nucleolar R-loops can arise from transcriptional activity driven by both polymerases. In contrast, our DRIP-qPCR experiments following rDNA damage show increased R-loop signal specifically at the promoter region and intergenic rDNA regions adjacent to the DSBs (Fig 6D). Furthermore, immunofluorescence analysis of R-loops revealed that Pol II inhibition with DRB significantly reduced R-loop signal following rDNA damage compared to Actinomycin D treated cells (Fig 6A-B), consistent with Pol II-transcription-associated R loops upon rDNA DSBs. Based on these findings, we have clarified in the revised manuscript that basal R-loops associated with Pol I transcription differ from the damage-associated R-loops that arise following rDNA DSB induction, likely reflecting Pol II-dependent transcriptional activity during the nucleolar DDR.

Reviewer comment2: In revised Fig. 6D, The DRIP–qPCR analysis using the S9.6 antibody shows only a modest difference in R-loop formation in IGS regions following DSB induction. However, given the known limitations in the specificity of the S9.6 antibody, this difference does not appear to be substantial. As shown in Fig. S5B, the authors suggest that the DRIP signal detected at some genes is attributable to R-loops, based on its reduction upon RNase H treatment. It would be helpful if the authors could examine whether the S9.6 antibody specifically detects R-loops in IGS region of rDNA.

We thank the reviewer for this important comment. We agree that the specificity of the S9.6 antibody is a known limitation and that DRIP-based measurements should be interpreted with appropriate caution.
To address this, we have included RNase H treatment controls (Fig S5B), which demonstrate that the DRIP signal is sensitive to RNase H digestion, supporting the conclusion that the signal detected reflects RNA-DNA hybrids.
With respect to the rDNA IGS regions, we acknowledge that the magnitude of change detected by DRIP-qPCR following DSB induction is modest. This likely reflects both the transient and dynamic nature of R-loop formation at these loci and technical limitations in resolving changes within highly repetitive rDNA sequences.
While we have not performed locus-specific RNase H validation exclusively at IGS regions, the global RNase H sensitivity of the DRIP signal, supports the interpretation that the detected signal represents bona fide R-loops. Nevertheless, we can address this.

Reviewer comment: “Could the decrease in TP53BP1 foci be due to reduced TP53BP1

expression?”

Response: We thank the reviewer for this suggestion. In the revised manuscript, we confirmed by western blotting that DRB treatment does not reduce TP53BP1 protein levels under the conditions used in our experiments (Fig S6C). Therefore, the reduction in TP53BP1 nucleolar foci following Pol II inhibition is unlikely to result from decreased TP53BP1 expression. Instead, these results support the idea that Pol II transcription contributes to the nucleolar DDR that facilitates the formation of nucleolar caps and subsequent TP53BP1 and RAD54L localization at damaged rDNA loci.

Reviewer comment2: OK

Reviewer comment: Western blot images appear combined.

Response: We thank the reviewer for noting this issue. The blot was cropped to remove an empty lane, and this was not intended to obscure any data. In the revised manuscript, we now provide an updated blot of RAD54L in OVCAR8 and RAD54L KO cell lines (Fig 1B).

Reviewer comment2: In the western blot shown in the revised Fig. 1B, RAD54L knockout1 still exhibits a weak RAD54L band, whereas knockout 2 shows no detectable signal. Could the authors clarify which regions of RAD54L are deleted in each knockout cell line, respectively?

We thank the reviewer for this important point. The residual RAD54L signal observed in KO1, compared to the complete loss in KO2, likely reflects differences in editing outcomes between the two clones.
Both clones were generated using CRISPR/Cas9 with four synthetic guide RNAs (Table S2), and the difference in protein expression likely arises from distinct indel events affecting each allele in the respective clones. Based on the protein expression data, we interpret KO2 as a complete (biallelic) knockout, whereas KO1 is most consistent with a partial or heterozygous disruption, resulting in low residual RAD54L protein levels.
Importantly, the phenotypic differences observed between KO1 and KO2 across multiple assays are consistent with the degree of RAD54L depletion, supporting this interpretation.
We have revised the manuscript to clarify that KO2 represents a complete knockout, while KO1 retains low residual RAD54L expression.

Reviewer comment: In Figure 6D, the colony formation assay shows the clear difference. But the corresponding bar graph in figure 6E for the quantitative analysis represents little difference. Could authors clarify the discrepancy?”

Response: We thank the reviewer for their careful assessment of the data. During revision we identified that RAD54L KO1 and KO2 cell lines were inadvertently mislabelled in the representative colony formation images, which contributed to the apparent discrepancy between the images and the quantification. This has now been corrected in the revised manuscript. In the revised manuscript, the cell lines are correctly labelled with KO1 representing a partial RAD54L knockout and KO2 representing a complete

RAD54L knockout. The proliferation data (Fig 1C) and colony formation assays (Fig 1D) show that sensitivity to CX-5461 correlates with the extent of RAD54L depletion, with the stronger knockout exhibiting increased sensitivity.

Reviewer comment2: What does a partial RAD54L knockout mean?

We thank the reviewer for this point. By "partial RAD54L knockout," we refer to a clone in which RAD54L is not fully ablated at the protein level, resulting in residual expression. In our study, KO1 displays a low but detectable RAD54L signal by Western blot, whereas KO2 shows complete loss of detectable protein. This difference most likely reflects distinct CRISPR/Cas9 editing outcomes, where KO2 represents a biallelic loss-of-function, while KO1 retains at least one allele that produces a partially functional protein. We have revised the manuscript to clarify this point.

Reviewer comment: "I am not fully convinced of the recruitment of RAD54L to the nucleoli based on the foci in Figure 6I, although the intensity of RAD54L appears to be higher in GRASP cells compared to control cells. Could the authors provide further clarification by the quantitative analysis?"

Response: We thank the reviewer for this helpful suggestion. In the revised manuscript, we performed additional experiments using an independent RAD54L antibody that produces lower background staining, allowing clearer visualisation of nucleolar RAD54L accumulation. We now provide representative single-cell images with magnified views of nucleoli to more clearly demonstrate RAD54L localization at nucleolar caps following rDNA damage in GRASP OVCAR8 and U2OS cell models (Fig 2A-B, Fig S1B; Fig 2I-J and Fig. S2I). Regarding the quantification previously shown in Fig. 6H, in the revised manuscript, nucleolar RAD54L localization is manually quantified as described in the Methods by scoring RAD54L foci within nucleolar cap regions. Quantification of the total number of RAD54L foci at nucleolar caps per cell provided a more informative measure of the magnitude of RAD54L recruitment to rDNA damage sites. This updated analysis confirms the same trend observed with the threshold-based scoring and is now included in the revised figure. Together with the magnified representative images and improved antibody specificity, these data more clearly demonstrate RAD54L accumulation at nucleolar caps following rDNA damage.

Reviewer comment2: OK

Dr. Elaine Sanij
St Vincent's Institute of Medical Research
DNA Damage and Cancer Therapy Laboratory
9 Princes St
Melbourne, Victoria 3065
Australia

21st May 2026

Re: EMBOJ-2026-124510-T
RAD54L coordinates the nucleolar DNA damage response to maintain rDNA stability

Dear Elaine,

Thank you again for transferring your previously reviewed and revised manuscript to The EMBO Journal. As discussed earlier, we sent it to one arbitrating referee with expertise in nucleolar chromatin/transcription and rDNA integrity, to particularly assess your responses to the remaining issues with these aspects raised by original referee 1. I am pleased to say that our arbitrator considered the data in the manuscript sufficiently strong to support most key conclusions of the work, and also felt that the remaining referee comments had been adequately addressed at this stage. We shall therefore be happy to proceed with formal acceptance and publication of this work in The EMBO Journal, as soon as the following editorial points are incorporated into a final version of the manuscript (please also consider our more detailed online Guide to Authors):

GENERAL:

- Please download and complete our author checklist (link provided below).

- Please upload the manuscript text (including figure legends) as an editable text file, and all main figures without legends as individual image files with sufficient resolution/quality for production.

- Please provide suggestions for a short 'blurb' text prefacing and summing up the conceptual aspect of the study in two sentences (max. 250 characters), followed by 3-5 one-sentence 'bullet points' with brief factual statements of key results of the paper; they will form the basis of an editor-written 'Synopsis' accompanying the online version of the article. Please also upload a synopsis image, which can be used as a "visual title" for the synopsis section of your paper (maybe utilizing Fig 7E for this purpose?)). The image should be ideally in JPG format, and please make sure that it remains in the modest dimensions of (exactly) 550 pixels wide and 300 pixels high.

TEXT:

- Please adjust the order as well as the headers of the different manuscript sections: Title page with complete author information, Abstract, Introduction, Results, Discussion, Methods, Data Availability, Acknowledgements, Disclosure and Competing Interests Statement, References, Main Figure Legends, Tables.

- As we are switching from a free-text author contribution statement towards a more formal statement based on Contributor Role Taxonomy (CRediT) terms, please remove the present Author Contribution section and instead specify each author's contribution(s) directly in the Author Information page of our submission system during upload of the final manuscript. See https://casrai.org/credit/ for more information.

- Please include a dedicated "Data Availability" section at the end of the Material and Methods (suggested wording: "The [structural coordinates | microarray | mass spectrometry] data from this publication have been deposited to the [name of the database] database [URL] and assigned the identifier [accession | permalink | hashtag].") This section should also mention any deposited code. Note that only datasets newly generated during the course of this study should be listed here; should there no data deposition to public repositories linked to the study, this should still be stated as "This study includes no data deposited in external repositories."

- Please adjust the format of the reference list and of the in-text citations according to EMBO Journal format (alphabetical order, author name et al + year, first up to 10 authors should be listed, followed by 'et al'), also check that all citations are complete with complete volume and page numbers (DOI info only in case of advance publications that do not yet have volume/page numbers).

- Please move the funding information into the Acknowledgement section, and make sure to also enter all relevant funding sources into our submission system. This may also need to include the paragraph on institute funding?

- As reagent table, please make sure to use the template available in our Guide to Authors (https://media.springernature.com/original/springer-cms/rest/v1/content/27825802/data/v1). Please consider which contents of the currently provided 'reagent table' could become part of the templated Reagents & Tools table, and which ones might be uploaded in separate files as Expanded View tables (callout: "Table EV1/2/3...") and referenced from the methods section/from the R&T table.

ll Materials and Methods need to be described in the main text using our 'Structured Methods' format. The Methods section should include a (separately uploaded) Reagents and Tools Table (downloadable at https://www.embopress.org/page/journal/14693178/authorguide#structuredmethods) listing key reagents, experimental models, software and relevant equipment, and including their sources and relevant identifiers; and a part describing the methods (ideally using a step-by-step protocol format to facilitate adoption of the methodologies across labs).

- Finally, it may be helpful to expand the introduction a bit, in particularly to give some more background on known functions and mechanisms of RAD54L.

DATA:

- Please refer to our author guide (www.embopress.org/page/journal/14602075/authorguide#expandedview) regarding "supplementary figures". Ideally, the PDF containing current figures S1-S6 should be renamed into Appendix, and prefaced with a title page containing a brief Table of Contents and the header "Appendix for [manuscript title]|". Make sure to rename these figures into "Appendix Figure S1-S6" on all occasions (in the Appendix & its ToC, when referencing them in the text...), and place each respective legends underneath each Appendix figure.

- During routine pre-acceptance checks, our data editors have raised the following queries regarding figures, data, and legends; I would appreciate if you briefly answered to them in the cover letter of your final submission, and made the requested text modifications with changes/additions highlighted via the "Track changes" option, to facilitate our final checking":
1. Please provide the missing information related to "n: in the legends of figures 1E, 3H, I; 4D, E; S2 B, H; S3 B
2. In figures 3E-F, it is not clear what the bar diagrams (mean/average, nature of error bars?) depict. Please note that should the values derived from only two individual values (as suggested by the figure legend?), then no statistical analysis would be justified and two individual data points should be plotted instead of error bars.
3. Please state the exact p values in the legends of figures 1D, E; 2B, E, F, G, H, J; 3B, C, G, H, I; 4D-F; 5A, B, C, F; 6B, D, E, F, G; 7B, C; S2 B, S3 B, S5 C, D; S6 B
4. Please define the white circles in the legend of figure 3A, D; 4C, 5D, 6A, 7A, D; S1B, S2 C, D, E, F, G, I; S6A.
5. Please define the white arrows in the legends of figures 3E, F.

- Finally, you shall also receive a separate message from our Source Data curation team, with instructions on how to prepare and upload relevant image and numerical raw data.

I am returning the manuscript to you now for the final revisions, with the hyperlink below allowing you to upload all files once you are ready. Should you need additional guidance/feedback regarding this final adjustments, please do not hesitate to contact us directly. Thank you again for the opportunity to consider this work for The EMBO Journal, and I look forward to receiving your final version!

With kind regards,

Hartmut

Hartmut Vodermaier, PhD
Senior Editor, The EMBO Journal
h.vodermaier@embojournal.org

*** PLEASE NOTE: All revised manuscript are subject to initial checks for completeness and adherence to our formatting guidelines. Revisions may be returned to the authors and delayed in their editorial re-evaluation if they fail to comply to the following requirements. As a first step please read our guidelines for revised submissions: https://link.springer.com/journal/44318/submission-guidelines#cms-Revised-submissions

1) Every manuscript requires a Data Availability section (even if only stating that no deposited datasets are included). Primary datasets or computer code produced in the current study have to be deposited in appropriate public repositories prior to resubmission, and reviewer access details provided in case that public access is not yet allowed.

2) Each figure legend must specify
- size of the scale bars that are mandatory for all micrograph panels

- the statistical test used to generate error bars and P-values
- the type error bars (e.g., S.E.M., S.D.)
- the number (n) and nature (biological or technical replicate) of independent experiments underlying each data point
- Figures may not include error bars for experiments with n<3; scatter plots showing individual data points should be used instead.

3) Revised manuscript text (including main tables, and figure legends for main and EV figures) has to be submitted as editable text file (e.g., .docx format). We encourage highlighting of changes (e.g., via text color) for the referees' reference.

4) Each main and each Expanded View (EV) figure should be uploaded as individual production-quality files (preferably in .eps, .tif, .jpg formats). For suggestions on figure preparation/layout, please refer to our Figure Preparation Guidelines:
https://media.springernature.com/original/springer-cms/rest/v1/content/27825798/data/v1

5) Point-by-point response letters should include the original referee comments in full together with your detailed responses to them (and to specific editor requests if applicable), and also be uploaded as editable (e.g., .docx) text files.

6) Please complete our Author Checklist, and make sure that information entered into the checklist is also reflected in the manuscript; the checklist will be available to readers as part of the Review Process File.

7) All authors listed as (co-)corresponding need to deposit, in their respective author profiles in our submission system, a unique ORCiD identifier linked to their name. Please see our Guide to Authors for detailed instructions.

8) Please note that supplementary information at EMBO Press has been superseded by the 'Expanded View' for inclusion of additional figures, tables, movies or datasets; with up to five EV Figures being typeset and directly accessible in the HTML version of the article.

9) To facilitate reproducibilty and cross-laboratory adoption of methodologies, please structure the Materials & Methods section as outlined in our guide to authors, including a completed Reagents and Tools Table.

10) Digital image enhancement is acceptable practice, as long as it accurately represents the original data and conforms to community standards. If a figure has been subjected to significant electronic manipulation, this must be clearly noted in the figure legend and/or the 'Materials and Methods' section. The editors reserve the right to request original versions of figures and the original images that were used to assemble the figure. Finally, we generally encourage uploading of numerical as well as gel/blot image source data.

At EMBO Press we ask authors to provide source data for the main manuscript figures. You will receive a separate email with instructions for providing source data with your revised manuscript, including how to upload and organize the files.

In the interest of ensuring the conceptual advance provided by the work, we recommend submitting a revision within 3 months (19th Aug 2026). Please discuss the revision progress ahead of this time with the editor if you require more time to complete the revisions. Use the link below to submit your revision:

Link Not Available

Referee #1:

This work by Liu and colleagues characterizes the interplay between rDNA transcription, rDNA damage responses, and RAD54L in cell lines. Overall, the data presented are comprehensive and well described. Perhaps due to the variety of measurements described, the resulting model is complex and multi-factorial. This complexity is unavoidable, given the complex interplay between DNA damage responses and cellular transcription programs. The connection between rDNA transcription (by RNA polymerases I and II) and R-loops remains cloudy, but that does not preclude the publication of these findings for motivating future studies.

In the eyes of this reviewer, the authors have sufficiently addressed previous comments raised by all reviewers and clarified the manuscript within the confines of the data.

EMBO Press Author Checklist

Corresponding Author Name: Elaine Sanij	
Journal Submitted to: The EMBO Journal	
Manuscript Number: EMBOJ-2026-124510	

USEFUL LINKS FOR COMPLETING THIS FORM
The EMBO Journal - Author Guidelines
EMBO Reports - Author Guidelines
Molecular Systems Biology - Author Guidelines
EMBO Molecular Medicine - Author Guidelines

Reporting Checklist for Life Science Articles (updated January
This checklist is adapted from Materials Design Analysis Reporting (MDAR) Checklist for Authors. MDAR establishes a minimum set of requirements in transparent reporting in the life sciences (see Statement of Task: 10.31222/osf.io/9sm4x). Please follow the journal's guidelines in preparing your
Please note that a copy of this checklist will be published alongside your article.

Abridged guidelines for figures
1. Data
The data shown in figures should satisfy the following conditions:
- the data were obtained and processed according to the field's best practice and are presented to reflect the results of the experiments in an accurate and unbiased manner.
- ideally, figure panels should include only measurements that are directly comparable to each other and obtained with the same assay.
- plots include clearly labeled error bars for independent experiments and sample sizes. Unless justified, error bars should not be shown for technical
- if n<5, the individual data points from each experiment should be plotted. Any statistical test employed should be justified.
- Source Data should be included to report the data underlying figures according to the guidelines set out in the authorship guidelines on Data

2. Captions
Each figure caption should contain the following information, for each panel where they are relevant:
- a specification of the experimental system investigated (eg cell line, species name).
- the assay(s) and method(s) used to carry out the reported observations and measurements.
- an explicit mention of the biological and chemical entity(ies) that are being measured.
- an explicit mention of the biological and chemical entity(ies) that are altered/varied/perturbed in a controlled manner.
- the exact sample size (n) for each experimental group/condition, given as a number, not a range;
- a description of the sample collection allowing the reader to understand whether the samples represent technical or biological replicates (including how many animals, litters, cultures, etc.).
- a statement of how many times the experiment shown was independently replicated in the laboratory.
- definitions of statistical methods and measures:
  - common tests, such as t-test (please specify whether paired vs. unpaired), simple χ2 tests, Wilcoxon and Mann-Whitney tests, can be unambiguously identified by name only, but more complex techniques should be described in the methods section;
  - are tests one-sided or two-sided?
  - are there adjustments for multiple comparisons?
  - exact statistical test results, e.g., P values = x but not P values < x;
  - definition of 'center values' as median or average;
  - definition of error bars as s.d. or s.e.m.

Please complete ALL of the questions below.
Select "Not Applicable" only when the requested information is not relevant for your study.

Materials

Newly Created Materials	Information included in the manuscript?	In which section is the information available? (Reagents and Tools Table, Materials and Methods, Figures, Data Availability Section)
New materials and reagents need to be available; do any restrictions apply?	Not Applicable	There are no restrictions on reagents' availability

Antibodies	Information included in the manuscript?	In which section is the information available? (Reagents and Tools Table, Materials and Methods, Figures, Data Availability Section)
For **antibodies** provide the following information:<br>- Commercial antibodies: RRID (if possible) or supplier name, catalogue number and or/clone number<br>- Non-commercial: RRID or citation	Yes	Reagents and Tools Table

DNA and RNA sequences	Information included in the manuscript?	In which section is the information available? (Reagents and Tools Table, Materials and Methods, Figures, Data Availability Section)
**Short novel DNA or RNA including primers, probes**: provide the sequences.	Yes	Table, EV2, Table EV3, Table EV4

Cell materials	Information included in the manuscript?	In which section is the information available? (Reagents and Tools Table, Materials and Methods, Figures, Data Availability Section)
**Cell lines:** Provide species information, strain. Provide accession number in repository **OR** supplier name, catalog number, clone number, and/**OR** RRID.	Yes	Reagents and Tools Table
**Primary cultures:** Provide species, strain, sex of origin, genetic modification status.	Not Applicable	
Report if the cell lines were recently **authenticated** (e.g., by STR profiling) and tested for mycoplasma contamination.	Yes	Reagents and Tools Table: Individuality and the identity of the cell lines were routinely confirmed by a polymerase chain reaction (PCR) based short tandem repeat (STR) analysis using six STR loci. No commonly misidentified cell lines were used in this manuscript.

Experimental animals	Information included in the manuscript?	In which section is the information available? (Reagents and Tools Table, Materials and Methods, Figures, Data Availability Section)
**Laboratory animals or Model organisms:** Provide species, strain, sex, age, genetic modification status. Provide accession number in repository **OR** supplier name, catalog number, clone number, **OR** RRID.	Not Applicable	
**Animal observed in or captured from the field:** Provide species, sex, and age where possible.	Not Applicable	
Please detail **housing and husbandry conditions**.	Not Applicable	

Plants and microbes	Information included in the manuscript?	In which section is the information available? (Reagents and Tools Table, Materials and Methods, Figures, Data Availability Section)
**Plants:** provide species and strain, ecotype and cultivar where relevant, unique accession number if available, and source (including location for collected wild specimens).	Not Applicable	
**Microbes:** provide species and strain, unique accession number if available, and source.	Not Applicable	

Human research participants	Information included in the manuscript?	In which section is the information available? (Reagents and Tools Table, Materials and Methods, Figures, Data Availability Section)
If collected and within the bounds of privacy constraints report on age, sex and gender or ethnicity for all study participants.	Not Applicable	

Core facilities	Information included in the manuscript?	In which section is the information available? (Reagents and Tools Table, Materials and Methods, Figures, Data Availability Section)
If your work benefited from core facilities, was their service mentioned in the acknowledgments section?	Not Applicable	

Design

Study protocol	Information included in the manuscript?	In which section is the information available? (Reagents and Tools Table, Materials and Methods, Figures, Data Availability Section)
If study protocol has been **pre-registered, provide DOI in the manuscript**. For clinical trials, provide the trial registration number **OR** cite DOI.	Not Applicable	

Report the **clinical trial registration number** (at ClinicalTrials.gov or equivalent), where applicable.	Not Applicable	

Laboratory protocol	Information included in the manuscript?	In which section is the information available? (Reagents and Tools Table, Materials and Methods, Figures, Data Availability Section)
Provide DOI OR other citation details if **external detailed step-by-step protocols** are available.	Not Applicable	

Experimental study design and statistics	Information included in the manuscript?	In which section is the information available? (Reagents and Tools Table, Materials and Methods, Figures, Data Availability Section)
Include a statement about **sample size** estimate even if no statistical methods were used.	Yes	We state in the Methods section that "no statistical method was used to predetermine the sample size, but we routinely, unless stated, employed at least three biological repeats for each experiment."
Were any steps taken to minimize the effects of subjective bias when allocating animals/samples to treatment (e.g. **randomization procedure**)? If yes, have they been described?	Yes	Randomization was not appropriate because samples were derived from specific genetic cell lines or treatment condition. This is stated in the methods section. Independent, biological replicate experiments were used to ensure reproducibility.
Include a statement about **blinding** even if no blinding was done.	Yes	We stated in the Methods section "The investigators were not blinded to allocation during the experiments and outcome assessment because all numerical data were software automated and independent of investigator subjectivity."
Describe **inclusion/exclusion criteria** if samples or animals were excluded from the analysis. Were the criteria pre-established?<br><br>If sample or data points were omitted from analysis, report if this was due to attrition or intentional exclusion and provide justification.	Yes	No data were excluded from any analyses. This is stated clearly in the methods section.
For every figure, are **statistical tests** justified as appropriate? Do the data meet the assumptions of the tests (e.g., normal distribution)? Describe any methods used to assess it. Is there an estimate of variation within each group of data? Is the variance similar between the groups that are being statistically compared?	Yes	All data presented in the manuscript are represented as the mean [plus or minus] SEM or SD and were analyzed using GraphPad Prism (version 10). The statistical test employed for each dataset has been specified in the figure legends.

Sample definition and in-laboratory replication	Information included in the manuscript?	In which section is the information available? (Reagents and Tools Table, Materials and Methods, Figures, Data Availability Section)
In the figure legends: state number of times the experiment was **replicated** in laboratory.	Yes	After the initial optimization of experimental conditions, we used biological replicates (not technical replicates) in this study to demonstrate statistical significance, following a defined methodology. The use of biological, independent replicates is clearly described in the manuscript text, relevant Methods sections, and Figure Legends.
In the figure legends: define whether data describe **technical or biological replicates**.	Yes	The use of biological replicates is clearly described in Figure legends.

Ethics

Ethics	Information included in the manuscript?	In which section is the information available? (Reagents and Tools Table, Materials and Methods, Figures, Data Availability Section)
Studies involving **human participants**: State details of **authority granting ethics approval** (IRB or equivalent committee(s), provide reference number for approval.	Not Applicable	
Studies involving **human participants**: Include a statement confirming that **informed consent** was obtained from all subjects and that the experiments conformed to the principles set out in the WMA Declaration of Helsinki and the Department of Health and Human Services Belmont Report.	Not Applicable	
Studies involving **human participants:** For publication of **patient photos**, include a statement confirming that consent to publish was obtained.	Not Applicable	
Studies involving experimental **animals**: State details of **authority granting ethics approval** (IRB or equivalent committee(s), provide reference number for approval. Include a statement of compliance with ethical regulations.	Not Applicable	
Studies involving **specimen and field samples:** State if relevant **permits** obtained, provide details of authority approving study; if none were required, explain why.	Not Applicable	

Dual Use Research of Concern (DURC)	Information included in the manuscript?	In which section is the information available? (Reagents and Tools Table, Materials and Methods, Figures, Data Availability Section)
Could your study fall under dual use research restrictions? Please check biosecurity documents and list of **select agents and toxins** (CDC): https://www.selectagents.gov/sat/list.htm	Not Applicable	
If you used a select agent, is the security level of the lab appropriate and reported in the manuscript?	Not Applicable	
If a study is subject to dual use research of concern regulations, is the name of the **authority granting approval and reference number** for the regulatory approval provided in the manuscript?	Not Applicable	

Reporting

The MDAR framework recommends adoption of discipline-specific guidelines, established and endorsed through community initiatives. Journals have their own policy about requiring specific guidelines and recommendations to complement MDAR.

Adherence to community standards	Information included in the manuscript?	In which section is the information available? (Reagents and Tools Table, Materials and Methods, Figures, Data Availability Section)
State if relevant guidelines or checklists (e.g., **ICMJE, MIBBI, ARRIVE, PRISMA**) have been followed or provided.	Not Applicable	The study does not involve clinical research, animal work nor systematic review methodology. The manuscript follows EMBO Press editorial policies, with complete reporting of experimental design, biological replicates, statistical analyses, and source data.
For **tumor marker prognostic studies,** we recommend that you follow the **REMARK** reporting guidelines (see link list at top right). See author guidelines, under 'Reporting Guidelines'. Please confirm you have followed these guidelines.	Not Applicable	
For **phase II and III randomized controlled trials**, please refer to the **CONSORT** flow diagram (see link list at top right) and submit the CONSORT checklist (see link list at top right) with your submission. See author guidelines, under 'Reporting Guidelines'. Please confirm you have submitted this list.	Not Applicable	

Data Availability

Data availability	Information included in the manuscript?	In which section is the information available? (Reagents and Tools Table, Materials and Methods, Figures, Data Availability Section)
Have **primary datasets** been deposited according to the journal's guidelines (see 'Data Deposition' section) and the respective accession numbers provided in the Data Availability Section?	Not Applicable	
Were **human clinical and genomic datasets** deposited in a public access-controlled repository in accordance to ethical obligations to the patients and to the applicable consent agreement?	Not Applicable	
Are **computational models** that are central and integral to a study available without restrictions in a machine-readable form? Were the relevant accession numbers or links provided?	Not Applicable	
If publicly available data were reused, provide the respective **data citations in the reference list**.	Not Applicable	
